# Molecular-topology-guided multiple-resonance emitters with sub-10-nm bandwidths: ultranarrow high-efficiency solution-processable OLEDs

**DOI:** 10.1093/nsr/nwag377

**Published:** 2026-06-22

**Authors:** Tao Hua, Nengquan Li, Xiaosong Cao, Cheng Zhong, Jingsheng Miao, Zhongyan Huang, Xiaojun Yin, Shaolong Gong, Zhanxiang Chen, Youming Zhang, Chuluo Yang

**Affiliations:** Shenzhen Key Laboratory of New Information Display and Storage Materials, College of Materials Science and Engineering, Shenzhen University, Shenzhen 518060, China; Institute of Technology for Future Industry, School of Science and Technology Instrument Application Engineering, Shenzhen University of Information Technology, Shenzhen 518172, China; Shenzhen Key Laboratory of New Information Display and Storage Materials, College of Materials Science and Engineering, Shenzhen University, Shenzhen 518060, China; Institute of Technology for Future Industry, School of Science and Technology Instrument Application Engineering, Shenzhen University of Information Technology, Shenzhen 518172, China; Shenzhen Key Laboratory of New Information Display and Storage Materials, College of Materials Science and Engineering, Shenzhen University, Shenzhen 518060, China; College of Chemistry and Molecular Sciences, Wuhan University, Wuhan 430072, China; Shenzhen Key Laboratory of New Information Display and Storage Materials, College of Materials Science and Engineering, Shenzhen University, Shenzhen 518060, China; Shenzhen Key Laboratory of New Information Display and Storage Materials, College of Materials Science and Engineering, Shenzhen University, Shenzhen 518060, China; Shenzhen Key Laboratory of New Information Display and Storage Materials, College of Materials Science and Engineering, Shenzhen University, Shenzhen 518060, China; College of Chemistry and Molecular Sciences, Wuhan University, Wuhan 430072, China; Shenzhen Key Laboratory of New Information Display and Storage Materials, College of Materials Science and Engineering, Shenzhen University, Shenzhen 518060, China; Shenzhen Key Laboratory of New Information Display and Storage Materials, College of Materials Science and Engineering, Shenzhen University, Shenzhen 518060, China; Institute of Technology for Future Industry, School of Science and Technology Instrument Application Engineering, Shenzhen University of Information Technology, Shenzhen 518172, China; Shenzhen Key Laboratory of New Information Display and Storage Materials, College of Materials Science and Engineering, Shenzhen University, Shenzhen 518060, China

**Keywords:** organic light-emitting diodes, ultranarrow-band emission, high-throughput screening, multiple-resonance emitters, solution-processed electroluminescence

## Abstract

Ultranarrow-band emitters are essential for high-color-purity organic light-emitting diodes, but their discovery remains largely empirical because spectral narrowing is difficult to predict. Progress has been limited by inefficient synthesis-and-testing cycles and the lack of simple design rules linking molecular structure to excited-state relaxation. Here, we establish a predictive design framework for ultranarrow multiple-resonance emitters by treating molecular topology as an explicit handle on structural relaxation. High-throughput screening of B,N-doped triangulene fusion modes identifies an alternating fusion pattern that minimizes reorganization energy and yields a ground-state descriptor for spectral narrowing without explicit excited-state calculations. Guided by this framework, we synthesize sky-blue emitters with photoluminescence bandwidths down to 9.1 nm and solution-processed devices with 10.9-nm electroluminescence bandwidths and 38.1% external quantum efficiency.

## Teaser

Computer screening identifies organic light-emitting materials that set a new benchmark for color purity and efficiency, advancing ultrahigh-definition displays.

## INTRODUCTION

Organic light-emitting diodes (OLEDs) have shown broad application prospects in wearable displays, and human–computer interaction systems, owing to their advantages of flexibility, active light emission, and lightweight [1,2]. As OLED technology advances toward ultra-high-definition applications, the need for higher spectral purity has become increasingly acute [3,4]. Multiple-resonance thermally activated delayed fluorescence (MR-TADF) materials, exemplified by B,N-doped polycyclic frameworks, offer a promising route to address this challenge by combining high luminescence efficiency with intrinsically narrowband emission [5–9]. In these emitters, the frontier molecular orbitals (FMOs) are separated at the atomic scale, which weakens electron–vibration coupling and gives rise to narrow short-range charge-transfer (SRCT) emission, typically with full width at half maximum (FWHM) below 30 nm [10–14]. At the same time, their inherently small singlet–triplet energy gap (Δ*E*_ST_) also enables efficient harvesting of triplet excitons through reverse intersystem crossing (RISC) [15–19]. Although MR-TADF materials have advanced rapidly in color tuning and device efficiency, the structural factors that govern further spectral narrowing remain poorly understood, which limits the further development of ultranarrow-band emitters for next-generation displays.

An important empirical route toward narrower emission is π-extension of the MR-TADF framework [20–22]. Expanding the conjugated skeleton can often strengthen the multiple-resonance character and, in many cases, lower the reorganization energy (*λ*), thereby suppressing vibronic broadening. It can also decrease Δ*E*_ST_ and accelerate RISC, improving the prospects for highly efficient OLEDs with reduced efficiency roll-off [23–25]. Yet in these quasi-planar fused frameworks, π-extension generates a large number of distinct fusion patterns, and even subtle changes in connectivity can reshape the electronic structure, alter molecular rigidity and redirect the structural relaxation pathways that broaden emission [26–28]. A central challenge in the design of MR-TADF emitters is therefore to identify the molecular topologies that most effectively suppress excited-state relaxation. This remains difficult because the accessible topological space far exceeds what can be explored experimentally, making non-intuitive ultranarrow-band motifs easy to overlook and leaving molecular design heavily reliant on synthesis-and-testing cycles [29,30]. More fundamentally, there is still no predictive framework that links molecular topology to excited-state relaxation and spectral purity, nor simple descriptors that capture the propensity for spectral narrowing without recourse to costly excited-state calculations [31]. Addressing these gaps would move emitter design beyond empirical optimization and toward predictive, mechanism-guided discovery, while also deepening the understanding of the photophysical origin of emission narrowing.

Here, we establish a topology-guided design framework for ultranarrow-band MR-TADF emitters by mapping the dependence of reorganization energy on fused architecture across an enumerated space of DABNA-derived structures. We identify connection patterns that minimize structural relaxation and show that this behavior is encoded in a ground-state descriptor based on the excitation-induced bond-order response, allowing the tendency toward low *λ* to be inferred from ground-state electronic structure alone. Guided by these principles, we realize MR emitters that exhibit ultranarrow photoluminescence with sub-10-nm FWHM with near-unity photoluminescence quantum yield (Φ_PL_) and a top-tier RISC rate on the order of 10^7^ s^−1^ in toluene. These properties enable solution-processed OLEDs with ultranarrow electroluminescence (FWHM of 10.9 nm) and a peak external quantum efficiency (EQE) of 38.1%. More broadly, our results establish molecular topology as an explicit design variable for simultaneously controlling spectral purity and efficiency in OLED materials.

## RESULTS AND DISCUSSION

The screening space was constructed using DABNA [8] as the basic fusing unit, guided by two connection rules: (i) each pair of DABNA units is fused by a single benzene ring, and (ii) the *ortho*/*para* B–N arrangement around the heteroatom site is preserved during fusion. While these rules exclude some graphene-type molecules, they facilitate synthetically accessible scaffolds and promote a non-bonding-dominated MR-type electronic structure. The allowed connections were categorized into three linkage groups: centroid–centroid fusion (A), centroid–terminal fusion (B1, B2), and terminal–terminal fusion (C1, C2, C3). We generated distinct oligomers up to tetramers, including 6 dimers, 53 trimers, and 576 tetramers, and evaluated *λ* for each candidate from optimized S_0_ and S_1_ geometries. The dataset size is computationally feasible and provides a solid foundation for investigating structure–property relationships.

**Figure 1.**
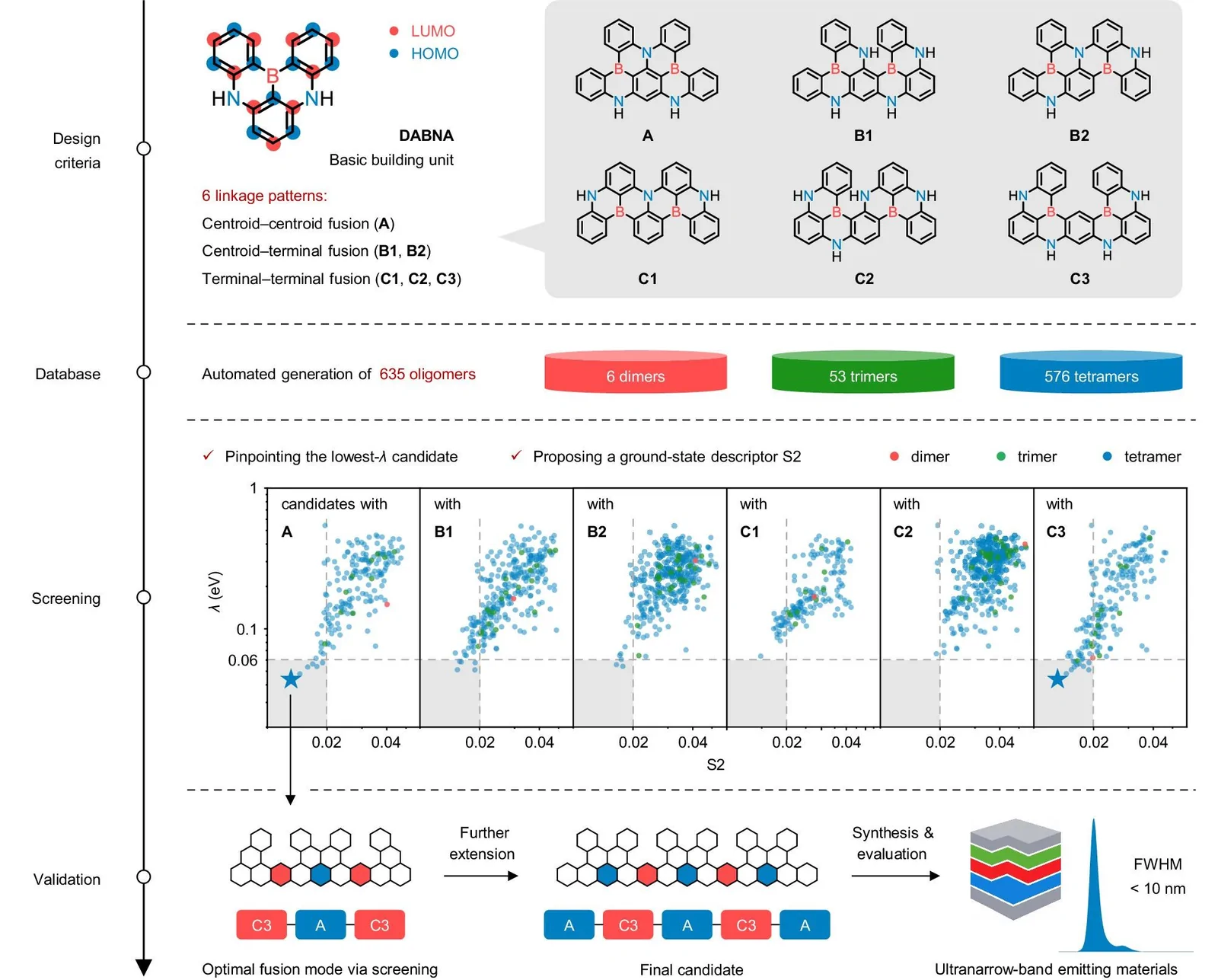
Overview of topology-guided theoretical screening of ultranarrow-band organic emitters.

The screening results, shown in Fig. 1, reveal two pivotal findings: (i) π-extension reduces the *λ* limit, suggesting that strategic extension promotes emission narrowing; (ii) *λ* depends strongly on the linkage pattern. At the dimer level, *λ* separates into favorable and unfavorable linkage families, where C3 gives the lowest *λ* of 62 meV (consistent with its role in *v*-DABNA [32]), and A gives the second-lowest *λ* of 149 meV, indicating that connectivity plays a dominant role in relaxation. This preference persists upon extension: the lowest-*λ* subset remains strongly enriched in C3 and A, and all screened structures with *λ* below 60 meV are dominantly composed of these two motifs. Importantly, the optimal pattern changes with length. Although the lowest-*λ* trimer adopts C3–C3, the lowest-*λ* tetramer switches to C3–A–C3. This crossover reflects the penalty of propagating consecutive C3 segments. Repeated C3–C3 connections induce helical distortions upon extension, where the resulting intramolecular stacking and enhanced non-planarity allow for larger structural relaxation during electronic transitions. Accordingly, the optimal tetramer topology incorporates A linkage to mitigate this helicity-driven relaxation.

To relate these relaxation trends to ground-state electronic structure, we introduced a simple descriptor that approximates excitation-induced bond reorganization. At the S_0_ geometry, we approximated an S_1_-like redistribution by removing one electron from the highest occupied molecular orbital (HOMO) and adding one electron to the lowest unoccupied molecular orbital (LUMO), and then recomputed Mayer bond orders. The resulting bond-order response reports how strongly the bonding network is perturbed by the excitation. We defined S2 = $\sum_i{| {\Delta {\mathrm{B}}{{\mathrm{O}}}_i} |}^2$ over all bonds in the conjugated skeleton, where ΔBO*_i_* is the bond-order change induced by this promotion. Smaller S2 values indicate that bond-order changes upon excitation are small and evenly distributed, reflecting delocalized electronic redistribution and stronger MR character. In the harmonic approximation, the reorganization energy can be expressed as: $\lambda \approx \frac{1}{2} \sum_{j}{k}_j{( {\Delta {Q}_j} )}^2$, where *k_j_* is the force constant and Δ*Q_j_* represents the nuclear displacement along vibrational mode *j* [33]. Of note, excitation-induced bond-order perturbations are correlated with the overall magnitude and localization of the nuclear displacement (Δ*Q_j_*) required to reach the relaxed excited-state geometry. Consequently, S2 serves as an electronic descriptor for the extent of structural relaxation. Across the enumerated candidates, *λ* shows an overall positive correlation with S2 (Fig. S31; Spearman correlation coefficient of  *ρ* = 0.646), which rationalizes the screening outcome: linkages enriched among the lowest-*λ* candidates generally present small bond-order changes upon promotion, consistent with a more spatially separated FMO distribution that weakens electron–vibration coupling.

It should be noted that the A linkage emerges as an outlier in the *λ*–S2 plot at the dimer level. Despite a relatively large S2 within its class, A maintains a small *λ* that is only slightly higher than C3. This deviation can be traced to the higher fusion degree of A, which stiffens the framework (i.e. for a similar bond-order redistribution, the more fused backbone undergoes a smaller geometric displacement, lowering *λ*). In higher oligomers, the influence of such mechanically atypical linkages becomes less distinct in the *λ*–S2 relation, as the contribution of any individual linkage is increasingly masked by cooperative effects across the extended framework. This interpretation also rationalizes the synergy between A and C3 in the lowest-*λ* architecture: the A linkage both breaks the C3–C3 propagation that builds helicity and increases local rigidity, reducing the structural response to a given electronic redistribution.

To further examine the broader applicability of S2, we evaluated 45 representative MR-TADF emitters reported in the literature (Fig. S32). The correlation between S2 and the experimental photoluminescence (PL) bandwidths gives an *R*^2^ value of 0.69, suggesting that smaller excitation-induced bond-order responses generally correspond to narrower emission. Nevertheless, S2 should be regarded as a preliminary screening descriptor rather than a definitive predictor. A small S2 value is favorable for narrow emission, but does not alone guarantee an ultranarrow spectrum, because insufficient molecular rigidity and additional structural-relaxation pathways beyond the HOMO–LUMO-dominated SRCT picture may also contribute to spectral broadening.

**Figure 2.**
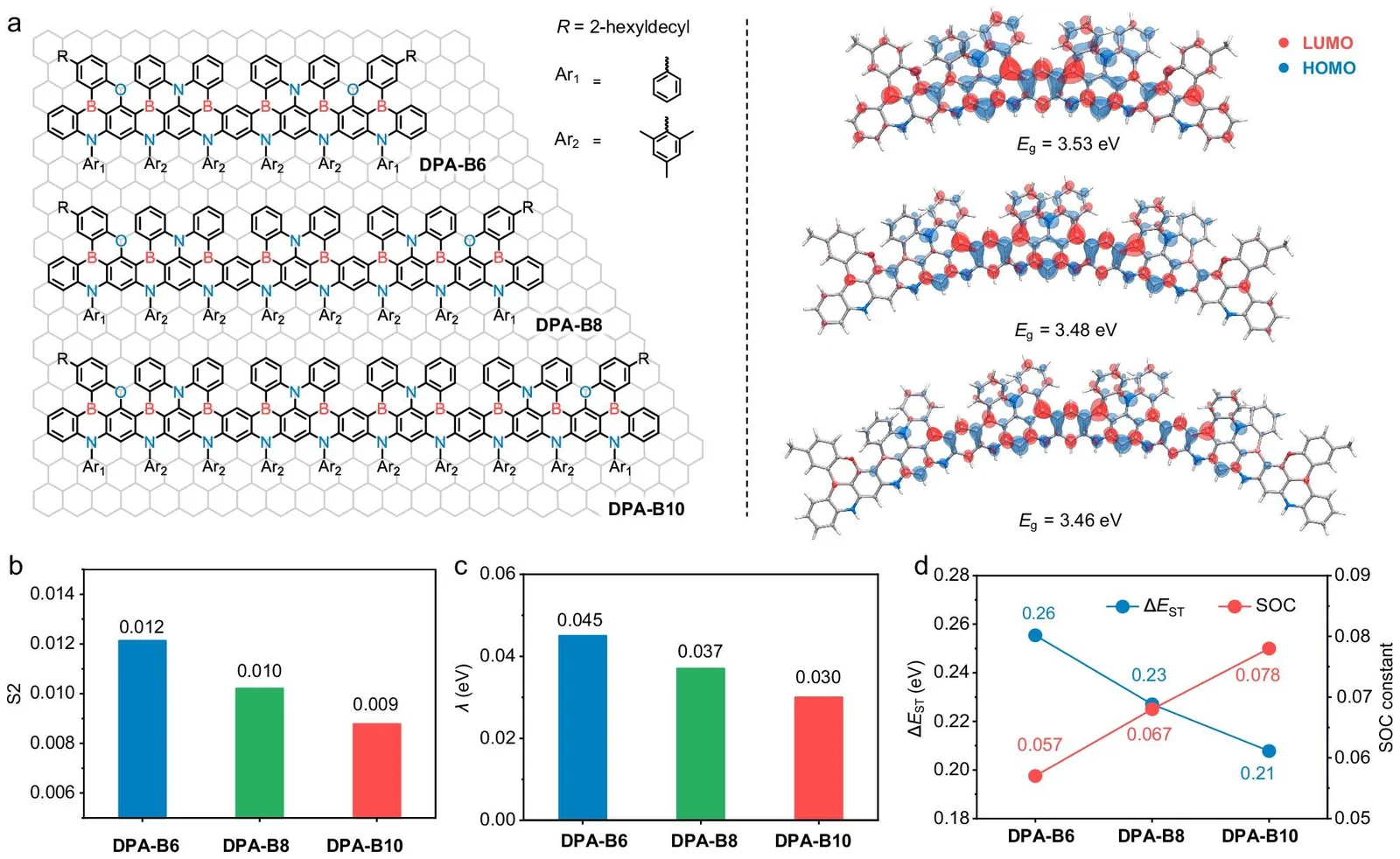
Molecular chemical structures and theoretical calculation of DPA-B6, DPA-B8, and DPA-B10. (a) Chemical structures of DPA-B*n* series (left) and FMOs distribution (right). (b) S2 values. (c) Reorganization energies. (d) Singlet–triplet energy splitting (Δ*E*_ST_) values and SOC constants.

Drawing on the screening results, we designed target emitters built on a linearly repeated C3–A linkage. To convert these topology-defined frameworks into synthetically accessible molecules, we capped them with B,N,O-doped triangulene end groups in a B2-type arrangement. This choice is supported by our previous study on the quadruple-borylated blue MR emitter DPA-B4 [6], which employs the same terminal motif and undergoes efficient one-shot borylation to form 12 C–B bonds in a single step (47% isolated yield, corresponding to 94% yield per C–B bond). This synthetic yield is notably high for multiply borylated systems derived from triarylamine precursors, possibly reflecting reduced side reactions when fewer reactive sites are exposed at the molecular termini. Moreover, oxygen contributes less to π-conjugation than nitrogen owing to its lower orbital energy, so the frontier electronic structure is expected to be less dominated by the terminal caps. On this basis, we derived a DPA-B*n* series (*n* = 6, 8, 10), where *n* denotes the number of boron atoms and the core incorporates *n*–2 DABNA units while retaining the same synthetically enabling caps (Fig. 2a). For practical implementation, 7-pentadecyl substituents were introduced to improve solution processability, whereas peripheral 2,4,6-trimethylphenyl groups were incorporated to prevent overborylation and suppress intermolecular interactions [34].

DFT calculations show that the FMO electron density of the DPA-B*n* series is more concentrated in the molecular center and progressively attenuated toward the termini (Fig. 2b). Consistent with this picture, bond-order changes upon excitation are largest within the C3–A-type segments. As π-extension increases, more bonds participate in the redistribution, but the magnitude of individual bond perturbations decreases, leading to a monotonic drop in S2 from 0.012 (DPA-B6) to 0.010 (DPA-B8) and 0.009 (DPA-B10), accompanied by a decrease in *λ* from 0.045 eV (DPA-B6) to 0.037 (DPA-B8) and 0.030 eV (DPA-B10). By contrast, DPA-B4 lacks the C3 substructure and exhibits substantially larger values of S2 (0.018) and *λ* (0.065 eV) (Figs S33 and S34), pointing to an abrupt improvement once C3 is introduced in DPA-B6. Therefore, these extended, topology-optimized π-skeletons are expected to deliver exceptional spectral purity relative to existing organic chromophores.

At the same time, the repetition of the C3–A linkage facilitates triplet-to-singlet upconversion process from two aspects. The Δ*E*_ST_ values decrease from 0.26 eV (DPA-B6) to 0.21 eV (DPA-B10) (Fig. 2d), consistent with trends reported by Pershin and co-workers [29]. It is crucial to point out that this reduction in exchange interactions is driven by the same mechanism that reduces S2 and *λ*, namely, the increased delocalization of the excitons across the π-skeleton. In parallel, spin–orbit coupling (SOC) is enhanced through the cumulative incorporation of helicene segments, with 〈S_1_|$\hat{H}$_SOC_|T_1_〉 increasing from 0.057 to 0.078 cm^−1^ across the series. This improvement is attributed to the greater out-of-plane character of the transitions, which eases angular-momentum limitations and accelerates spin mixing. Together, the concurrent reduction of Δ*E*_ST_ and increase in SOC provide a clear physical basis for accelerated RISC within a Fermi’s golden rule framework, *k*_RISC_ ∝ 〈S_1_|$\hat{H}$_SOC_|T_1_〉^2^/Δ*E*_ST_, which is key for efficient triplet exciton utilization in devices [35].

Subsequently, the DPA-B*n* series was synthesized using a modular route. As outlined in Schemes S1–S3, the key arylamine precursors were assembled through sequential Buchwald–Hartwig aminations and then converted to the target emitters by a one-shot multiple borylation at 180°C using boron triiodide (BI_3_) as the borylating reagent and 2,6-di(*tert*-butyl)pyridine as a proton scavenger. The final borylation step delivers these emitters in reasonable isolated yields of 12%–21% despite their substantial structural complexity, providing a family of highly elaborate organoboron architectures.

**Figure 3.**
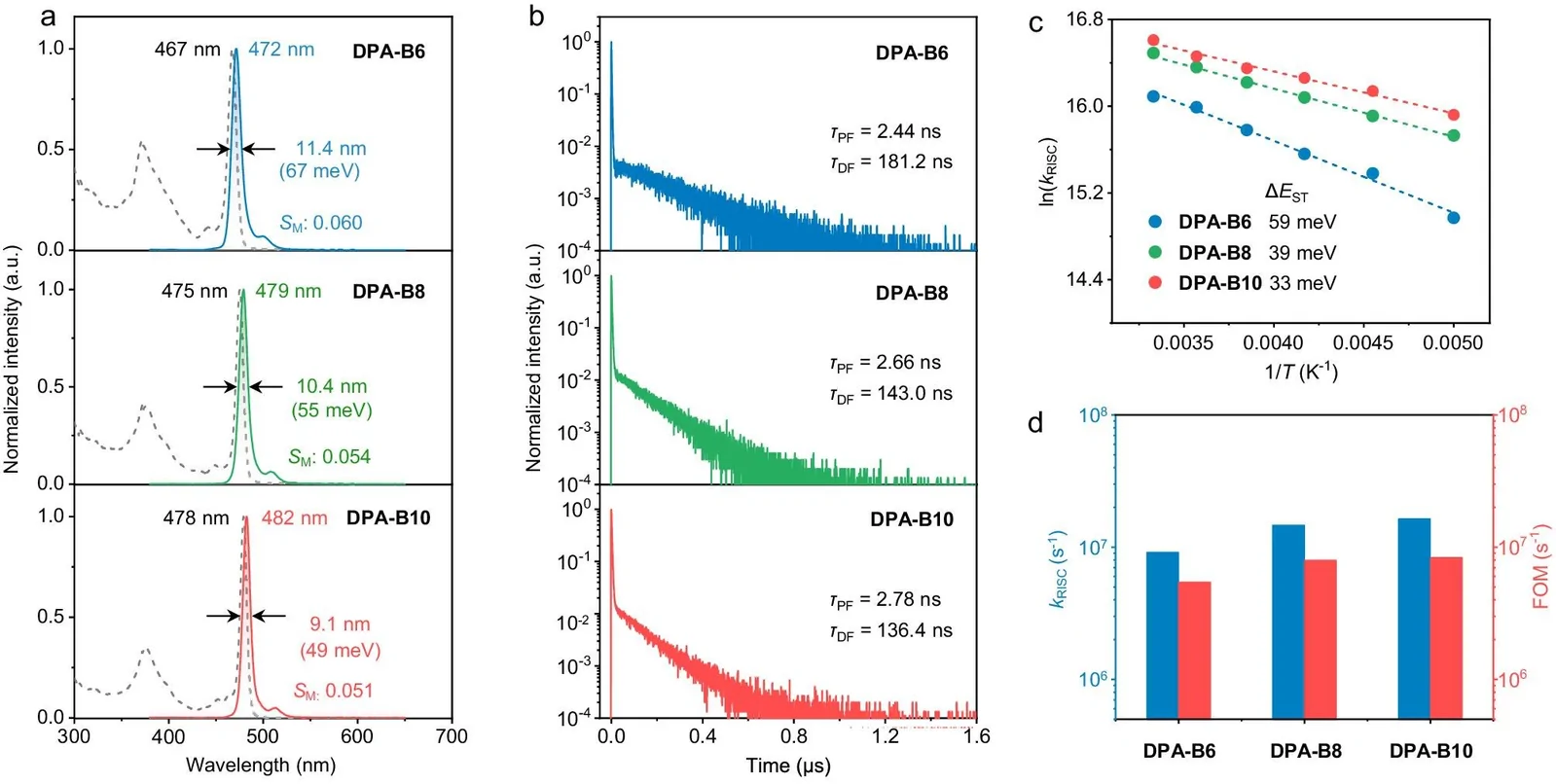
Photophysical properties of DPA-B6, DPA-B8, and DPA-B10 in toluene solution. (a) UV–vis and fluorescence spectra. (b) TRPL decay curves. (c) Arrhenius plots of *k*_RISC_. (d) FOM and *k*_RISC_ values at 300 K.

In dilute toluene solution (1 × 10^−5^ M) at room temperature (Fig. 3), the steady-state absorption spectra show sharp lowest-energy bands at 467 nm (DPA-B6), 475 nm (DPA-B8), and 478 nm (DPA-B10), consistent with the characteristic SRCT transition of MR emitters. Upon photoexcitation, the series exhibits sky-blue fluorescence with emission maxima that gradually redshift from 472 to 482 nm as π-extension increases. Importantly, the emission bands are ultranarrow and further contract across the series, with FWHM values of 11.4 nm (DPA-B6), 10.4 nm (DPA-B8), and 9.1 nm (DPA-B10). The corresponding Huang–Rhys factors (*S*_M_), estimated from the *I*_0–1_/*I*_0–0_ peak-height ratio, decrease from 0.060 to 0.054 and 0.051, reflecting weak vibronic coupling between S_1_ and S_0_ [36]. Upon cooling to 77 K, the FWHM values further narrow to 9.4–8.1 nm (Fig. S35). These results show that linear π-extension with an alternating fusion pattern robustly weakens excitation-induced structural deformation and suppresses vibrational broadening, yielding DPA-B10 as the first organic emitter to show an emission spectrum with FWHM < 10 nm and *S*_M_ < 0.1 at room temperature. Furthermore, the emitters show weak solvatochromic shifts (Fig. S37), which is expected to reduce sensitivity to solid-state solvation effects and help preserve high spectral purity in devices [37].

To probe excited-state dynamics, time-resolved photoluminescence (TRPL) decays were measured in deoxygenated toluene, revealing biexponential kinetics assigned to prompt and delayed fluorescence with clear temperature dependence. At 300 K, the prompt/delayed lifetimes are 2.44/181.2 ns (DPA-B6), 2.66/143.0 ns (DPA-B8), and 2.78/136.4 ns (DPA-B10), where the delayed component is quenched upon exposure to air, confirming its triplet-involved origin (Fig. S40) [38]. The hundred-nanosecond delayed lifetimes are significantly shorter than those of most TADF emitters, which typically exhibit microsecond delayed emission [39–42]. Together with near-unity Φ_PL_ yields in oxygen-free toluene (98%–100%), these emitters exhibit some of the fastest RISC rate constants (*k*_RISC_) among all heavy-element-free TADF systems, ranging from 0.98 × 10^7^ s^−1^ (DPA-B6) to 1.63 × 10^7^ s^−1^ (DPA-B10), while the radiative rate constants (*k*_r_) remain on the order of 10^8^ s^−1^. To better link molecular kinetics with device-relevant performance, we further evaluated a recently proposed figure of merit (FOM), ${k}_{\mathrm{r}}{k}_{{\mathrm{eq}}} = \frac{{4{k}_{\mathrm{r}}{k}_{{\mathrm{RISC}}}}}{{3{k}_{\mathrm{r}} + 4{k}_{{\mathrm{ISC}}}}}$, where *k*_ISC_ is the intersystem crossing rate constant [43]. The resulting values increase from 5.4 × 10^6^ to 7.9 × 10^6^ and 8.3 × 10^6^ s^−1^ for DPA-B6, DPA-B8, and DPA-B10, respectively, which are also among the highest values reported for organic emitters. Thus, triplet excitons in the DPA-B*n* series are anticipated to efficiently convert to light, rather than dissipating energy through multiple spin-flip cycles, thereby minimizing energy losses. Upon cooling to 200 K, the delayed component decreases gradually (Fig. S41), further supporting the TADF mechanism. The temperature dependence of *k*_RISC_ was analyzed using Arrhenius equation, yielding linear plots of ln(*k*_RISC_) versus 1/*T* with slopes corresponding to Δ*E*_ST_/*k*_B_, in which *k*_B_ is the Boltzmann constant [44]. The extracted Δ*E*_ST_ values decrease systematically from 59 meV (DPA-B6) to 39 meV (DPA-B8) and 33 meV (DPA-B10) (Fig. 3c). This trend correlates with the enhanced *k*_RISC_ and is consistent with the DFT results (Fig. 2d).

**Figure 4.**
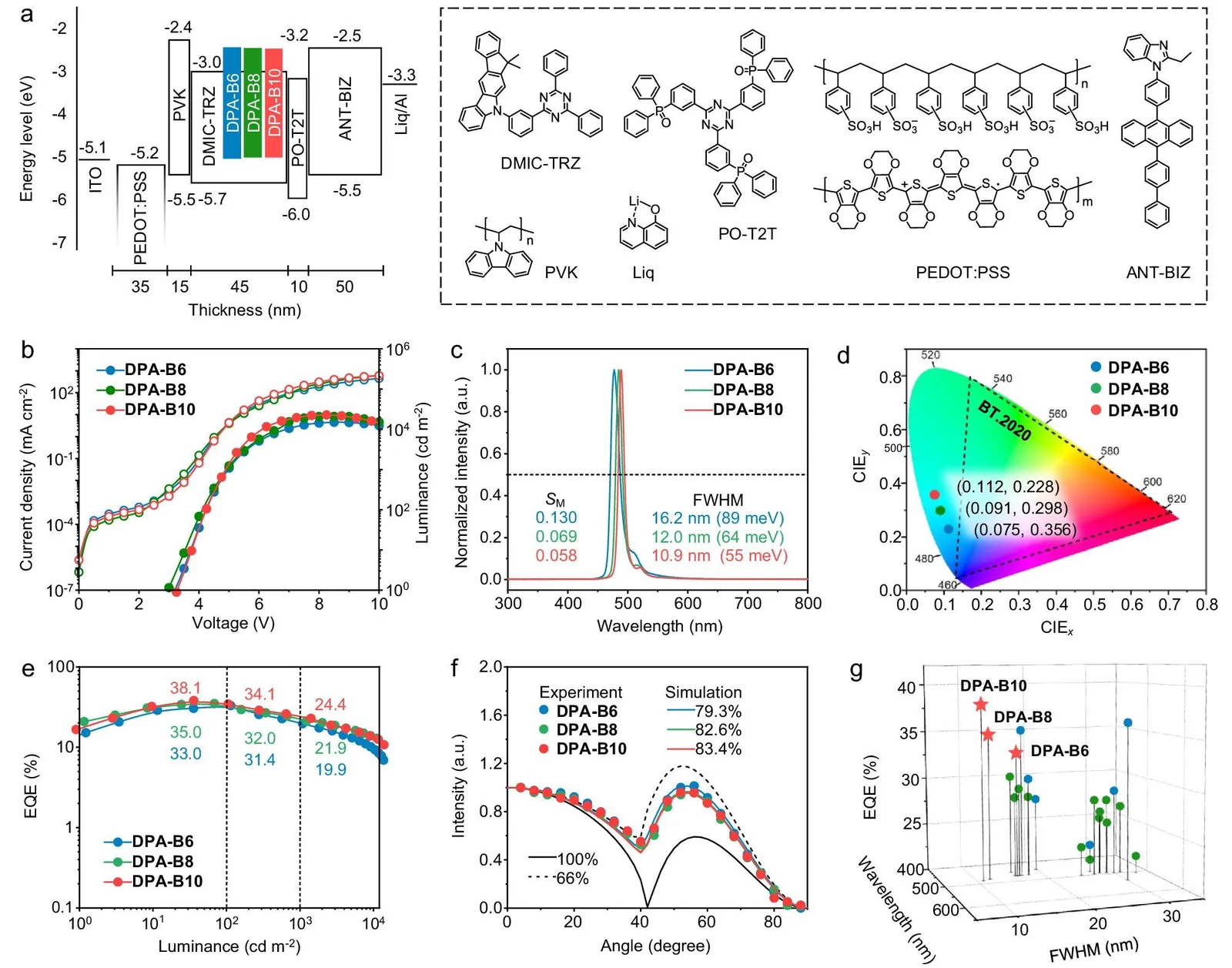
EL performance of OLEDs based on DPA-B*n*. (a) Device structure with the energy-level diagrams and chemical structures used for the functional layers. (b) Current density and luminance versus driving voltage characteristics. (c) EL spectra. (d) CIE coordinates. (e) EQE-luminance curves. (f) Angle-dependent *p*-polarized photoluminescence curves. (g) Representative solution-processed OLEDs based on MR-TADF emitters.

Motivated by the exceptional photoluminescence properties of the DPA-B*n* series and good solubility in common organic solvents, we fabricated solution-processed OLEDs using a multilayer architecture: ITO/poly(3,4-ethylenedioxythiophene):polystyrene sulfonate (PEDOT:PSS, 35 nm)/poly(N-vinylcarbazole (PVK, 10 mg/mL, 15 nm)/1,3-dihydro-1,1-dimethyl3-(3-(4,6-diphenyl-1,3,5-triazin-2-yl)phenyl)indeno-[2,1-*b*]carbazole (DMIC-TRZ):0.6 wt% emitters (45 nm)/1,3,5-triazine-2,4,6-triyl) tris(benzene-3,1-diyl) tris(diphenylphosphine oxide (PO-T2T, 10 nm)/(1-(4-(10-([1,1′-biphenyl]4-yl)anthracen-9-yl) phenyl)-2-ethyl-1H-benzo[*d*]-imidazole (ANT-BIZ, 50 nm)/8-hydroxyquinolinolato-lithium (Liq, 2 nm)/Al (100 nm). The dopant concentration was optimized through concentration-dependent screening, and 0.6 wt% was selected as the optimal loading for efficiency and spectral purity (Fig. S45). Energy-level diagrams, molecular structures, and performance metrics are presented in Fig. 4 and Figs S46–S48, with key parameters summarized in Table 1.

**Table 1.** Summary of the electroluminescence data of OLEDs.

Device	*λ*_EL_^a^ (nm)	*L*_max_^b^ (cd m^−2^)	CE^c^ (cd A^−1^)	PE^d^ (lm W^−1^)	EQE_max_^e^ (%)	EQE_100/1000_^f^ (%)	FWHM^g^ (nm)	CIE coordinates^h^ (*x, y*)
DPA-B6	477	15 010	42.9	31.7	33.0	31.4/19.9	16.2	[0.075, 0.356]
DPA-B8	485	21 639	48.8	39.8	35.0	32.0/21.9	12.0	[0.091, 0.298]
DPA-B10	489	21 492	58.0	45.1	38.1	34.1/24.4	10.9	[0.112, 0.228]

a Peak wavelength

b Maximum luminance

c Maximum current efficiency

d Maximum power efficiency

e Maximum external quantum efficiency

f External quantum efficiency at 100 and 1000 cd m^−2^

g FWHM values

h CIE coordinates

The electroluminescence (EL) spectra for all devices show negligible voltage-dependent shifts, indicating stable emission characteristics across the operating range (Fig. S47). The current density–voltage–luminance (*J–V–L*) characteristics reveal low turn-on voltages of 3.0–3.2 V and running voltages of 4.9–5.0 V at a practical luminance of 1000 cd m^−2^, reflecting efficient charge injection and balanced carrier transport enabled by the bipolar DMIC-TRZ host. This is also supported by the smooth and uniform film morphology, as indicated by atomic force microscopy (AFM) imaging with small *R*_q_ values around 0.5 nm for the emitting layers (Fig. S44). Consistent with their PL profiles, the devices display sky-blue emission with peaks centered at 477, 485, and 489 nm for DPA-B6, DPA-B8, and DPA-B10, respectively. The spectra are extremely clean, with corresponding FWHM values of 16.2, 12.0, and 10.9 nm, and *S*_M_ values of 0.130, 0.069, and 0.058, respectively. These FWHM values lie among the narrowest reported for both solution-processed and vacuum-evaporated bottom-emitting OLEDs (Tables S5 and S6), and are considerably narrower than typical emission bandwidths of microLED and quantum-dot LED platforms [45–47]. Notably, the CIE coordinates shift from (0.112, 0.228) to (0.075, 0.356), placing the emission outside the BT.2020 gamut triangle in the blue–cyan sector and gradually closer to the spectral locus (Fig. 4d). This feature is particularly relevant to emerging multi-primary display architectures in which a cyan channel is introduced alongside RGB to expand color gamut, suggesting that our emitters provide a compelling materials platform for next-generation wide-gamut displays [48].

Beyond spectral purity, the devices deliver outstanding efficiency (Fig. 4e). The maximum EQEs reach 33.0% (DPA-B6), 35.0% (DPA-B8), and 38.1% (DPA-B10), all achieved without any external optical outcoupling enhancement. In the benchmark compiled in Fig. 4g, these performances place our devices among the best solution-processed OLEDs. High current and power efficiencies are also obtained, reaching up to 42.9 cd A^−1^ and 31.7 lm W^−1^ (DPA-B6), 48.8 cd A^−1^ and 39.8 lm W^−1^ (DPA-B8), and 58.0 cd A^−1^ and 45.1 lm W^−1^ (DPA-B10), respectively. Importantly, device performance is highly reproducible: among 12 independently fabricated DPA-B10 devices, the EQE–luminance characteristics exhibit tight batch-to-batch dispersion (Fig. S48), supporting robust processability for solution processing. We attribute the high EQE to the synergistic contributions of balanced charge recombination, near-unity Φ_PL_, as well as favorable emitter orientation. Angle-dependent *p*-polarized PL measurements in the solution-processed film reveal pronounced horizontal dipole alignment, with the horizontal ratio (Θ_//_) increasing systematically from 79.3% (DPA-B6) to 83.4% (DPA-B10) (Fig. 4f), comparable to, and in some cases exceeding, those reported for mesogenic or dendritic emitters [49,50]. This trend is consistent with the progressively extended and rigidified π-skeleton, which increases molecular aspect ratio and promotes preferential in-plane alignment during film formation, thereby improving light extraction from the emissive layer. Moreover, owing to fast *k*_RISC_ and large FOM values, efficiency roll-off is effectively mitigated under practical operating conditions: the DPA-B10 device maintains 34.1% EQE at 100 cd m^−2^ and 24.4% at 1000 cd m^−2^, indicative of efficient triplet harvesting at high brightness. The remaining roll-off at high luminance may arise from device-level factors, including charge-transport imbalance and interfacial effects in the solution-processed multilayer architecture. Collectively, together with the ultranarrow emission bandwidth, these results demonstrate that spectral purity and device efficiency can be advanced simultaneously in solution-processed OLEDs, establishing the DPA-B*n* framework as a viable and scalable platform for next-generation printable OLED displays.

## CONCLUSION

In this study, we establish a topology-guided strategy for designing ultranarrow-band emitters for high-color-fidelity OLEDs. Through high-throughput screening of 635 molecular candidates constructed from the DABNA building block, we uncover a clear topology–relaxation rule: π-extension lowers the attainable limit of reorganization-energy *λ*, and *λ* is dominated by the fusion pattern. An alternating C3–A-type connectivity emerges as optimal, where C3 intrinsically minimizes structural relaxation, and A acts as a topological modifier that suppresses the helicity induced by consecutive C3 propagation. Importantly, *λ* across the surveyed space shows an overall positive correlation with a ground-state-derived descriptor based on excitation-induced bond-order response, indicating that the extent of excited-state relaxation can be estimated, to a useful degree, from ground-state electronic redistribution. This relationship provides a practical route to accelerate screening and rule extraction for narrowband emitters without relying exclusively on costly excited-state optimizations.

Guided by these rules, we design and develop the DPA-B*n* series, which validates the topology principle experimentally. The emitters deliver sky-blue photoluminescence with FWHM down to 9.1 nm, together with near-unity Φ_PL_, fast *k*_RISC_ on the order of 10^7^ s^−1^, and favorable horizontal dipole alignment up to 83.4% in the solution-processed film. These molecular advantages convert into solution-processed OLEDs with a peak EQE of 38.1%, while preserving ultranarrow electroluminescence with FWHM as small as 10.9 nm and minimum vibronic shoulder. Beyond record-level device performance, such intrinsic spectral purity can decrease reliance on microcavity engineering and complex optical stacks, and is well suited to multi-primary display concepts targeting color gamut beyond BT.2020 standard.

Looking ahead, combining topology enumeration with machine-learning models trained on physics-based descriptors should further accelerate the discovery of topologically distinct MR emitters, including deep-blue and longer-wavelength ultranarrow-band candidates. Overall, this work positions molecular topology as an explicit and programmable design variable for simultaneously advancing spectral purity and efficiency in scalable OLED technologies.

## METHODS

The detailed preparation and characteristic methods of materials are available as Supplementary data.

## Supplementary Material

nwag377_Supplemental_File

## Data Availability

The data that support the findings of this study are available from the corresponding author upon reasonable request.
