# Supplementary material for: Molecular-topology-guided multiple-resonance emitters with sub-10-nm bandwidths: ultranarrow high-efficiency solution-processable OLEDs
Source: Natl Sci Rev. 2026 Jun 22;13(16):nwag377. doi: 10.1093/nsr/nwag377 (PMC13520118; doi:10.1093/nsr/nwag377)
Supplement: Supplementary file 1 [file nwag377_supplemental_file.pdf]

Supplementary Data

Molecular-topology-guided multiple-resonance emitters with sub-10-nm bandwidths: ultranarrow high-efficiency solution-processable OLEDs

Tao Hua[1,2,†], Nengquan Li[1,2,†], Xiaosong Cao[1,*], Cheng Zhong[3,*], Jingsheng Miao[1], Zhongyan Huang[1], Xiaojun Yin[1], Shaolong Gong[3], Zhanxiang Chen[1], Youming Zhang[1,2], Chuluo Yang[1,*]

[1]Shenzhen Key Laboratory of New Information Display and Storage Materials, College of Materials Science and Engineering, Shenzhen University, Shenzhen 518060, China;

[2]Institute of Technology for Future Industry, School of Science and Technology Instrument Application Engineering, Shenzhen University of Information Technology, Shenzhen 518172, China and [3]College of Chemistry and Molecular Sciences, Wuhan University, Wuhan 430072, China

***Corresponding Authors.** E-mails: xcao@szu.edu.cn; zhongcheng@whu.edu.cn; clyang@szu.edu.cn

[†]Equally contributed to this work.

Contents

General Procedure S-3

Synthesis and Characterization S-3

Electrochemical Characterization..S-15

High-Throughput Screening..S-15

Quantum Chemical Calculations S-16

Analysis of Rate Constants S-16

Determination of Emitting Dipole Orientation S-17

Device Fabrication and Measurement S-17

Schemes, Figures and Tables S-19

References...S-77

General Procedure: All of the organic reactions were proceeded under the protection of the dry-argon atmosphere. ^{1}H NMR spectra were recorded at 400 or 500 MHz, and ^{13}C NMR spectra were recorded at 100 or 125 MHz NMR on Bruker instruments in $CDCl_3$. Tetramethylsilane was used as the internal standard and $CDCl_3$ as the solvent (Fig. S1-S28). The target products were further confirmed by Matrix-assisted laser desorption/ionization time of flight mass spectrometry (MALDI-TOF, Fig. S29). MALDI-TOF were performed on an Ultraflextreme apparatus (Bruker).

Synthesis and Characterization: The detailed procedures for the synthesis of DPA-B6, DPA-B8, and DPA-B10 are depicted in Scheme S1-S3. The borylated precursors were synthesized from commercially available starting materials using nucleophilic substitution reactions and Buchwald-Hartwig C-N coupling reactions. Intermediates 7[1] and 9[2] were synthesized according to the previously reported methods. The final products, DPA-B6, DPA-B8, and DPA-B10 were obtained in appreciable yields through a one-pot borylation Friedel-Crafts reaction at 200 °C. Methanol was added to the reaction mixture after the final one-pot multiple borylation reaction to precipitate the crude products, which were then collected by filtration. The crude products were purified by column chromatography to remove non-target byproducts, including possible incompletely borylated species, followed by recrystallization to afford the target DPA-B*n* emitters. The structures of DPA-B6, DPA-B8, and DPA-B10 were confirmed by ^{1}H NMR spectroscopy and MALDI-TOF mass spectrometry. The ^{1}H NMR spectra exhibited clean and well-resolved resonance patterns in both the aromatic and aliphatic regions. The apparent splitting or broadening of several resonances is likely caused by slowly interconverting structural conformers[3]. Since partially borylated products would lower the molecular symmetry and produce additional or split aromatic proton signals, the high-resolution ^{1}H NMR spectra are able to distinguish such non-target species if present in detectable amounts.

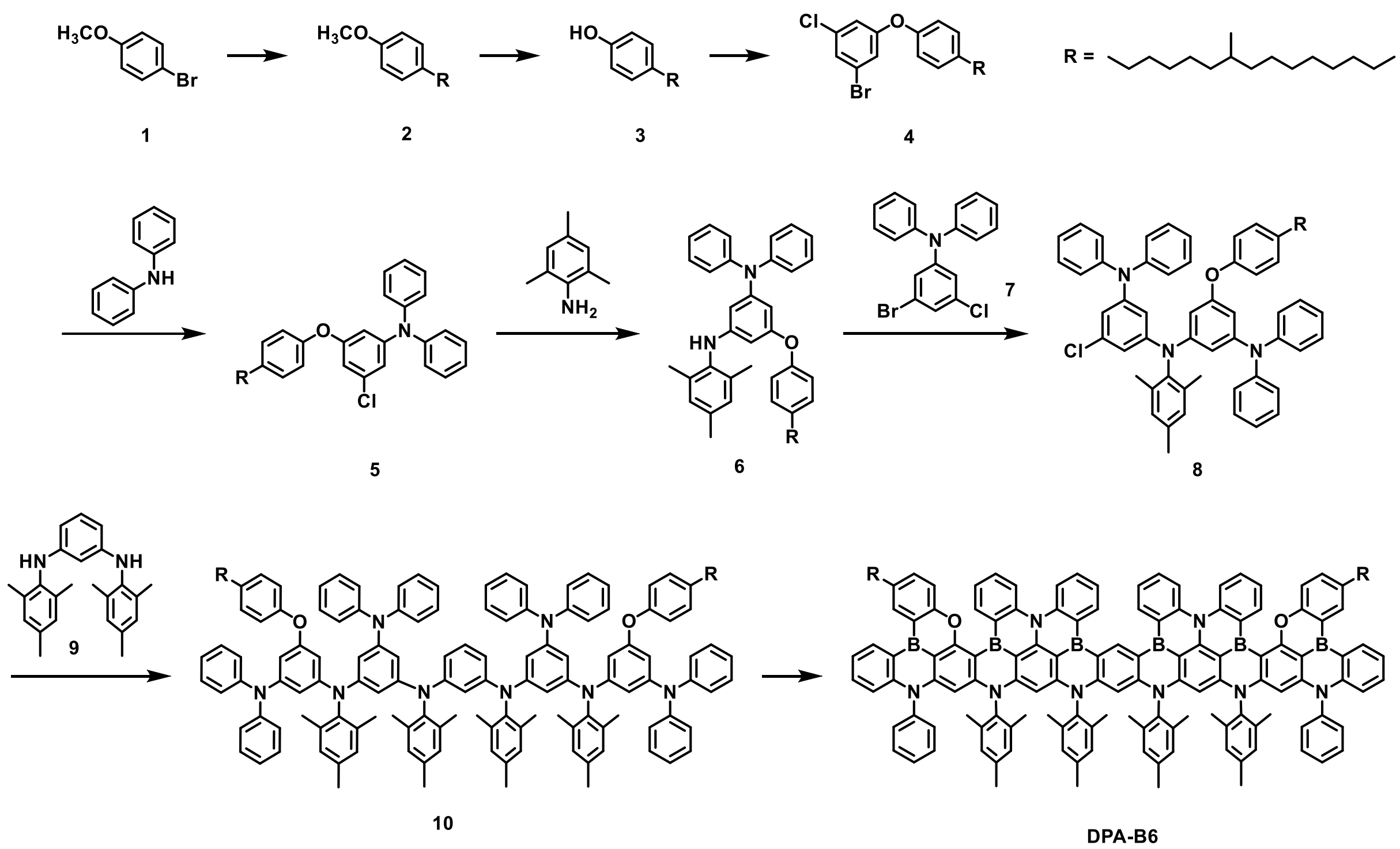

Scheme S1. Synthesis route of **DPA-B6**.

*Synthesis of 1-(2-hexyldecyl)-4-methoxybenzene (**2**)*

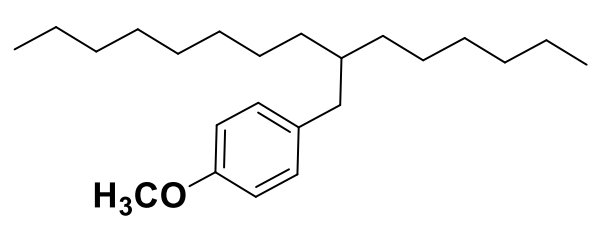

Under argon atmosphere, A solution of tert-butyllithium in hexane (48 mL, 1.3 M) was introduced drop wise to a solution of 1-bromo-4-methoxybenzene (5.6 g, 30 mmol) in 100 mL THF at -78 °C and the mixture was stirred for 2 hours, then a solution of 7-(bromomethyl)pentadecane (9.2 g, 30 mmol) in THF was added at -78 °C. The mixture was stirred for 1 hour, then gradually warmed to room temperature and further stirred for two days. The reaction was quenched with water and the organic layer was separated. The aqueous layer was extracted with dichloromethane (30 mL) for three times, the organic ingredients were dried over Na_2SO_4 for 15 min and removed by a rotatory evaporation. The crude product was purified by silica gel column chromatography, intermediate **2** was obtained as a colorless liquid (6.0 g, 60%). ^{1}H NMR (500 MHz, $CDCl_3$) δ 7.04 (d, *J* = 9.0 Hz, 2H), 6.81 (d, *J* = 8.5 Hz, 2H), 3.79 (s, 3H), 2.46 (d, *J* = 7.0 Hz, 2H), 1.57–1.48 (m, 1H), 1.33–1.12 (m, 24H), 0.93–0.75 (m, 6H). ^{13}C NMR (100 MHz, $CDCl_3$) δ 157.54, 133.92, 130.03, 113.47, 55.22, 39.79, 39.59, 33.11, 33.09, 31.93, 30.04, 29.72, 29.65, 29.36, 26.56, 22.70, 14.13.

*Synthesis of 4-(2-hexyldecyl)phenol (**3**)*

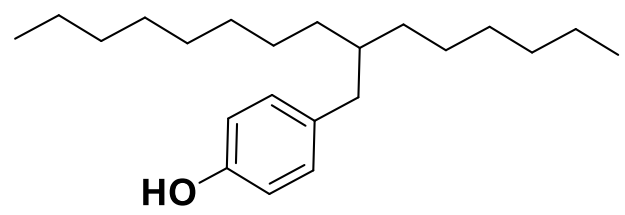

Under a dry atmosphere, compound **2** (5 g, 15 mmol) was dissolved in 50 mL ultra-dry dichloromethane solution, then BBr_3 (1.6 mL, 16.5 mmol) was slowly added into the above solution at 0 °C. The mixture was stirred for 30 min, then gradually warmed to room temperature and further stirred for 2 h. The reaction was quenched with methanol slowly. The mixture was extracted with dichloromethane and water for three times, the organic ingredients were dried over Na_2SO_4 for 15 min and removed by a rotatory evaporation. Compound **3** was obtained as a colorless liquid (4.2 g, 87%) and directly used to next step without further purification. ^{1}H NMR (400 MHz, $CDCl_3$) δ 7.00 (d, J = 8.4 Hz, 2H), 6.73 (d, J = 8.4 Hz, 2H), 4.56 (s, 1H), 2.44 (d, J = 7.2 Hz, 2H), 1.56–1.51 (m, 1H), 1.26–1.22 (m, 24H), 1.00–0.83 (m, 6H).

*Synthesis of 1-bromo-3-chloro-5-(4-(2-hexyldecyl)phenoxy)benzene (**4**)*

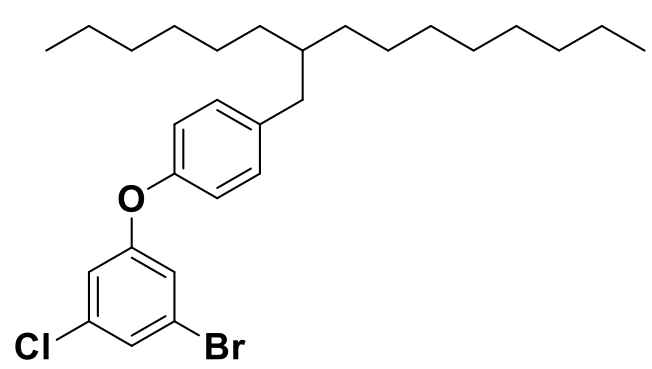

Compound **3** (3.2 g, 10 mmol), 1-bromo-3-chloro-5-fluorobenzene (2.1 g, 10 mmol), and Cs_2CO_3 (6.5 g, 20 mmol) were added into a 100 mL two necked round bottom flask. After 50 mL degassed N-methylpyrrolidone (NMP) was added into the flask, the reaction was allowed heating up to 180 ºC and stirred for 18 h. The reaction mixture was allowed to cool to room temperature and extracted with ethyl acetate (20 mL) for three times, the organic ingredients were dried over Na_2SO_4 for 15 min and removed by a rotatory evaporation. The crude product was purified by silica gel column chromatography, intermediate **4** was obtained as a colorless oil liquid (4.3 g, 86%). ^{1}H NMR (400 MHz, $CDCl_3$) δ 7.19 (t, J = 2.0 Hz, 1H), 7.14 (d, J = 8.4 Hz, 2H), 6.99 (d, J = 1.6 Hz, 1H), 6.93 (d, J = 8.4 Hz, 2H), 6.88 (t, J = 2.0 Hz, 1H), 2.52 (d, J = 6.8 Hz, 2H), 1.64–1.57 (m, 1H), 1.32–1.17 (m, 24H), 0.91–0.82 (m, 6H). ^{13}C NMR (100 MHz,

CDCl3) δ 159.47, 153.08, 138.58, 135.70, 130.71, 125.46, 122.93, 119.64, 119.33, 116.95, 39.92, 39.71, 33.17, 31.92, 31.89, 30.00, 29.66, 29.62, 29.34, 26.57, 26.54, 22.70, 22.69, 14.13.

*Synthesis of 3-chloro-5-(4-(2-hexyldecyl)phenoxy)-N,N-diphenylaniline (**5**)*

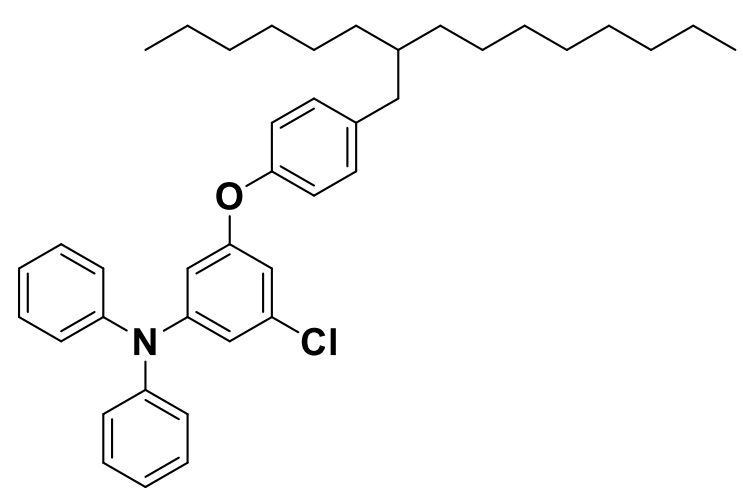

Under argon atmosphere, intermediate **4** (5.1 g, 10 mmol), diphenylamine (2.0 g, 12 mmol), sodium *tert*-butoxide (*t*-BuONa) (1.9 g, 20 mmol), *tri-tert*-butylphosphine tetrafluoroborate ($P(^tBu)_3HBF_4$) (290 mg, 1 mmol) and 100 mg palladium(II)acetate ($Pd(OAc)_2$) were added into a 100 mL two necked round bottom flask. After 30 mL degassed xylene was added into the flask, the reaction was allowed heating up to 100 ºC and stirred for 6 h. The reaction mixture was allowed to cool to room temperature and extracted with dichloromethane (25 mL) for three times, the organic ingredients were dried over Na_2SO_4 for 15 min and removed by a rotatory evaporation. The crude product was purified by silica gel column chromatography, intermediate **5** was obtained as a colorless oil liquid (5.1 g, 85%). ^{1}H NMR (400 MHz, $CDCl_3$) δ 7.31–7.26 (m, 3H), 7.13–7.03 (m, 9H), 6.92 – 6.86 (m, 2H), 6.69 (d, *J* = 2.0 Hz, 1H), 6.59 (t, *J* = 2.0 Hz, 1H), 6.47 (t, *J* = 2.0 Hz, 1H), 2.48 (d, *J* = 6.8 Hz, 2H), 1.61–1.55 (m, 1H), 1.32–1.16 (m, 24H), 0.91–0.84 (m, 6H). ^{13}C NMR (100 MHz, $CDCl_3$) δ 159.03, 153.93, 150.06, 146.85, 137.52, 135.18, 130.39, 129.65, 129.47, 125.57, 125.22, 123.91, 118.95, 116.56, 111.39, 110.65, 39.82, 39.69, 33.11, 31.93, 31.91, 30.02, 29.68, 29.65, 29.36, 26.56, 26.53, 22.70, 14.14.

*Synthesis of 5-(4-(2-hexyldecyl)phenoxy)-N^1-mesityl-N^3,N^3-diphenylbenzene-1,3-diamine (**6**)*

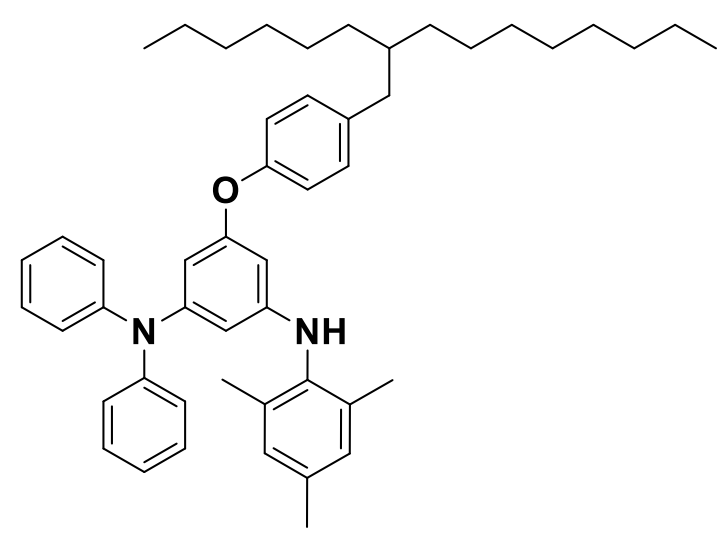

Under argon atmosphere, compound **5** (3.0 g, 5 mmol), 2,4,6-trimethylaniline (1.1 g, 7.5 mmol), *t*-BuONa (960 mg, 10 mmol), 2-dicyclohexylphosphino-2',6'-dimethoxybiphenyl (s-Phos) (145

mg, 0.5 mmol) and 100 mg tris(dibenzylideneacetone)dipalladium ($Pd_2(dba)_3$) were added into a 100 mL two necked round bottom flask. After 30 mL degassed xylene was added into the flask, the reaction was allowed heating up to 130 ºC and stirred for 6 h. The reaction mixture was allowed to cool to room temperature and extracted with dichloromethane (25 mL) for three times, the organic ingredients were dried over Na_2SO_4 for 15 min and removed by a rotatory evaporation. The crude product was purified by silica gel column chromatography, intermediate **6** was obtained as a colorless oil liquid (3.1 g, 89%). ^{1}H NMR (400 MHz, $CDCl_3$) δ 7.19 (d, *J* = 7.2 Hz, 4H), 7.08 (d, *J* = 8.4 Hz, 4H), 7.02 – 6.93 (m, 4H), 6.90–6.80 (m, 4H), 6.10 (d, *J* = 2.0 Hz, 1H), 5.98 (d, *J* = 2.0 Hz, 1H), 5.75 (d, *J* = 2.0 Hz, 1H), 4.99 (s, 1H), 2.44 (d, *J* = 7.2 Hz, 2H), 2.23 (s, 3H), 2.14 (s, 6H), 1.54–1.50 (m, 1H), 1.34–1.15 (m, 24H), 0.91–0.81 (m, 6H). ^{13}C NMR (100 MHz, $CDCl_3$) δ 158.84, 155.07, 149.70, 148.78, 147.57, 136.13, 135.78, 135.47, 135.07, 130.04, 129.13, 129.02, 124.43, 122.68, 117.96, 104.66, 104.17, 98.64, 39.68, 33.05, 31.93, 30.04, 29.70, 29.67, 29.36, 26.55, 26.51, 22.71, 20.85, 18.24, 14.14.

*Synthesis of 5-(4-(2-hexyldecyl)phenoxy)-N^1-mesityl-N^3,N^3-diphenylbenzene-1,3-diamine (**8**)*

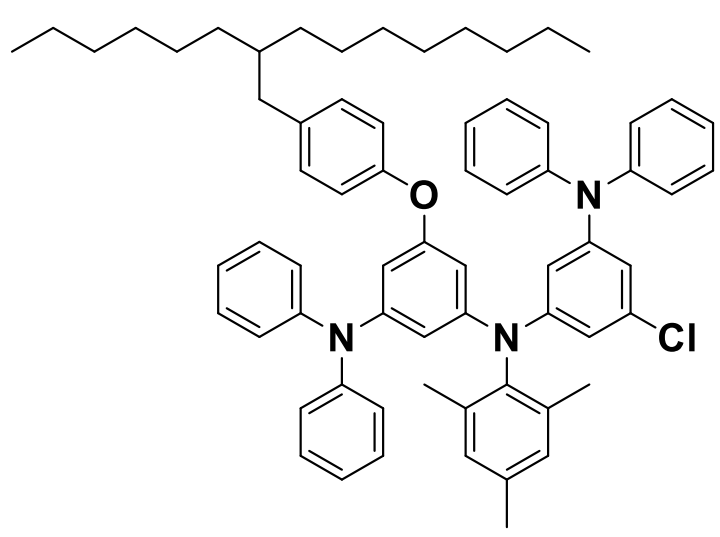

Under argon atmosphere, compound **6** (3.5 g, 5 mmol), 3-bromo-5-chloro-N,N-diphenylaniline (**7**) (1.1 g, 7.5 mmol), *t*-BuONa (960 mg, 10 mmol), P(^{t}Bu)$_3$HBF$_4$ (290 mg, 1 mmol) and 100 mg $Pd_2(dba)_3$ were added into a 100 mL two necked round bottom flask. After 30 mL degassed xylene was added into the flask, the reaction was allowed heating up to 100 ºC and stirred for 4 h. The reaction mixture was allowed to cool to room temperature and extracted with dichloromethane (25 mL) for three times, the organic ingredients were dried over Na_2SO_4 for 15 min and removed by a rotatory evaporation. The crude product was purified by silica gel column chromatography, intermediate **6** was obtained as a white solid (3.1 g, 89%). ^{1}H NMR (400 MHz, $CDCl_3$) δ 7.23–7.14 (m, 8H), 7.05–6.90 (m, 14H), 6.82–6.73 (m, 4H), 6.53 (d, *J* = 2.0 Hz, 1H), 6.44–6.42 (m, 1H), 6.42–6.38 (m, 2H), 6.17–6.13 (m, 2H), 2.43 (d, *J* = 6.8 Hz,

2H), 2.23 (s, 3H), 1.95 (s, 6H), 1.52–1.46 (m, 1H), 1.34–1.11 (m, 24H), 0.91–0.81 (m, 6H). ^{13}C NMR (101 MHz, CDCl3) δ 158.33, 154.84, 149.33, 147.38, 147.18, 147.00, 139.18, 136.94, 136.88, 136.23, 135.11, 130.09, 129.92, 129.31, 129.26, 129.14, 124.54, 124.29, 123.40, 123.26, 122.93, 117.70, 115.48, 113.13, 112.47, 110.30, 107.33, 105.32, 39.68, 33.04, 31.95, 30.05, 29.72, 29.68, 29.38, 26.55, 26.52, 22.72, 20.95, 18.40, 14.16.

*Synthesis of N^1,$N^{1\prime}$-(1,3-phenylene)bis(N^3-(3-(diphenylamino)-5-(4-(2-hexyldecyl)phenoxy) phenyl)-N^1,N^3-dimesityl-N^5,N^5-diphenylbenzene-1,3,5-triamine) (**10**)*

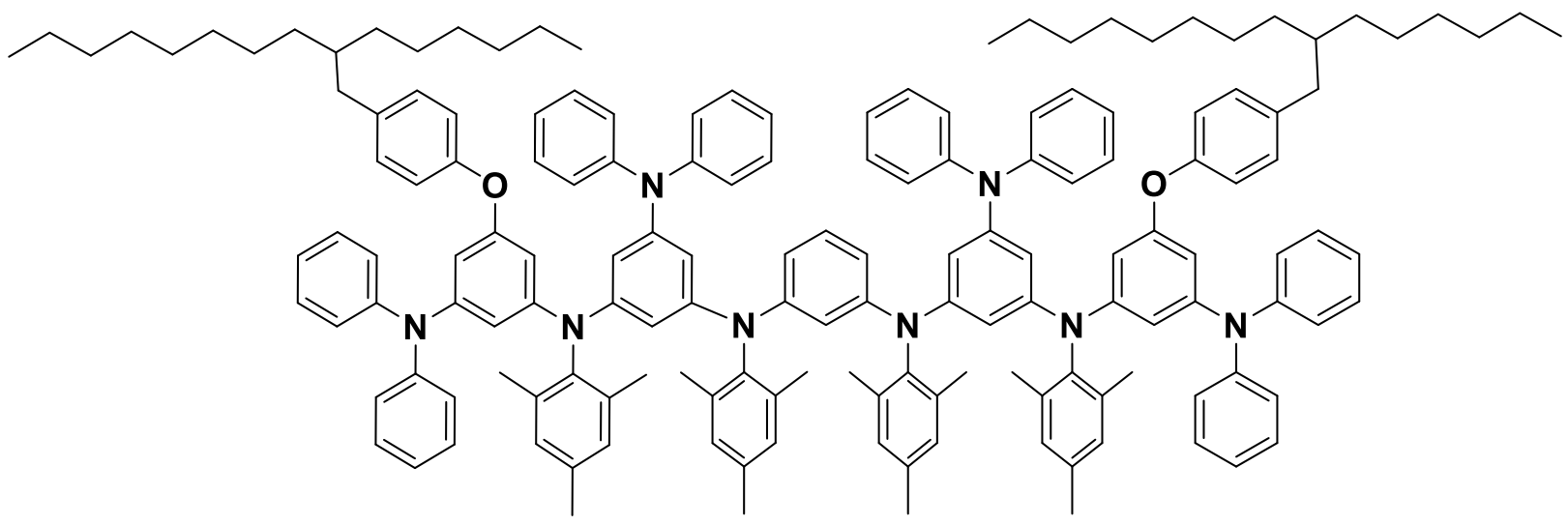

Under argon atmosphere, compound **8** (2.2 g, 2.2 mmol), N^1,N^3-dimesitylbenzene-1,3-diamine (**9**) (345 mg, 1 mmol), *t*-BuONa (384 mg, 4 mmol), s-Phos (82 mg, 0.2 mmol) and 92 mg $Pd_2(dba)_3$ were added into a 100 mL two necked round bottom flask. After 25 mL degassed xylene was added into the flask, the reaction was allowed heating up to 135 ºC and stirred for 3 h. The reaction mixture was allowed to cool to room temperature and extracted with dichloromethane (25 mL) for three times, the organic ingredients were dried over Na_2SO_4 for 15 min and removed by a rotatory evaporation. The crude product was purified by silica gel column chromatography, intermediate **10** was obtained as a white solid (1.4 g, 63%). ^{1}H NMR (400 MHz, $CDCl_3$) δ 7.17–7.03 (m, 16H), 6.93 (dd, *J* = 12.2, 8.1 Hz, 22H), 6.88–6.78 (m, 8H), 6.76–6.70 (m, 4H), 6.66–6.55 (m, 8H), 6.33 (s, 2H), 6.25–6.12 (m, 8H), 6.10–6.02 (m, 2H), 5.89–5.82 (m, 2H), 2.41 (d, *J* = 6.8 Hz, 4H), 2.17 (s, 12H), 1.81 (s, 12H), 1.61 (s, 12H), 1.52–1.45 (m, 2H), 1.34–1.10 (m, 48H), 0.90–0.83 (m, 12H). ^{13}C NMR (101 MHz, $CDCl_3$) δ 158.13, 155.13, 149.12, 148.30, 147.93, 147.39, 147.34, 146.98, 146.48, 145.95, 139.79, 136.85, 136.71, 136.18, 135.92, 135.85, 130.04, 129.51, 129.28, 129.16, 129.01, 128.87, 124.45, 123.99, 123.45, 123.03, 122.56, 122.07, 117.56, 111.78, 109.71, 109.59, 109.41, 107.26, 106.79, 104.67, 39.71, 39.67, 33.07, 31.97, 30.08, 29.74, 29.71, 29.40, 26.58, 26.54, 22.74, 20.95, 20.92, 18.21, 18.04, 14.19.

Synthesis of ***DPA-B6***

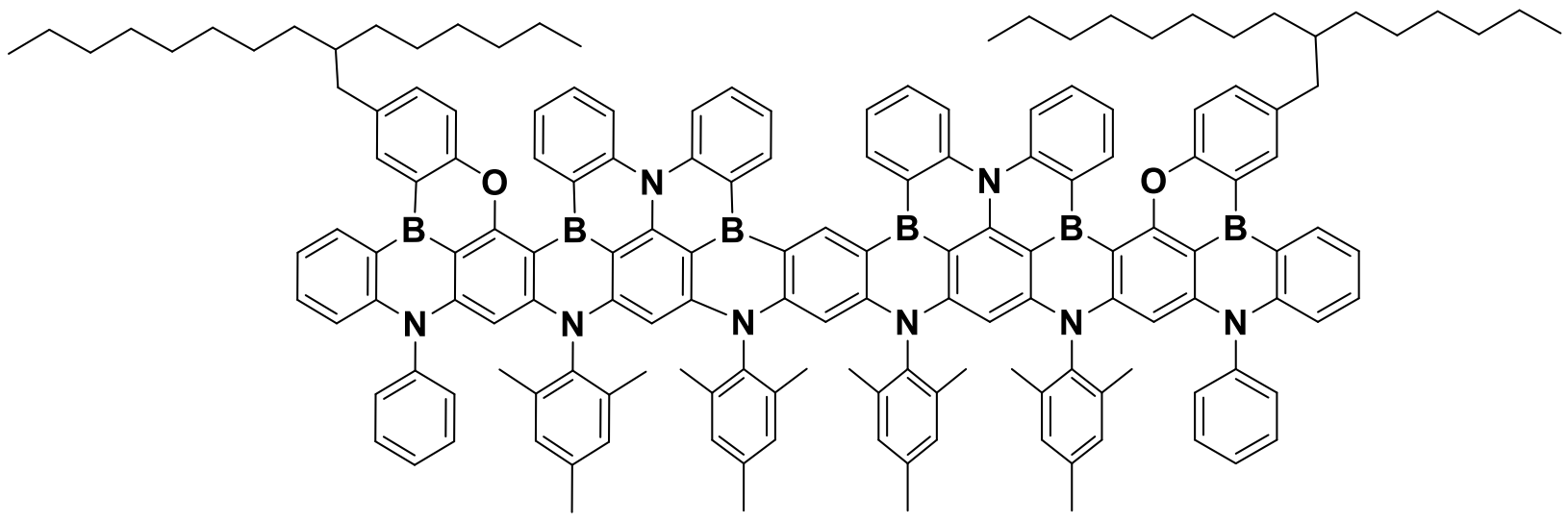

Under argon atmosphere, compound **10** (226 mg, 0.1 mmol) was dissolved in 2 mL ultradry *o*-dichlorobenzene. 2,6-di-tert-butylpyridine (0.4 mL, 1.8 mmol) and BI_3 (940 mg, 2.4 mmol) was injected into the mixture at argon atmosphere quickly. The reaction was allowed heating up to 200 ºC and stirred for 24 h. The reaction mixture was allowed to cool to room temperature and excess methanol was added into the system slowly in an ice-bath. Powdered solid was obtained by filtration followed by washing with methanol. The crude product was purified by silica gel column chromatography, **DPA-B6** was obtained as a pale yellow solid (335 mg, 21%). ^{1}H NMR (400 MHz, $CDCl_3$) δ 10.46 (d, *J* = 22.7 Hz, 1H), 9.41–9.31 (m, 1H), 9.02 (d, *J* = 8.0 Hz, 1H), 8.96–8.88 (m, 2H), 8.85–8.74 (m, 2H), 8.55–8.40 (m, 4H), 8.32–8.18 (m, 2H), 7.70–7.61 (m, 2H), 7.58 (d, *J* = 8.4 Hz, 3H), 7.51–7.39 (m, 5H), 7.36–7.22 (m, 10H), 7.16–7.09 (m, 2H), 7.06–6.83 (m, 4H), 6.72–6.58 (m, 2H), 6.55–6.29 (m, 8H), 5.81–5.70 (m, 1H), 5.31–5.17 (m, 2H), 5.1–5.00 (m, 2H), 2.72 (d, *J* = 5.8 Hz, 4H), 2.25–2.12 (m, 12H), 1.78–1.67 (m, 2H), 1.46–1.36 (m, 12H), 1.34–1.13 (m, 60H), 0.83–0.74 (m, 12H). MALDI-TOF (m/z): [M]+ calcd for $C_{158}H_{156}B6N_8O_2$, 2263.298; found, 2263.376

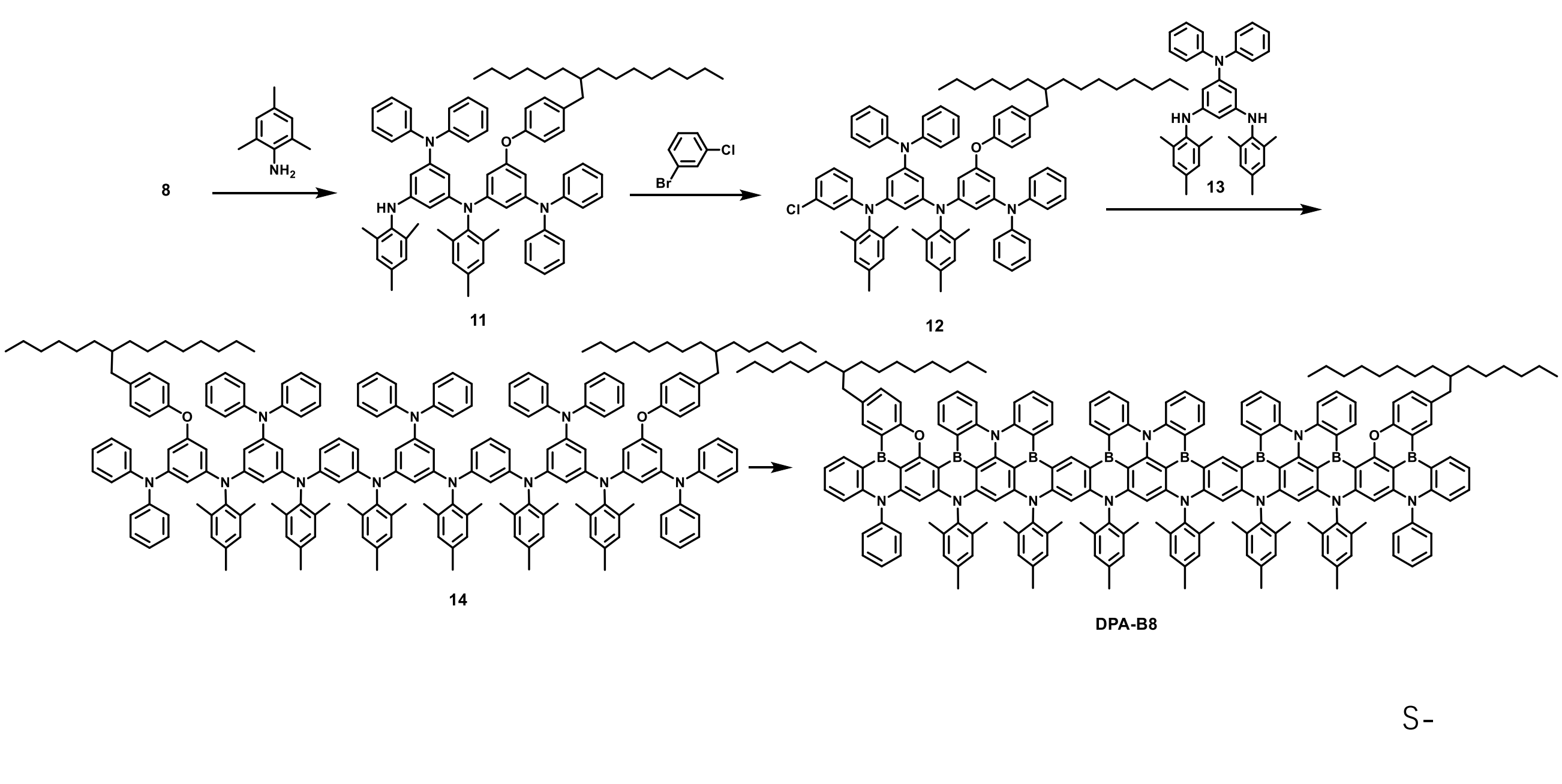

Scheme S2. Synthesis route of **DPA-B8**.

*Synthesis of N[1]-(3-(diphenylamino)-5-(4-(2-hexyldecyl)phenoxy)phenyl)-N[1],N[3]-dimesityl-N[5], N[5]-diphenylbenzene-1,3,5-triamine (**11**)*

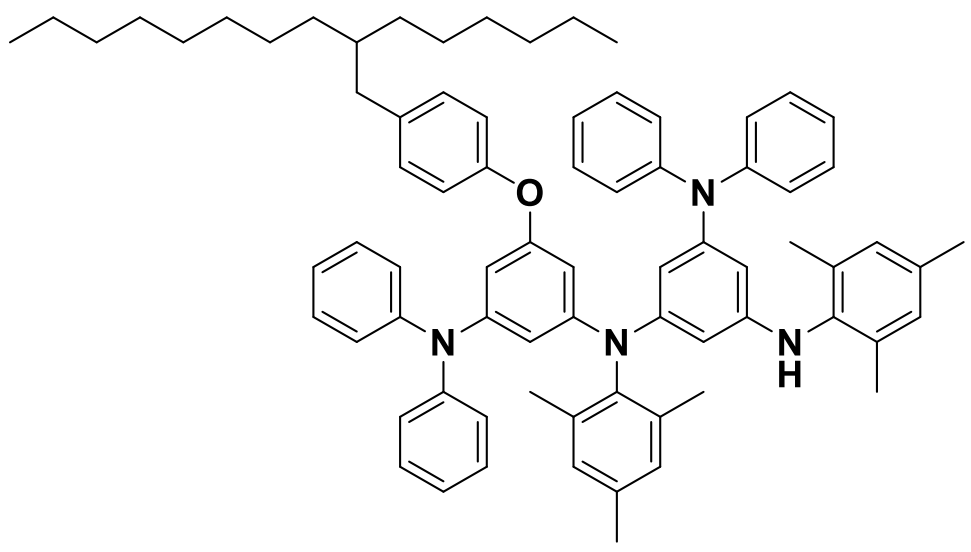

The synthesis procedure of compound **11** is similar to that of compound **6**, employing compound **8** instead of compound **5**. Compound **11** (86%) was acquired as white solid. ^{1}H NMR (400 MHz, $CDCl_3$) δ 7.20–7.08 (m, 8H), 7.02–6.94 (m, 10H), 6.94–6.83 (m, 4H), 6.75 (d, *J* = 6.4 Hz, 6H), 6.51–6.46 (m, 1H), 6.20–6.05 (m, 3H), 5.74–5.68 (m, 1H), 5.62–5.57 (m, 1H), 4.80 (s, 1H), 2.42 (d, *J* = 6.8 Hz, 2H), 2.22 (d, *J* = 8.4 Hz, 6H), 2.00 (s, 6H), 1.94 (s, 6H), 1.52–1.46 (m, 1H), 1.36–1.12 (m, 24H), 0.91–0.83 (m, 6H). ^{13}C NMR (125 MHz, $CDCl_3$) δ 158.08, 155.09, 149.04, 147.45, 147.43, 147.38, 139.88, 136.93, 136.35, 135.96, 135.36, 134.89, 130.01, 129.65, 129.15, 128.99, 128.96, 128.91, 128.85, 124.43, 124.10, 124.02, 123.91, 123.80, 123.75, 123.00, 122.60, 122.57, 122.36, 122.15, 117.51, 110.70, 107.23, 105.50, 77.30, 77.04, 76.79, 39.66, 39.63, 33.08, 32.70, 31.94, 29.72, 28.79, 26.54, 23.06, 22.71, 20.92, 20.83, 18.37, 18.17, 14.18, 14.15.

*Synthesis of N[1]-(3-chlorophenyl)-N[3]-(3-(diphenylamino)-5-(4-(2-hexyldecyl)phenoxy)phenyl)-N1,N3-dimesityl-N[5],N[5]-diphenylbenzene-1,3,5-triamine (**12**)*

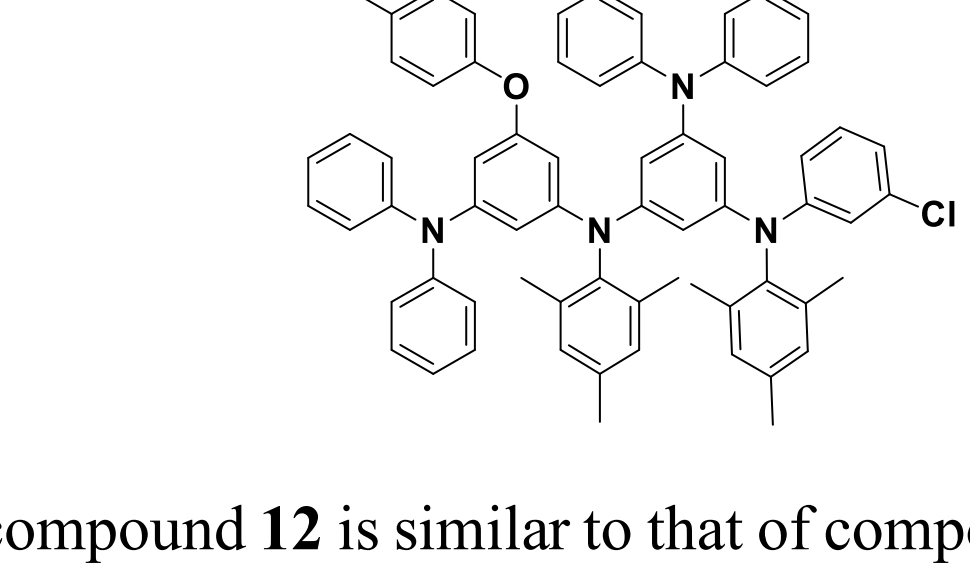

The synthesis procedure of compound **12** is similar to that of compound **8**, employing 1-bromo-3-chlorobenzene instead of compound **7**. Compound **12** (88%) was acquired as white solid. ^{1}H NMR (500 MHz, $CDCl_3$) δ 7.19–7.10 (m, 8H), 7.04–6.93 (m, 10H), 6.93–6.85 (m, 5H), 6.78–

6.74 (m, 4H), 6.73–6.70 (m, 1H), 6.69 (s, 2H), 6.67–6.59 (m, 2H), 6.38–6.34 (m, 2H), 6.24–6.17 (m, 2H), 6.09–6.06 (m, 1H), 5.94 (s, 1H), 2.43 (d, J = 6.4 Hz, 2H), 2.24 (s, 3H), 2.19 (s, 3H), 1.88 (s, 6H), 1.82 (s, 6H), 1.55–1.50 (m, 1H), 1.30–1.15 (m, 24H), 0.90–0.82 (m, 6H). ^{13}C NMR (100 MHz, $CDCl_3$) δ 158.08, 155.08, 149.16, 148.74, 147.77, 147.33, 147.29, 147.09, 146.78, 146.26, 139.60, 139.13, 137.05, 136.74, 136.68, 136.38, 135.98, 134.57, 130.03, 129.77, 129.71, 129.59, 129.17, 129.00, 124.55, 124.09, 123.77, 122.65, 122.41, 119.93, 118.17, 117.52, 116.64, 109.90, 109.74, 109.54, 107.51, 106.94, 104.89, 39.70, 39.64, 33.05, 31.95, 30.07, 29.73, 29.69, 29.38, 26.57, 26.53, 22.73, 20.99, 20.92, 18.20, 14.17.

Synthesis of N^1-(3-chlorophenyl)-N^3-(3-(diphenylamino)-5-(4-(2-hexyldecyl)phenoxy)phenyl)-N1,N3-dimesityl-N^5,N^5-diphenylbenzene-1,3,5-triamine ***(14)***

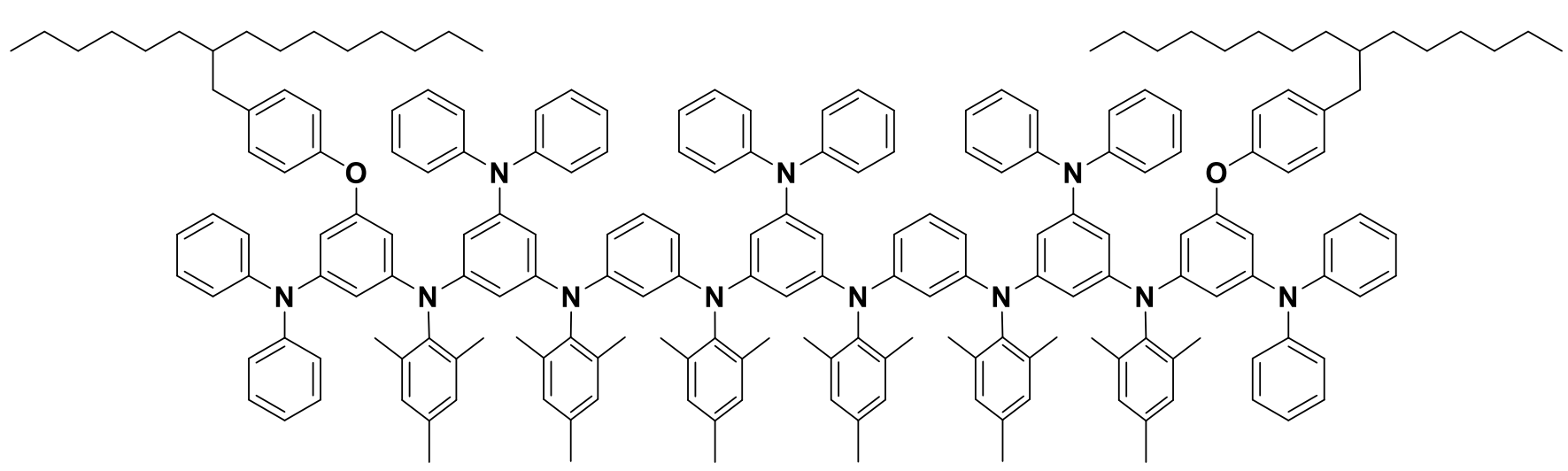

The synthesis procedure of compound **14** is similar to that of compound **10**, employing N^1,N^3-dimesityl-N^5,N^5-diphenylbenzene-1,3,5-triamine (**13**) and compound **12** instead of N^1,N^3-dimesitylbenzene-1,3-diamine and compound **8**, respectively. Compound **14** (60%) was obtained as white solid. ^{1}H NMR (400 MHz, $CDCl_3$) δ 7.19–7.01 (m, 20H), 6.98–6.84 (m, 28H), 6.83–6.76 (m, 6H), 6.73 (d, J = 8.4 Hz, 4H), 6.69–6.61 (m, 6H), 6.57 (d, J = 6.8 Hz, 8H), 6.32 (d, J = 2.0 Hz, 2H), 6.29–6.11 (m, 14H), 6.04 (d, J = 2.0 Hz, 2H), 5.84–5.73 (m, 3H), 2.41 (d, J = 6.8 Hz, 4H), 2.16 (d, J = 6.0 Hz, 18H), 1.81 (s, 12H), 1.64–1.56 (m, 24H), 1.51–1.46 (m, 2H), 1.32–1.09 (m, 48H), 0.91–0.82 (m, 12H). ^{13}C NMR (101 MHz, $CDCl_3$) δ 158.10, 155.12, 149.09, 148.25, 148.04, 147.96, 147.42, 147.40, 147.32, 147.00, 146.88, 146.44, 146.08, 145.84, 140.01, 139.77, 139.75, 136.87, 136.81, 136.72, 136.15, 135.90, 135.83, 135.75, 130.01, 129.48, 129.27, 129.25, 129.14, 128.98, 128.87, 128.81, 124.44, 123.98, 123.39, 122.54, 122.02, 121.98, 117.53, 111.49, 111.45, 109.84, 109.71, 109.62, 109.47, 109.07, 107.51, 107.11, 106.71, 104.58, 77.37, 77.06, 76.74, 39.69, 39.66, 33.05, 31.96, 30.07, 29.73, 29.70, 29.38, 26.57, 26.53, 22.73, 20.97, 20.93, 20.91, 18.21, 18.06, 17.99, 14.17.

Synthesis of ***DPA-B8***

The synthesis procedure of **DPA-B8** is similar to that of **DPA-B6**, employing precursor **14** instead of precursor **10**. **DPA-B8** (18%) was acquired as pale yellow solid. ^{1}H NMR (400 MHz, $CDCl_3$) δ 10.53 (d, J = 21.1 Hz, 2H), 9.46–9.35 (m, 2H), 9.16–9.05 (m, 2H), 8.99 (d, J = 6.6 Hz, 2H), 8.92 (d, J = 6.8 Hz, 2H), 8.60 (s, 2H), 8.55–8.43 (m, 3H), 8.41–8.33 (m, 2H), 8.29 (d, J = 9.2 Hz, 1H), 7.76–7.61 (m, 6H), 7.60–7.46 (m, 6H), 7.45–7.30 (m, 10H), 7.16–7.04 (m, 6H), 7.02–6.94 (m, 2H), 6.76 (d, J = 10.0 Hz, 2H), 6.65–6.45 (m, 12H), 5.91 (d, J = 2.8 Hz, 2H), 5.42–5.33 (m, 2H), 5.26–5.17 (m, 3H), 2.80 (d, J = 8.4 Hz, 4H), 2.37–2.15 (m, 24H), 1.87–1.74 (m, 2H), 1.54–1.47 (m, 30H), 1.48–1.19 (m, 48H), 0.96–0.84 (m, 12H).

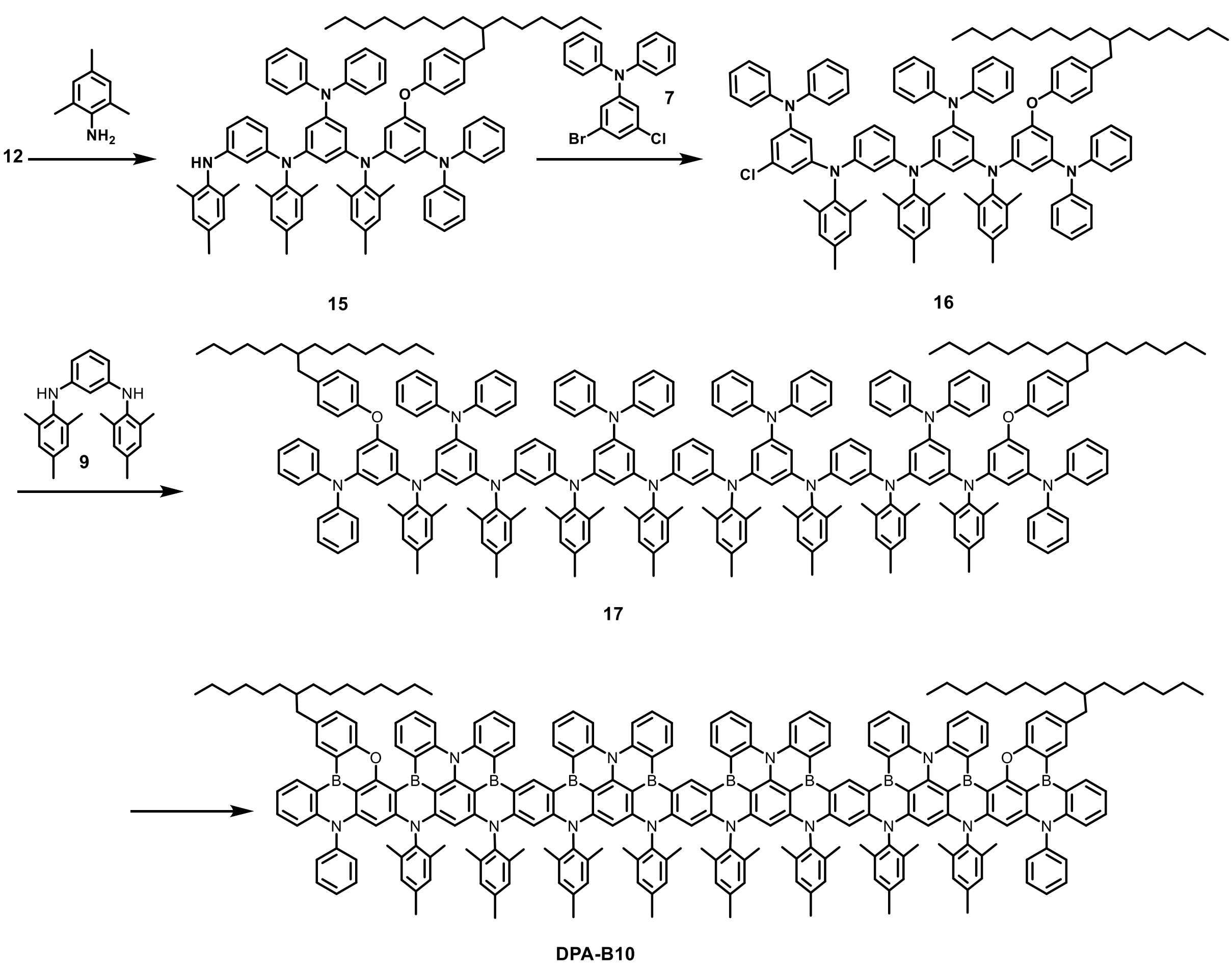

Scheme S3. Synthesis route of **DPA-B10**.

*Synthesis of N^1-(3-(diphenylamino)-5-(4-(2-hexyldecyl)phenoxy)phenyl)-N^1,N^3-dimesityl-N^3-(3-(mesitylamino)phenyl)-N^5,N^5-diphenylbenzene-1,3,5-triamine (**15**)*

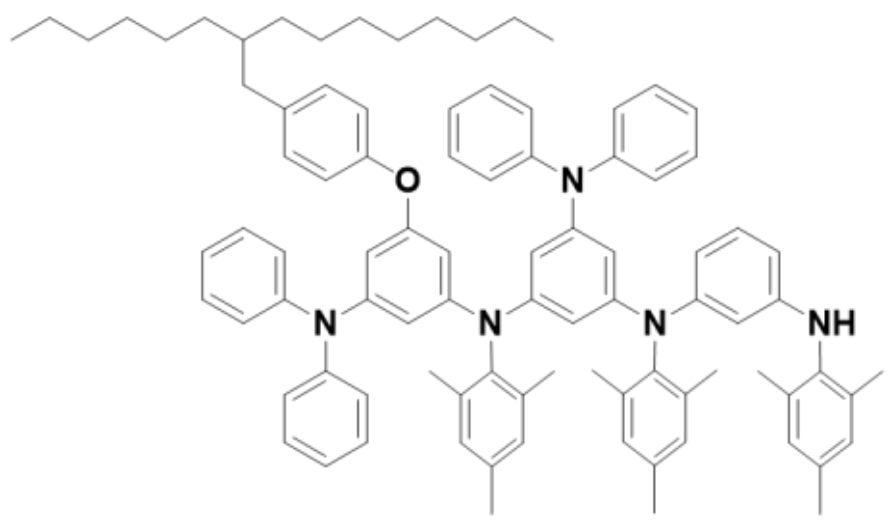

The synthesis procedure of compound **15** is similar to that of compound **11**, employing precursor **12** instead of precursor **8**. Compound **15** (82%) was acquired as white solid. ^{1}H NMR (400 MHz, $CDCl_3$) δ 7.17–7.08 (m, 8H), 7.03–6.93 (m, 10H), 6.92–6.81 (m, 6H), 6.78–6.69 (m, 5H), 6.67 (s, 2H), 6.37–6.28 (m, 3H), 6.28–6.23 (m, 2H), 6.11–6.05 (m, 2H), 6.03 (dd, *J* = 8.0, 2.0 Hz, 1H), 5.69 (dd, *J* = 8.0, 2.0 Hz, 1H), 4.95 (s, 1H), 2.41 (d, *J* = 7.2 Hz, 2H), 2.26 (s, 3H), 2.23 (s, 3H), 2.18 (s, 3H), 2.07 (s, 6H), 1.86 (d, *J* = 11.2 Hz, 12H), 1.33–1.13 (m, 24H), 0.91–0.81 (m, 6H). ^{13}C NMR (125 MHz, $CDCl_3$) δ 157.81, 155.25, 149.20, 148.49, 148.17, 147.50, 147.29, 146.91, 146.54, 146.33, 139.91, 139.70, 137.21, 136.74, 136.23, 136.13, 136.00, 135.87, 135.75, 135.07, 130.03, 129.50, 129.43, 129.06, 128.99, 128.95, 128.84, 124.52, 124.08, 123.89, 123.53, 122.61, 122.08, 117.27, 117.07, 109.96, 109.86, 109.61, 109.34, 107.78, 106.90, 105.21, 104.86, 104.62, 39.67, 39.64, 33.09, 32.70, 31.95, 29.73, 28.79, 26.54, 23.07, 22.72, 20.97, 20.92, 20.90, 18.27, 18.25, 18.17, 18.02, 14.19, 14.16.

*Synthesis of N^1-(3-((3-chloro-5-(diphenylamino)phenyl)(mesityl)amino)phenyl)-N^3-(3-(diphenylamino)-5-(4-(2-hexyldecyl)phenoxy)phenyl)-N^1,N^3-dimesityl-N^5,N^5-diphenylbenzene-1,3,5-triamine (**16**)*

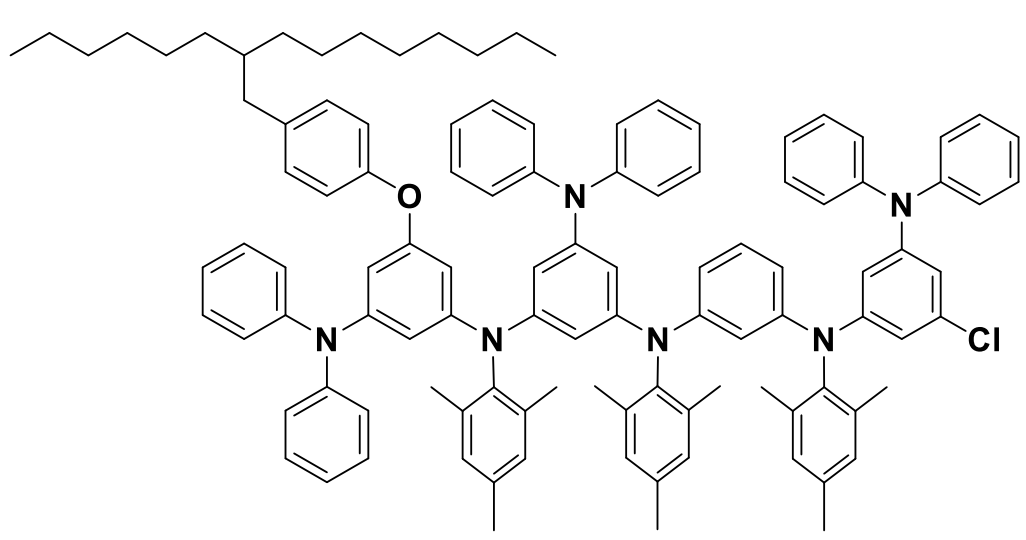

The synthesis procedure of compound **16** is similar to that of compound **8**, employing precursor **15** instead of precursor **6**. Compound **16** (75%) was acquired as white solid. ^{1}H NMR (400

MHz, $CDCl_3$) δ 7.20–7.06 (m, 12H), 7.01–6.90 (m, 16H), 6.89–6.81 (m, 3H), 6.78–6.71 (m, 5H), 6.65 (d, *J* = 11.6 Hz, 4H), 6.47 (d, *J* = 2.0 Hz, 1H), 6.44–6.39 (m, 2H), 6.36 (d, *J* = 2.0 Hz, 1H), 6.30 (d, *J* = 2.0 Hz, 1H), 6.28–6.24 (m, 2H), 6.23–6.19 (m, 2H), 6.15 (d, *J* = 2.0 Hz, 1H), 6.06 (d, *J* = 2.0 Hz, 1H), 5.89 (d, *J* = 2.0 Hz, 1H), 2.41 (d, *J* = 6.8 Hz, 2H), 2.22 (s, 3H), 2.19 (s, 6H), 1.83 (s, 6H), 1.79 (s, 6H), 1.67 (s, 6H), 1.51–1.47 (m, 1H), 1.30–1.13 (m, 24H), 0.90–0.83 (m, 6H). ^{13}C NMR (125 MHz, $CDCl_3$) δ 158.13, 155.10, 149.18, 149.15, 148.43, 147.90, 147.75, 147.40, 147.33, 147.16, 146.81, 146.61, 146.08, 145.51, 139.75, 139.58, 139.45, 137.01, 136.83, 136.72, 136.62, 136.23, 136.08, 135.95, 135.07, 130.04, 129.72, 129.51, 129.41, 129.19, 129.01, 128.88, 124.26, 124.05, 123.47, 123.01, 122.60, 122.11, 117.55, 115.38, 113.03, 112.53, 112.28, 111.01, 109.83, 109.69, 109.48, 107.59, 106.81, 104.65, 39.71, 39.67, 33.06, 31.96, 30.07, 29.73, 29.70, 29.39, 26.57, 26.54, 22.74, 20.99, 20.93, 18.26, 18.20, 18.03, 14.18.

*Synthesis of N^1,N^{1}'-(1,3-phenylene)bis(N^3-(3-((3-(diphenylamino)-5-((3-(diphenylamino)-5-(4-(2-hexyldecyl)phenoxy)phenyl)(mesityl)amino)phenyl)(mesityl)amino)phenyl)-N^1,N^3-dimesityl-N^5,N^5-diphenylbenzene-1,3,5-triamine) (**17**)*

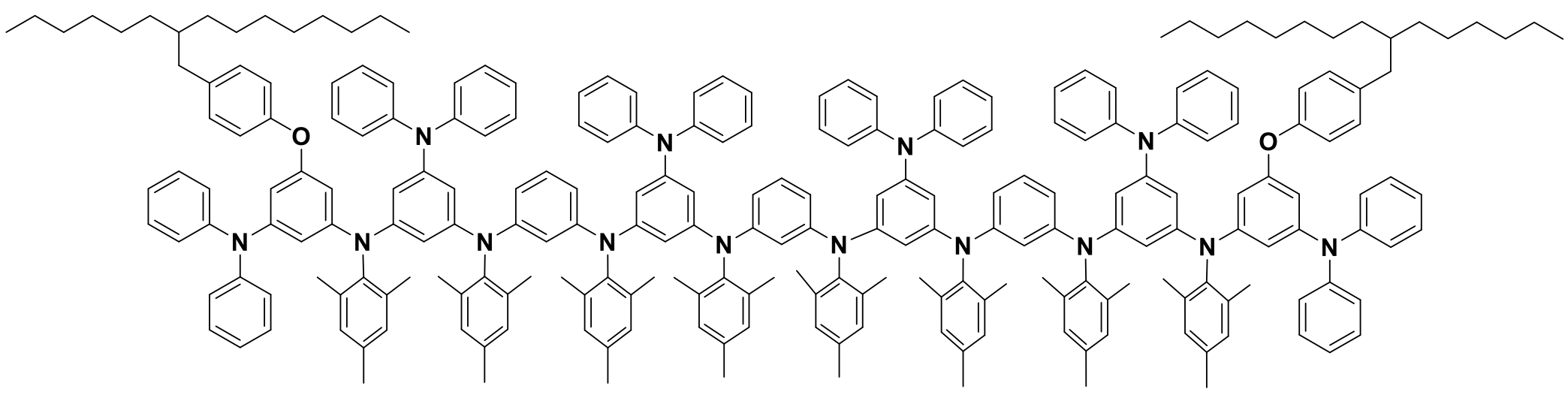

The synthesis procedure of compound **17** is similar to that of compound **10**, employing precursor **16** instead of precursor **8**. Precursor **17** (56%) was acquired as white solid. ^1H NMR (400 MHz, $CDCl_3$) δ 7.15–7.00 (m, 26H), 6.98–6.85 (m, 32H), 6.84–6.76 (m, 8H), 6.73 (d, *J* = 8.8 Hz, 4H), 6.67–6.62 (m, 6H), 6.56 (d, *J* = 4.8 Hz, 12H), 6.32 (d, *J* = 2.0 Hz, 2H), 6.27–6.10 (m, 18H), 6.04 (d, *J* = 2.0 Hz, 2H), 5.80 (d, *J* = 2.0 Hz, 2H), 5.74 (s, 2H), 2.28–2.13 (m, 24H), 1.81 (s, 12H), 1.66–1.56 (m, 36H), 1.51–1.46 (m, 2H), 1.33–1.13 (m, 48H), 0.91–0.82 (m, 12H). ^{13}C NMR (101 MHz, $CDCl_3$) δ 158.09, 155.13, 149.09, 148.25, 148.03, 147.97, 147.44, 147.40, 147.33, 146.99, 146.93, 146.44, 146.12, 145.98, 145.86, 140.00, 139.76, 136.86, 136.84, 136.83, 136.75, 136.72, 136.15, 135.90, 135.82, 135.74, 135.73, 130.01, 129.52, 129.48,

129.27, 129.24, 129.14, 128.99, 128.89, 128.87, 128.81, 124.45, 123.99, 123.47, 123.40, 123.33, 122.55, 122.03, 121.94, 117.53, 111.45, 111.41, 111.34, 111.32, 111.27, 109.79, 109.72, 109.64, 109.47, 109.15, 107.53, 107.31, 106.71, 104.58, 77.38, 77.06, 76.74, 39.69, 39.66, 33.05, 31.96, 30.07, 29.73, 29.70, 29.38, 26.57, 26.53, 22.73, 20.96, 20.94, 20.92, 18.21, 18.07, 18.05, 17.99, 14.18.

*Synthesis of **DPA-B10***

The synthesis procedure of **DPA-B10** is similar to that of **DPA-B6**, employing precursor **17** instead of precursor **10**. **DPA-B10** (12%) was acquired as pale yellow solid. ^{1}H NMR (500 MHz, $CDCl_3$) δ 10.63–10.46 (m, 3H), 9.48–9.39 (m, 2H), 9.15–9.07 (m, 3H), 9.02 (d, J = 7.5 Hz, 2H), 8.94 (d, J = 7.5 Hz, 2H), 8.62 (s, 2H), 8.56–8.46 (m, 4H), 8.44–8.29 (m, 3H), 7.78–7.51 (m, 18H), 7.49–7.34 (m, 10H), 7.32–7.30 (m, 2H), 7.19–7.08 (m, 4H), 6.79 (d, J = 9.0 Hz, 2H), 6.68–6.51 (m, 16H), 5.94 (d, J = 4.5 Hz, 3H), 5.40 (s, 2H), 5.32 (s, 2H), 5.26 (d, J = 5.5 Hz, 4H), 2.82 (d, J = 6.5 Hz, 4H), 2.36–2.20 (m, 24H), 1.87–1.78 (m, 2H), 1.58–1.52 (m, 12H), 1.49–1.28 (m, 84H), 0.94–0.84 (m, 12H).

Electrochemical Characterization: Cyclic voltammograms (CVs) were obtained in dichloromethane at room temperature with a CHI600 electrochemical workstation at 25 °C and a scan speed of 50 mV s^{-1}. The electrochemical oxidation potentials were collected by cyclic voltammetry measurements via a CHI660 electrochemical workstation (Chenhua, China) and ferroceniumferrocene (Fc/Fc^+) was used as the internal reference, tetrabutylammonium hexafluorophosphate (0.1 M) was used as the supporting electrolyte. A platinum plate electrode was utilized as the working electrode, a platinum wire was utilized as the counter electrode and Ag/AgCl as reference electrode. The reduction potentials were calculated from E_{ox} and E_g, the optical bandgaps (E_gs) were estimated from the onset of the absorption spectra.

High-throughput Screening: To construct the comprehensive molecular library evaluated in this study, we implemented a combinatorial fragment-assembly algorithm. The workflow initializes with a predefined repository of core structural building blocks, specifically classified into fragments A, B1, B2, C1, C2, and C3. The algorithm systematically enumerates possible

topological linkages by permuting these primary fragments. To control the size and complexity of the generated structures, the algorithm is constrained by a targeted degree of polymerization, denoted as n. By iteratively executing the assembly process for lengths of n=2,3,4, the algorithm generates a highly diverse set of n-mers. This systematic exploration of sequence space ensures an unbiased coverage of the available chemical combinations. Following the automated generation, the resulting oligomers are subjected to property evaluation. Initial 3D conformers were generated and refined using an MM force-field workflow implemented in RDKit. For each molecule, all enumerated conformers were subjected to energy minimizations using UFF. Conformers were then sorted by energy and filtered to remove near-duplicates. The remaining unique, lowest-energy conformer was kept as the initial conformer set for subsequent calculations.

Quantum Chemical Calculations

Theoretical chemical calculations were performed by the Gaussian 16 program package[4]. All generated structures were further optimized using Density Functional Theory (DFT) at the PBE0/def2-SVP level[5], incorporating Grimme's D3 dispersion correction with Becke-Johnson damping (GD3BJ). Subsequently, excited-state geometries were optimized using Time-Dependent Density Functional Theory (TD-DFT) at the same level of theory. Based on the optimized stationary points of both the ground and excited states, the reorganization energies were calculated utilizing the four-point method. Finally, based on the HOMO and LUMO of the ground state, the differences in Mayer bond orders between the ground and excited states for every bond within each molecule were evaluated using the Multiwfn program[6]. Molecular structure models and isosurface plots were rendered using Multiwfn and VMD[7,8].

Photophysical Measurements

Absorption spectra were measured on Shimadzu UV-2700 spectrophotometer. Steady-state emission and low-temperature emission spectra were recorded on a Hitachi F-7100 fluorescence spectrophotometer at room temperature. Transient PL delay curves were obtained using Fluotime 300 (PicoQuant) with a picosecond pulsed UV-LASTER (LASTER375) as the excitation source. Photoluminescence quantum yields (Φ_{PL}s) were measured by a Hamamatsu

ultraviolet-near-infrared absolute PL quantum yield spectrometer (C13534, Hamamatsu Photonics) equipped with a calibrated integrating sphere, and the integrating sphere was purged with dry argon to maintain an inert atmosphere.

Analysis of Rate Constants: The rate constants of radiative decay (k_r) and nonradiative decay (k_{nr}) from S_1 to S_0 states, intersystem crossing (k_{ISC}) and reverse intersystem crossing (k_{RISC}) were calculated from the following six equations[9,10]

$$k_\mathrm{p} = 1/\tau_\mathrm{p} \quad \text{Eq.(1)}$$

$$k_\mathrm{d} = 1/\tau_\mathrm{d} \quad \text{Eq.(2)}$$

$$k_\mathrm{r} = \Phi_\mathrm{p} k_\mathrm{p} + \Phi_\mathrm{d} k_\mathrm{d} \approx \Phi_\mathrm{p} k_\mathrm{p} \quad \text{Eq.(3)}$$

$$k_\mathrm{nr} = \frac{1-\Phi_\mathrm{PL}}{\Phi_\mathrm{PL}} k_\mathrm{r} \quad \text{Eq.(4)}$$

$$k_\mathrm{ISC} = k_\mathrm{p} - k_\mathrm{r} - k_\mathrm{nr} \quad \text{Eq.(5)}$$

$$k_\mathrm{RISC} = (k_\mathrm{p} k_\mathrm{d} \Phi_\mathrm{d})/(k_\mathrm{ISC} \Phi_\mathrm{p}) \quad \text{Eq.(6)}$$

Where τ_p and τ_d represent the prompt and decay fluorescence lifetime, which determined from transient photoluminescence (PL) spectra. The k_p and k_d represent the decay rate constants for prompt and delayed fluorescence, respectively. Φ_p and Φ_d indicate prompt and delayed fluorescence components and can be distinguished from the total Φ_PL by comparing the integrated intensities of prompt and delayed components in the transient PL spectra.

Determination of Emitting Dipole Orientation

The emitting dipole orientation of an emitting film was measured by angle-resolved and polarization-resolved PL measurements in a Hamamatsu's established molecular orientation measurement system (C13472-01, Hamamatsu Photonics). The samples were attached to a fused silica half-cylinder prism by index matching liquid. The excitation of the samples was performed with the 360-nm line of the continuous-wave laser with a fixed excitation angle of 45°. The angle-dependent *p*-polarized emission intensity at the peak wavelength of the PL spectrum of the emitting layer was detected. The horizontal dipole orientation ($\Theta_{//}$) was then determined by least-square fitting of the measured angle-dependent *p*-polarized emission

intensity with calculated results.

Device Fabrication and Measurement

SP-OLEDs were fabricated with a configuration of ITO/PEDOT:PSS, 35 nm)/PVK (15 nm)/EML (45 nm)/PO-T2T (10 nm)/ANT-BIZ (50 nm)/Liq (2 nm)/Al (100 nm). ITO-coated glass substrates were ultrasonicated sequentially in acetone and ethanol, followed by the treatment in a UV-ozone oven for 20 min. Afterwards, PEDOT:PSS was spin-coated onto the ITO substrates at ambient temperature at a speed of 4000 rpm for 30 s (~35 nm). The PEDOT:PSS coated ITO substrates were baked at 120 °C for 10 min and then transferred to a glove box with N_2 atmosphere. Then, a 15 nm PVK layer spin-coated from chlorobenzene solution. The emissive layer (using chlorobenzene as the solvent, 10 mg/mL) was spin-coated onto the PVK layer at a speed of 3000 rpm for 30 s (~45 nm), followed by an annealing process (120 ºC for 15 min). Then, the exciton blocking layer (PO-T2T, 10 nm), electron-transporting layer (ANT-BIZ, 50 nm), electron injecting layer (Liq, 2 nm) and cathode (aluminum, 100 nm) were consecutively evaporated in a vacuum chamber under 2×10^{-6} mbar. The devices were tested in ambient environment without encapsulation. The current density-voltage-luminance (*J*-V-*L*), *L*-EQE curves, and electroluminescence spectra were measured using a Keithley 2400 source meter coupled with an absolute EQE measurement system (C9920-12, Hamamatsu Photonics, Japan).

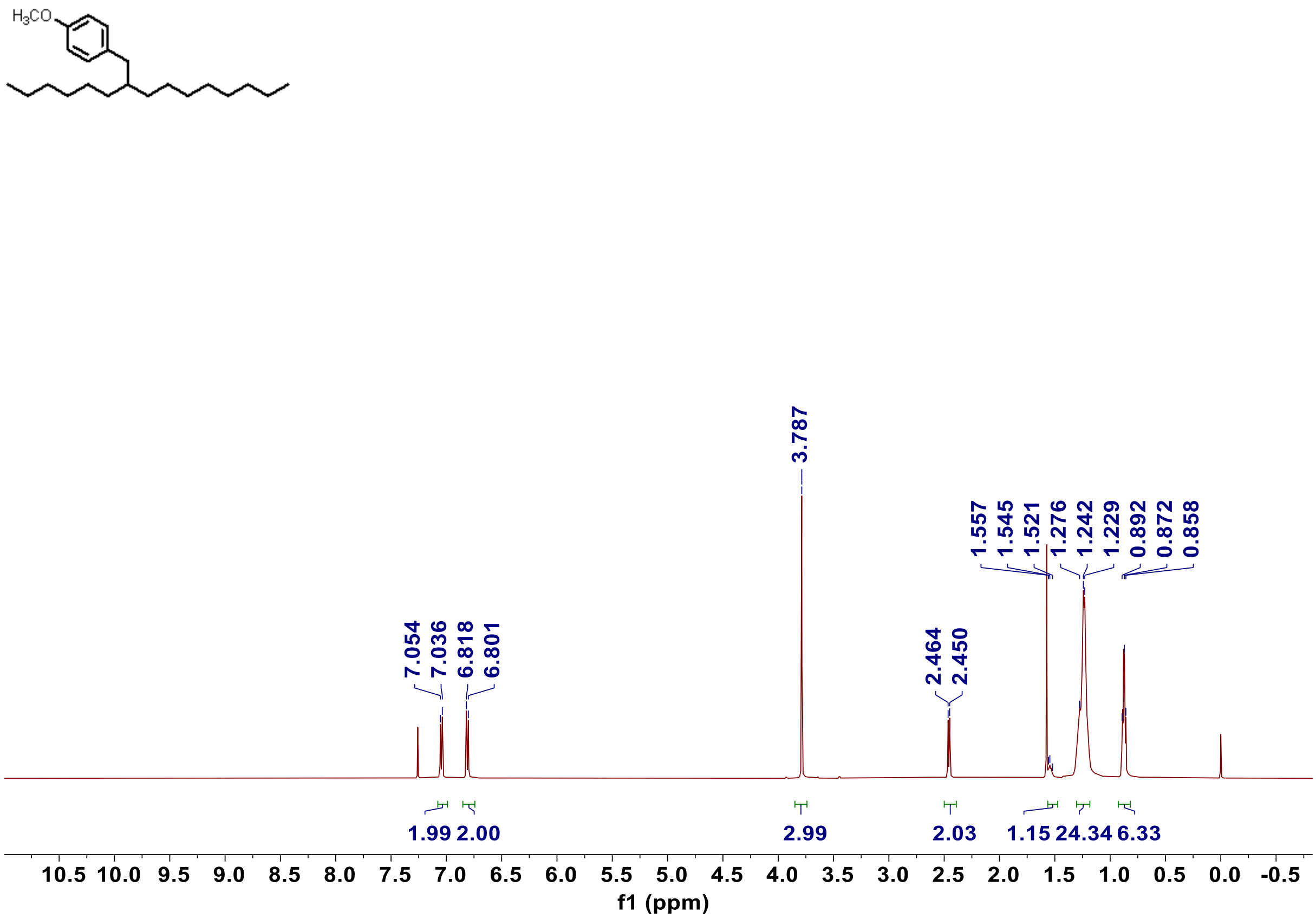

Figure S1. ^{1}H NMR spectrum of compound **2** in $CDCl_3$.

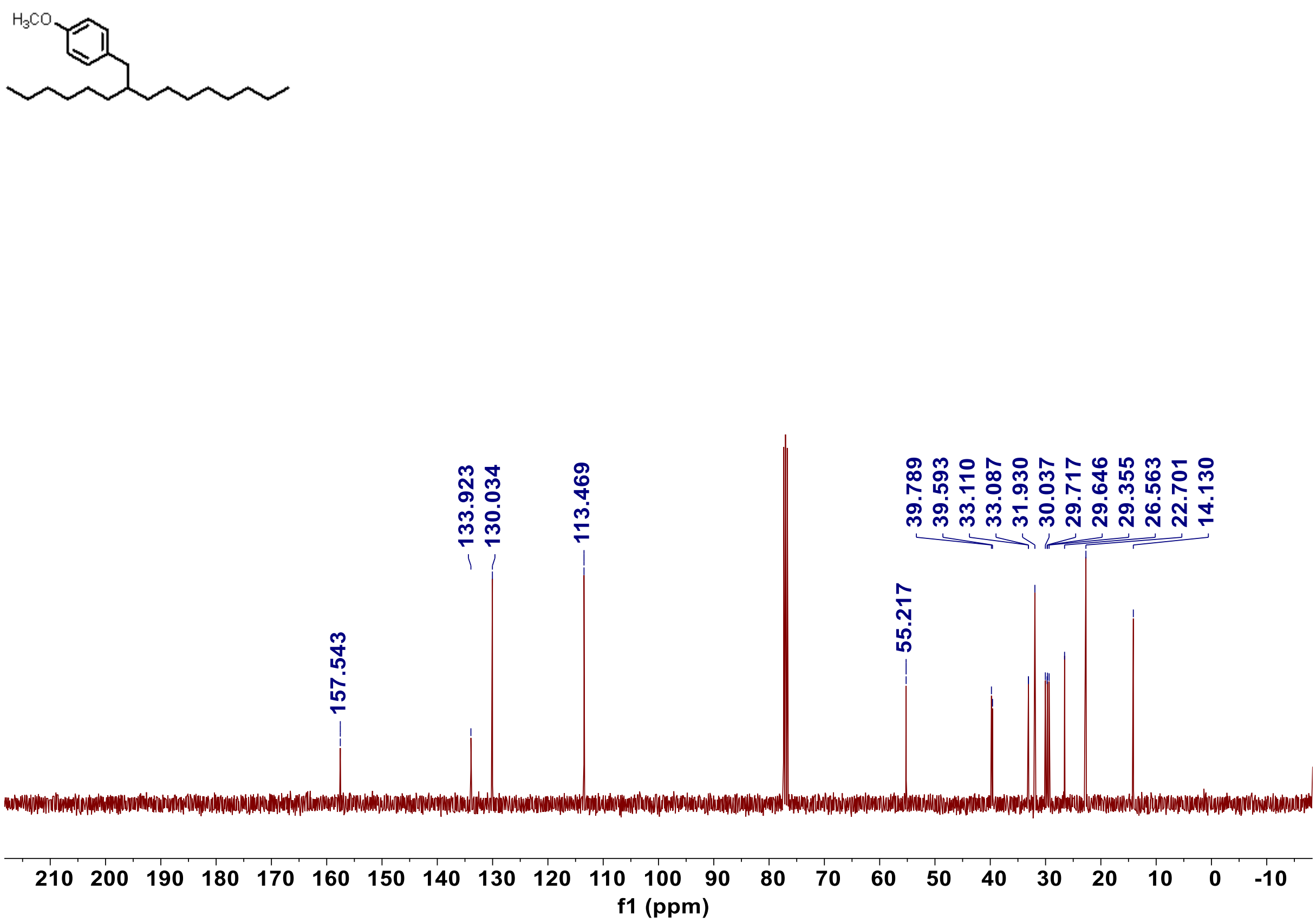

Figure S2. ^{13}C NMR spectrum of compound **2** in $CDCl_3$.

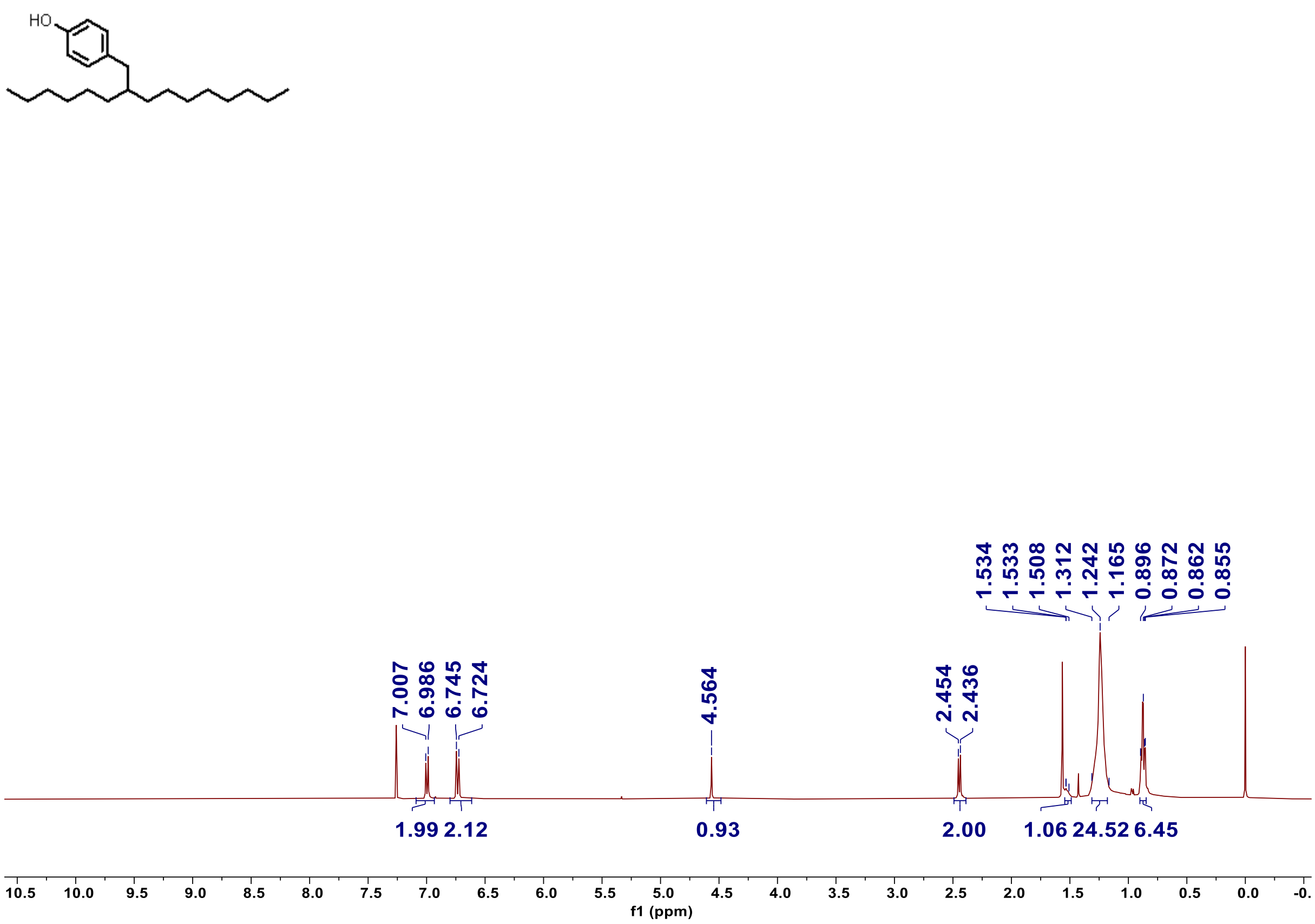

Figure S3. ^{1}H NMR spectrum of compound **3** in $CDCl_3$.

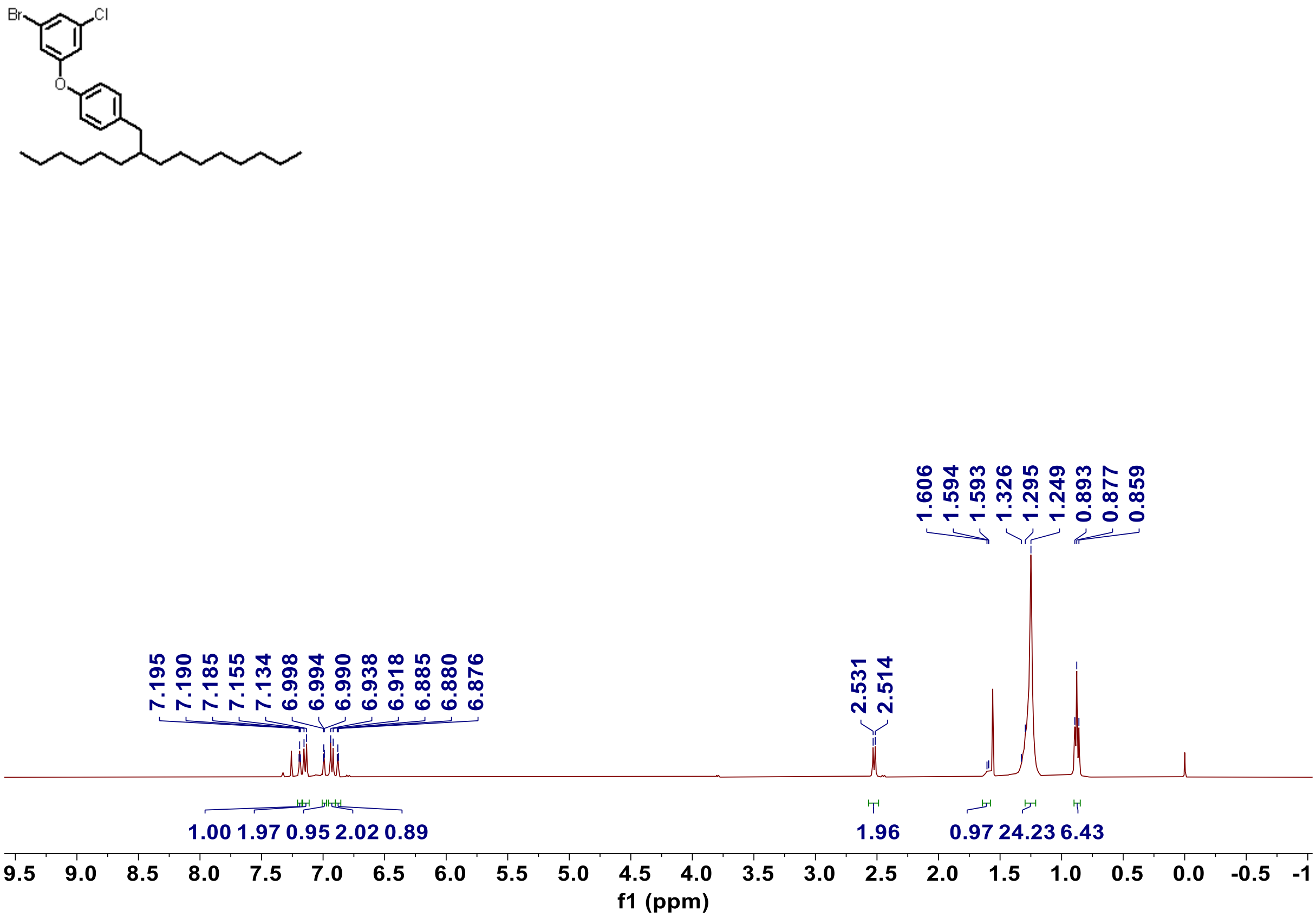

Figure S4. ^{1}H NMR spectrum of compound **4** in $CDCl_3$.

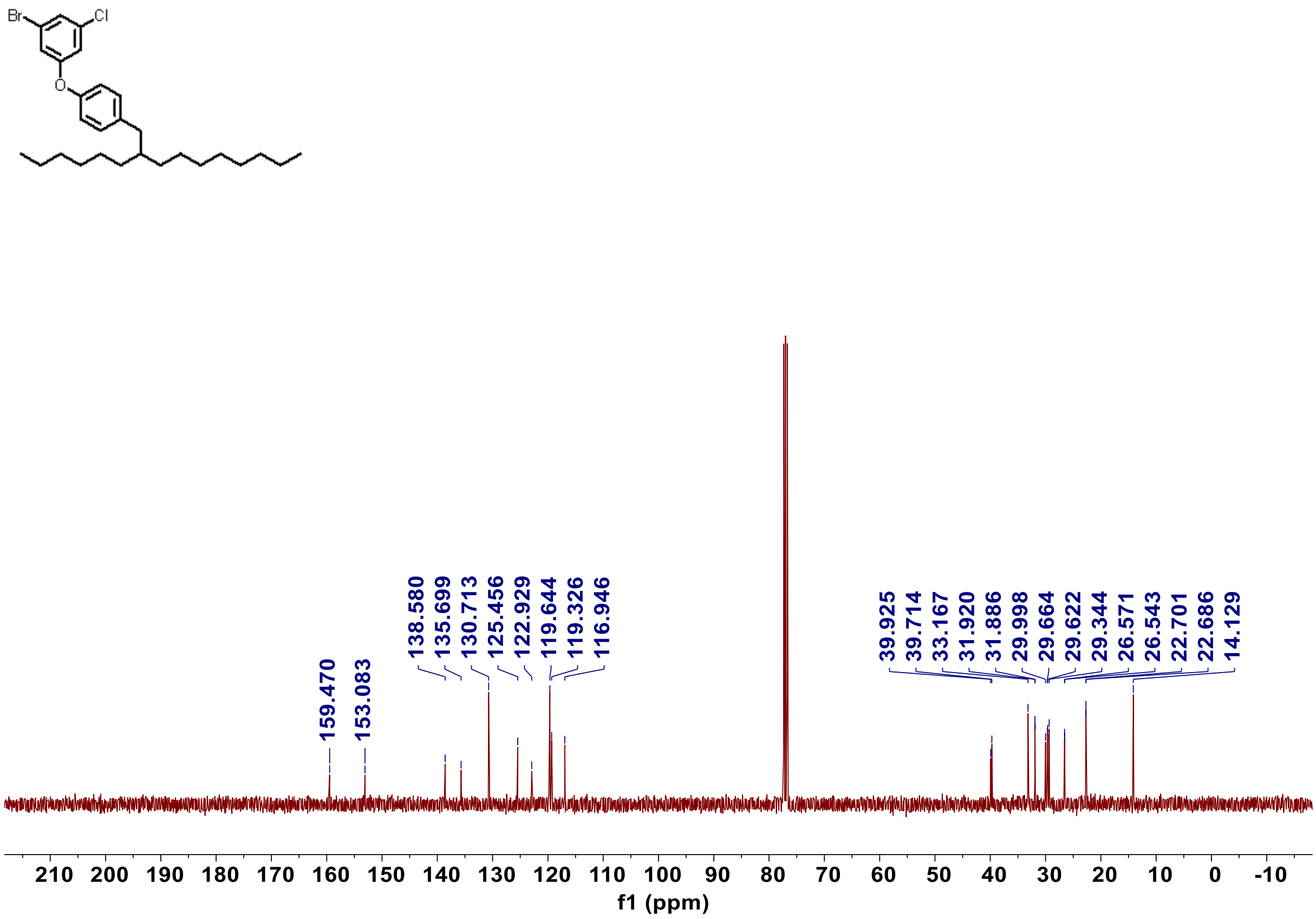

Figure S5. ^{13}C NMR spectrum of compound **4** in $CDCl_3$.

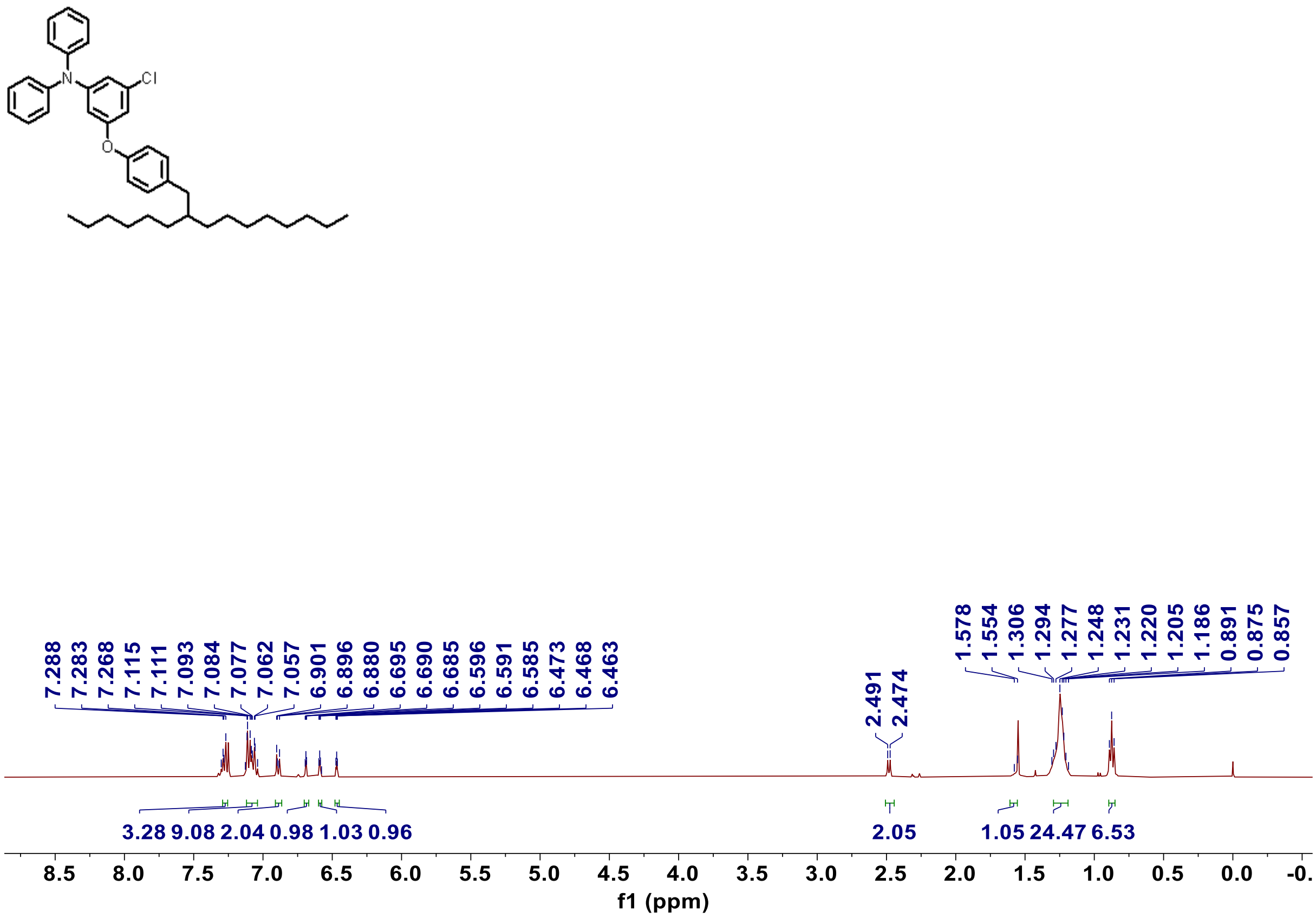

Figure S6. ^{1}H NMR spectrum of compound **5** in $CDCl_3$.

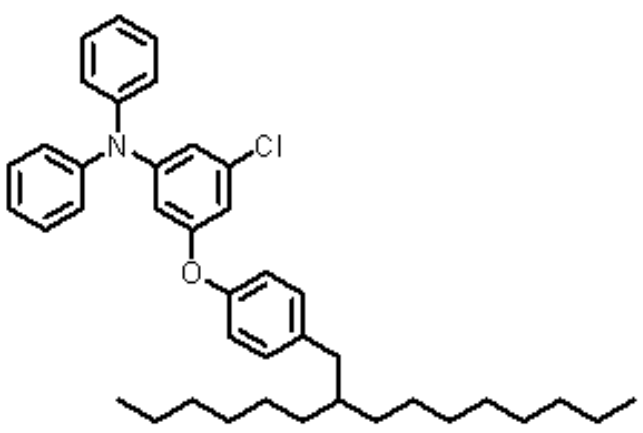

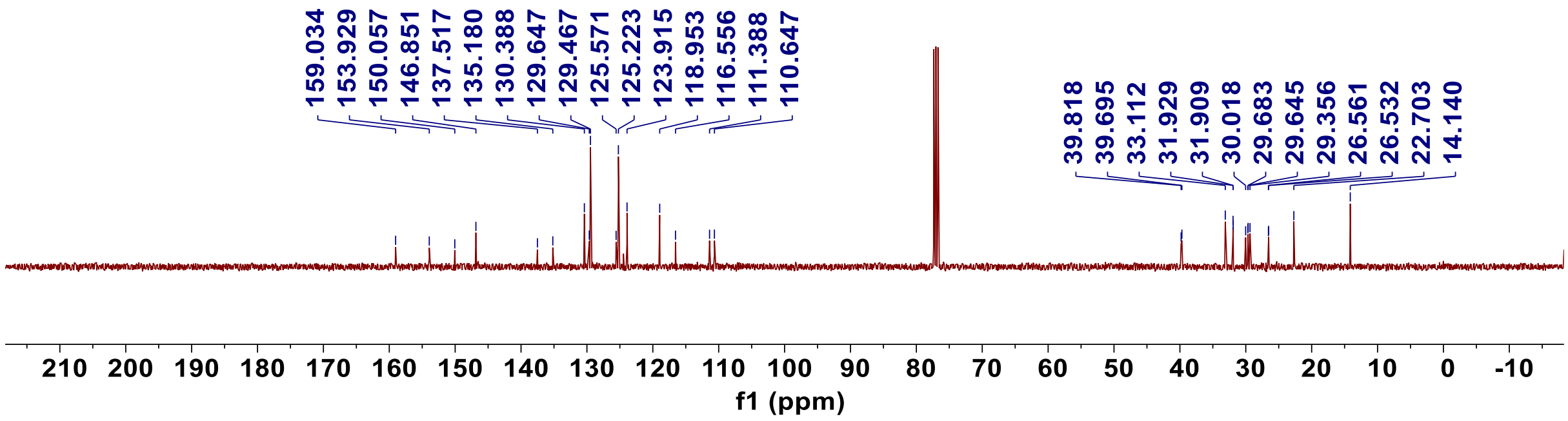

Figure S7. ^{13}C NMR spectrum of compound **5** in $CDCl_3$.

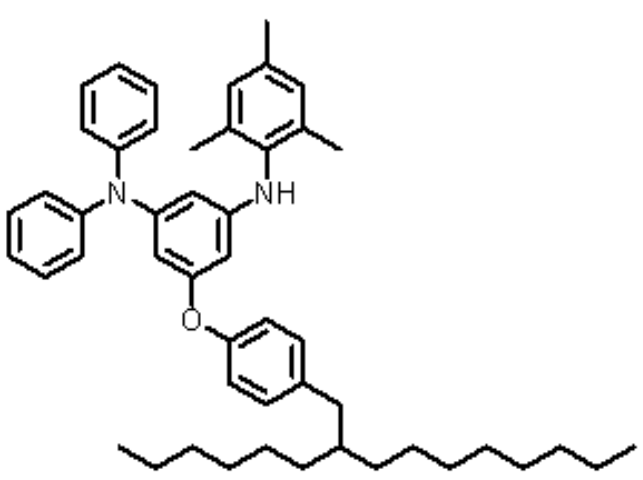

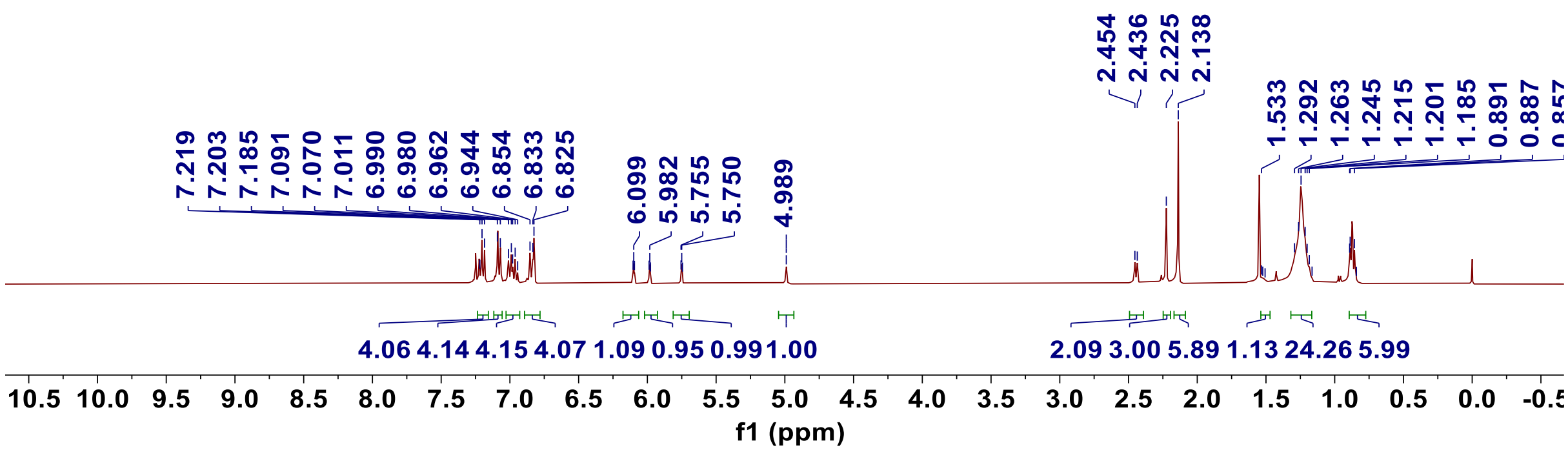

Figure S8. ^{1}H NMR spectrum of compound **6** in $CDCl_3$.

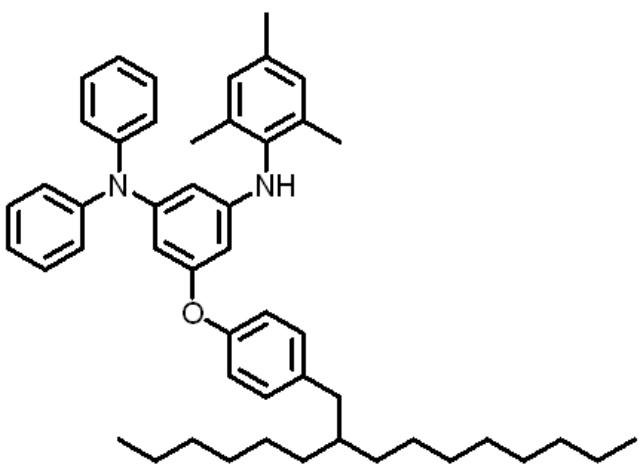

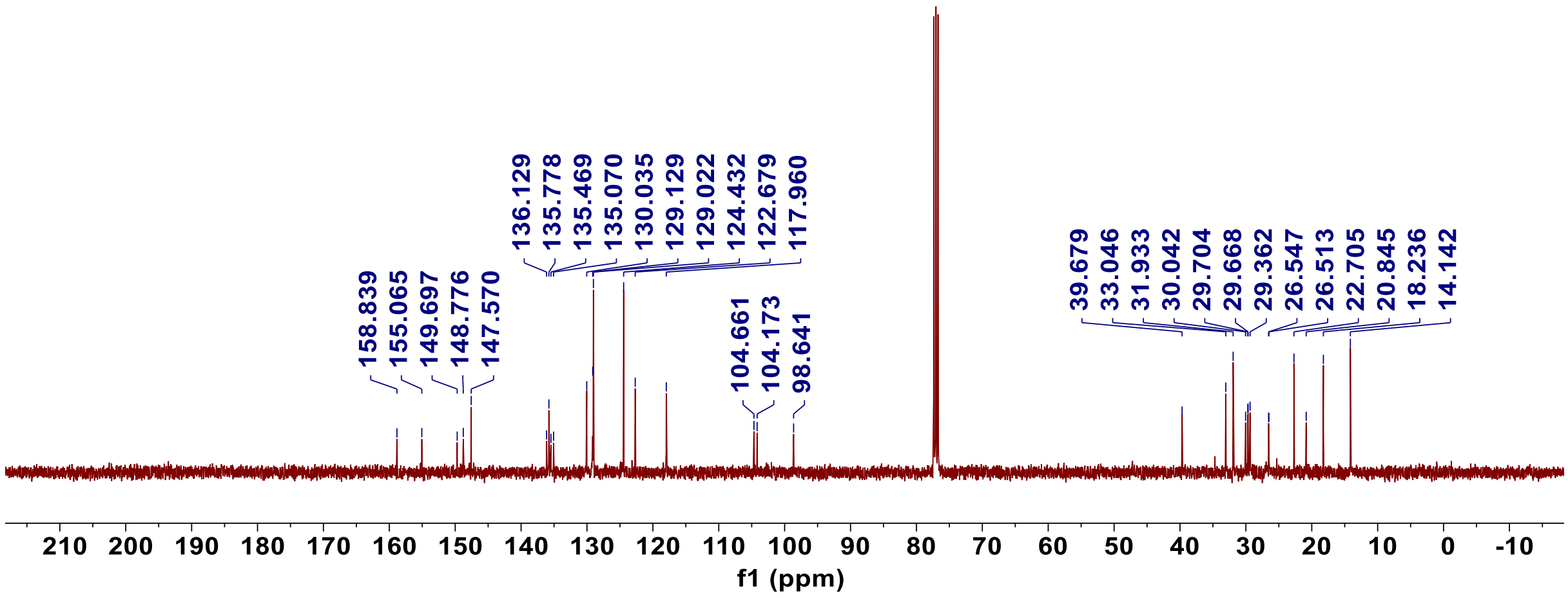

Figure S9. ^{13}C NMR spectrum of compound **6** in $CDCl_3$.

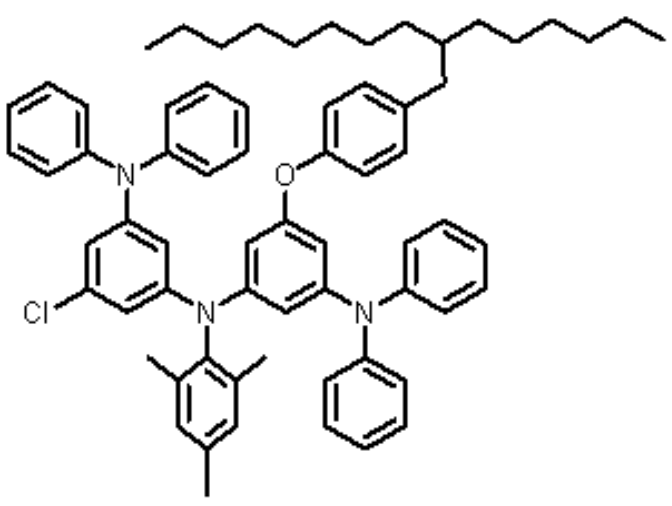

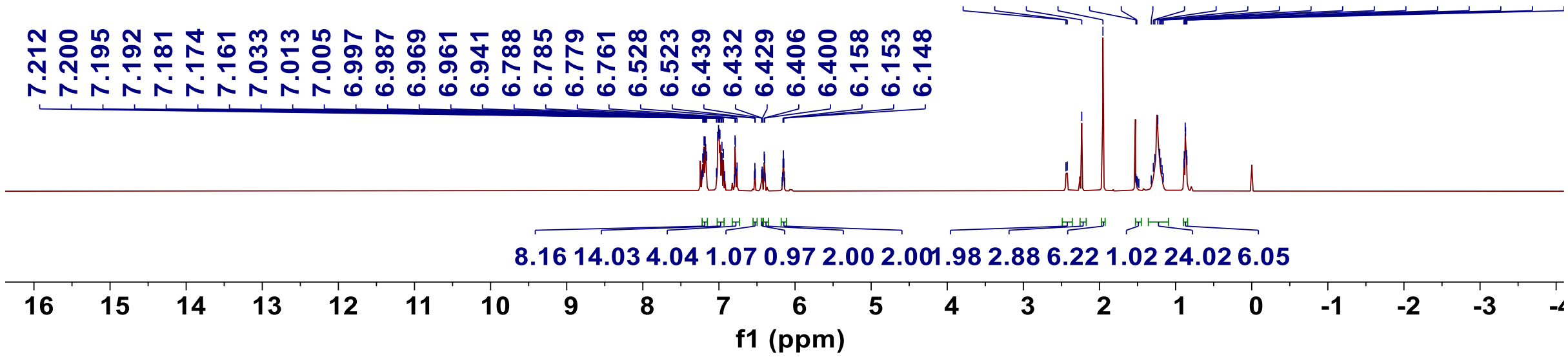

Figure S10. ^{1}H NMR spectrum of compound **7** in $CDCl_3$.

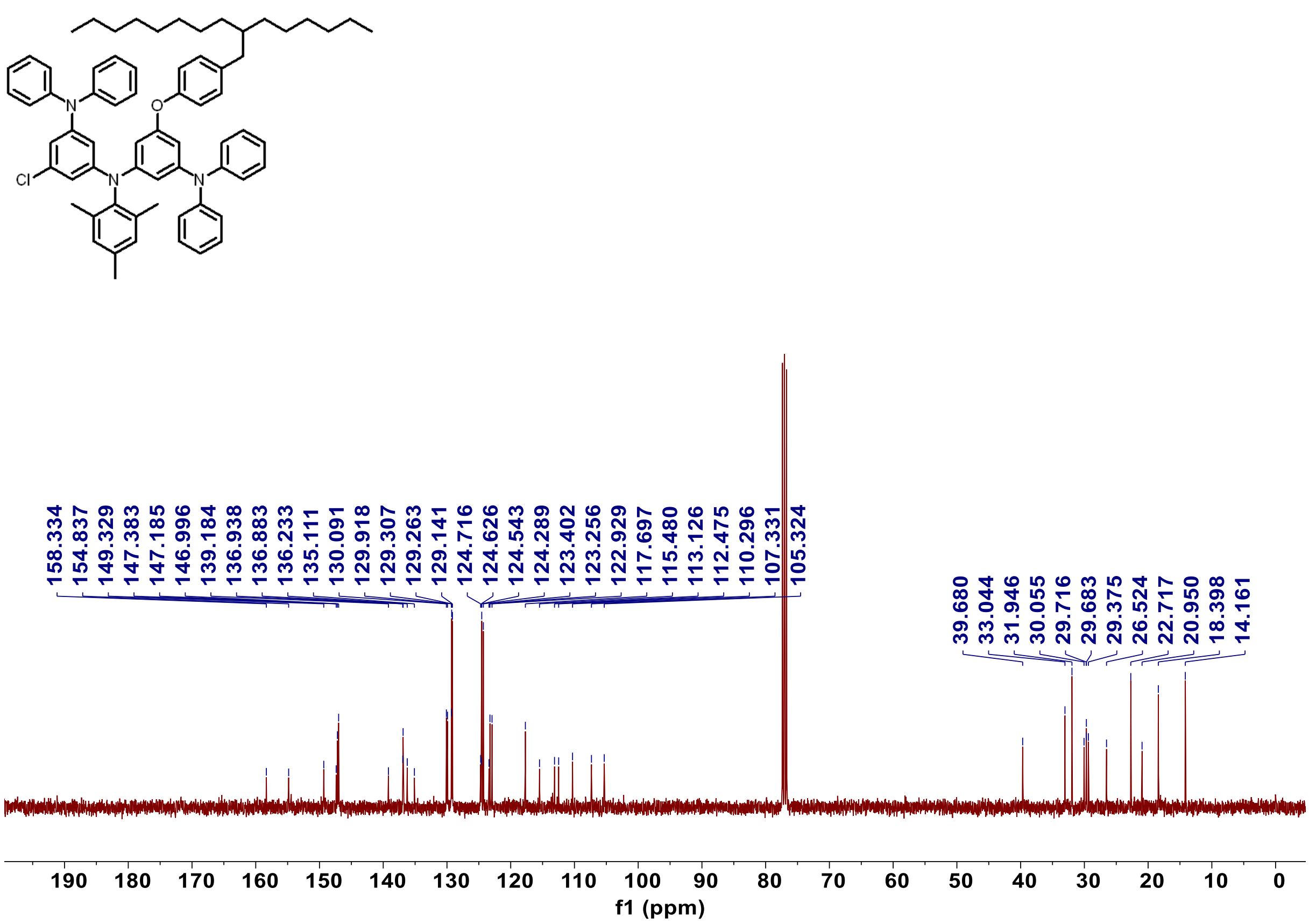

Figure S11. ^{13}C NMR spectrum of compound **7** in $CDCl_3$.

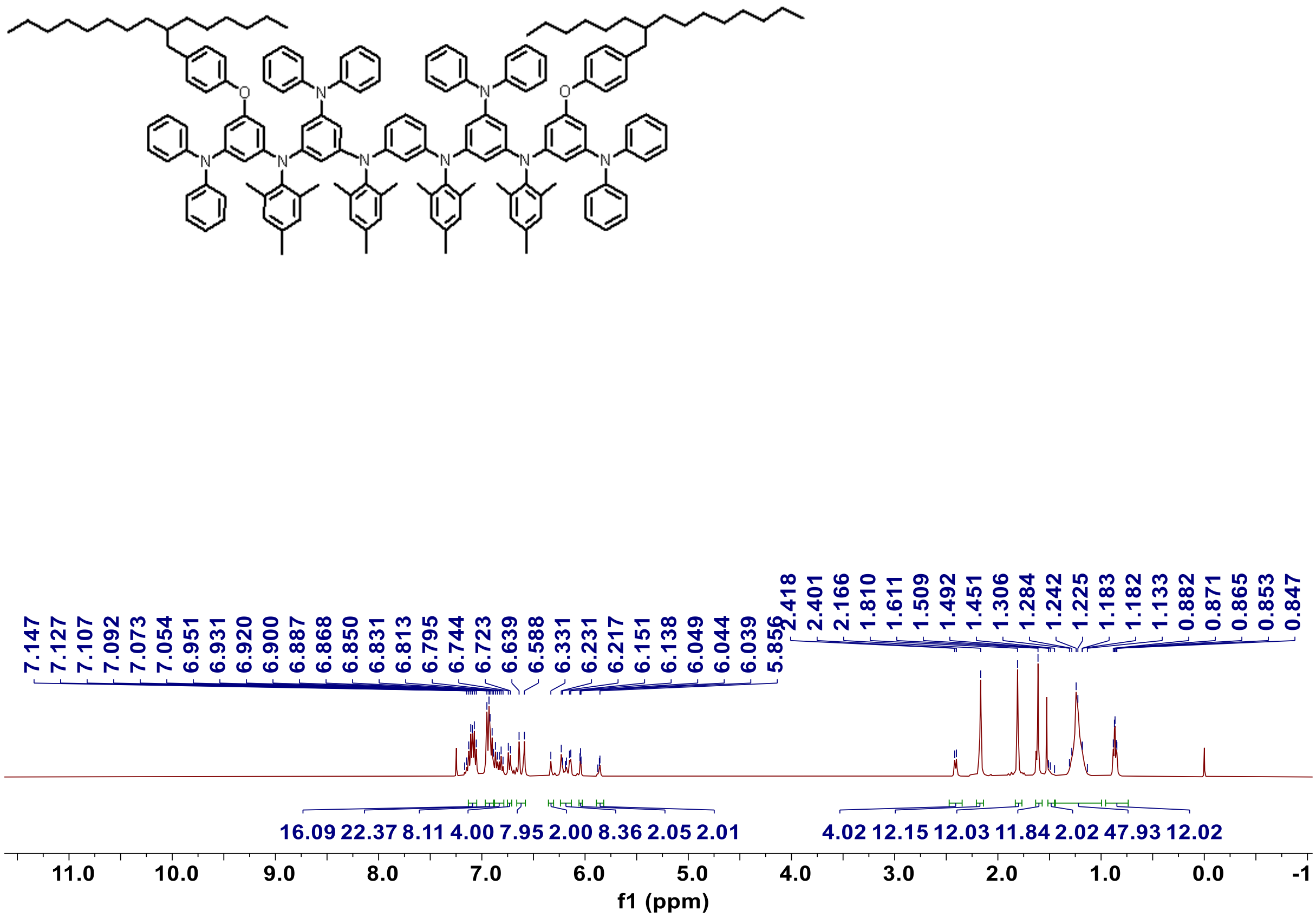

Figure S12. ^{1}H NMR spectrum of compound **10** in $CDCl_3$.

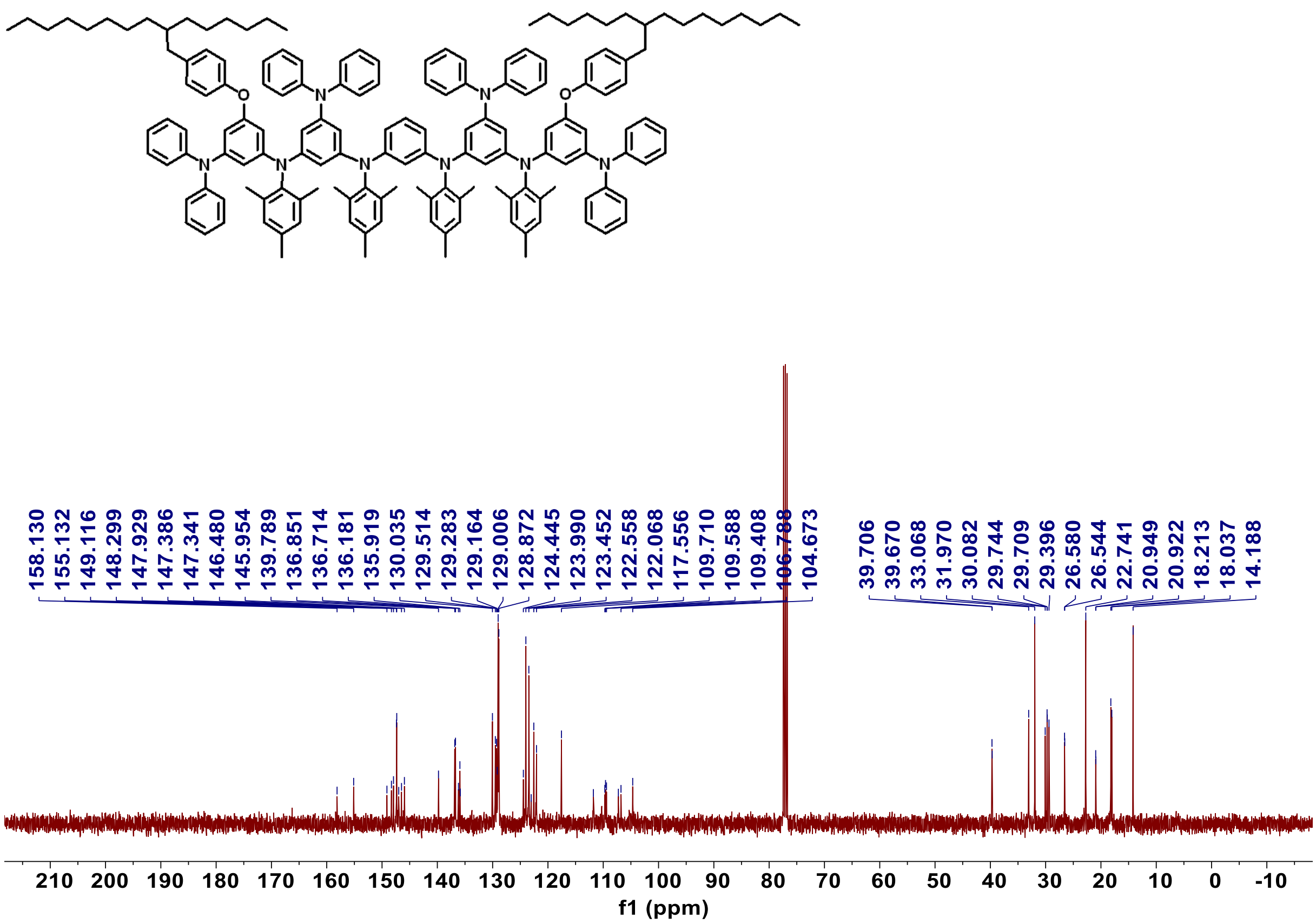

Figure S13. ^{13}C NMR spectrum of compound **10** in $CDCl_3$.

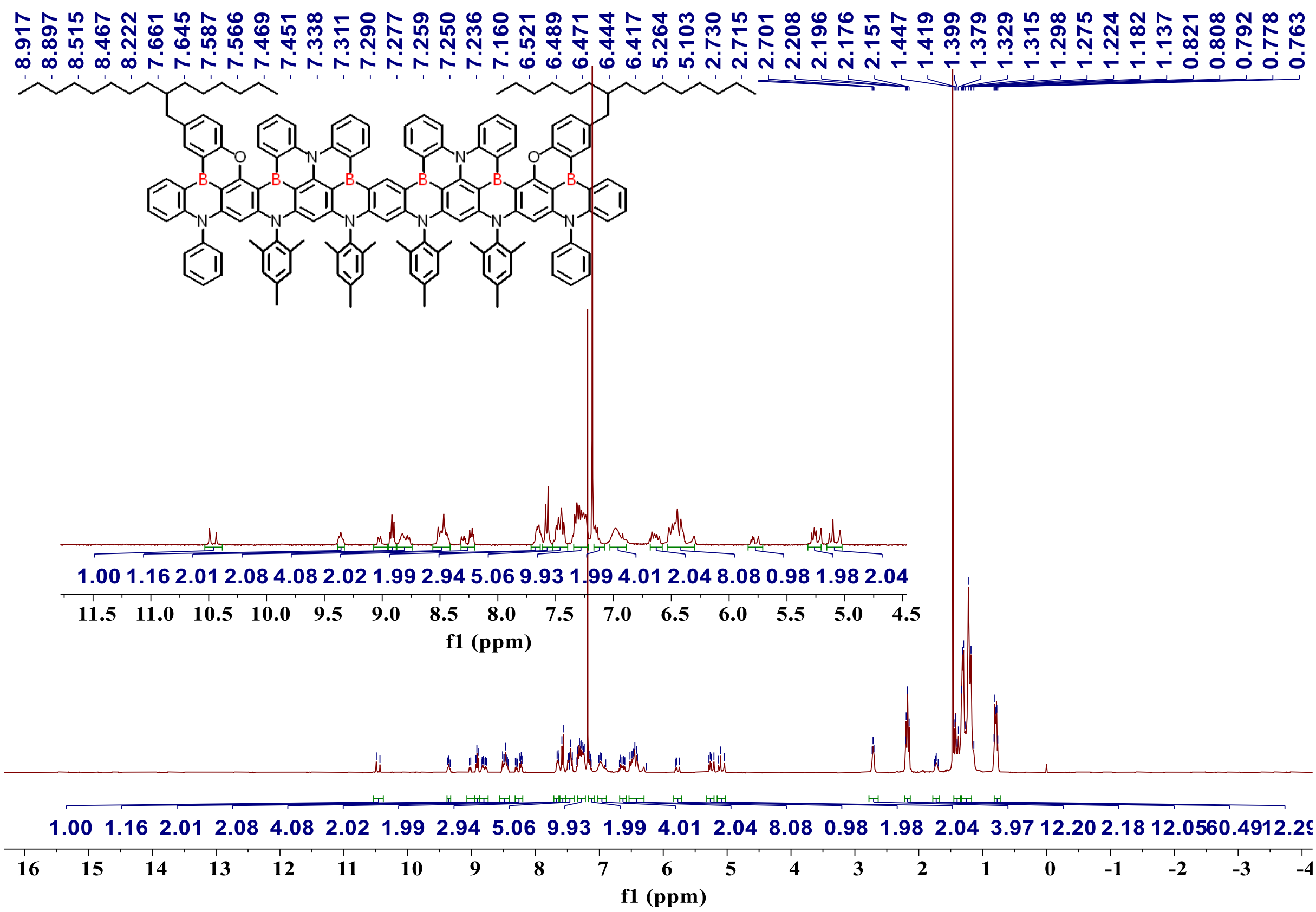

Figure S14. ^{1}H NMR spectrum of **DPA-B6** in $CDCl_3$.

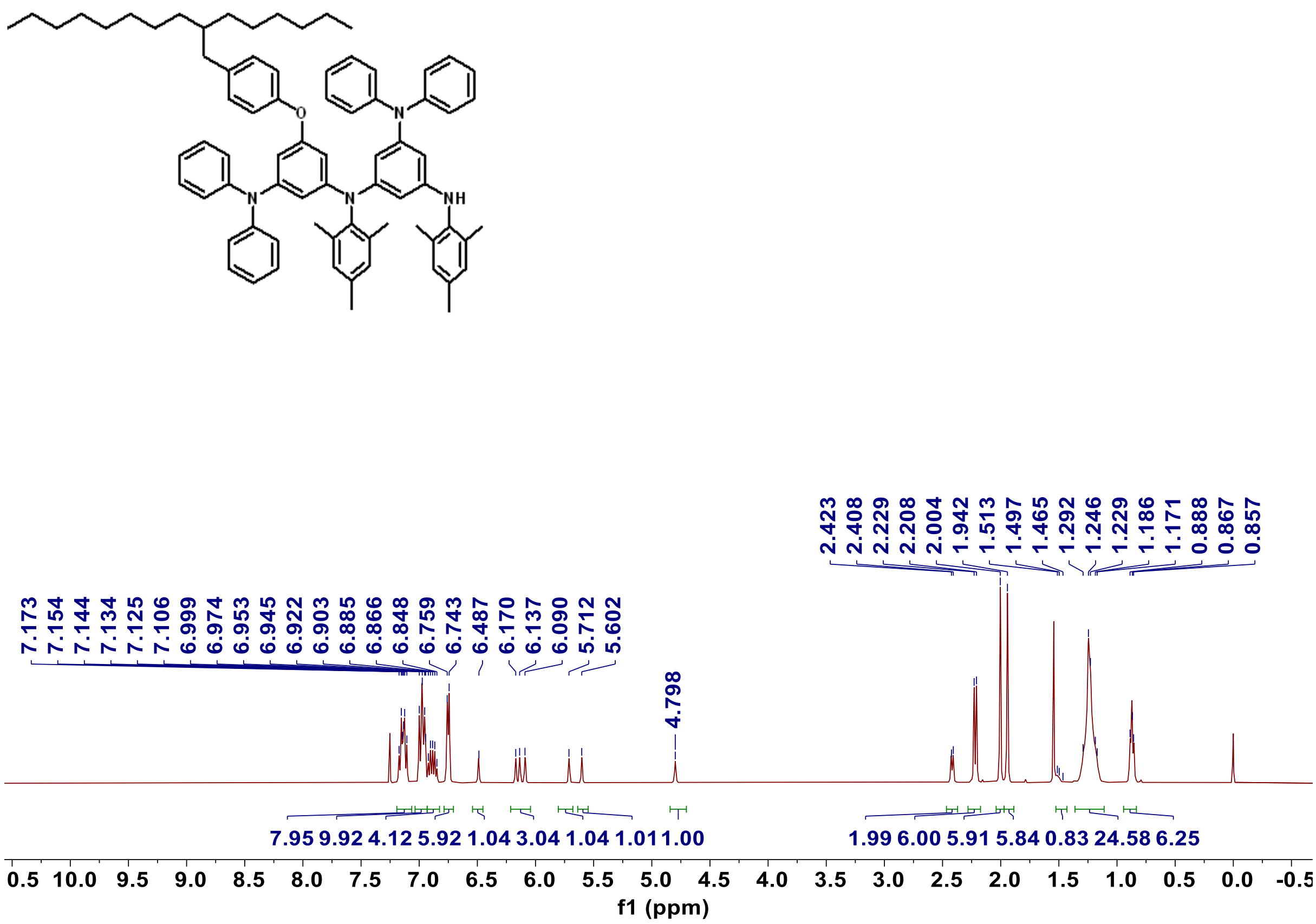

Figure S15. ^{1}H NMR spectrum of compound **11** in $CDCl_3$.

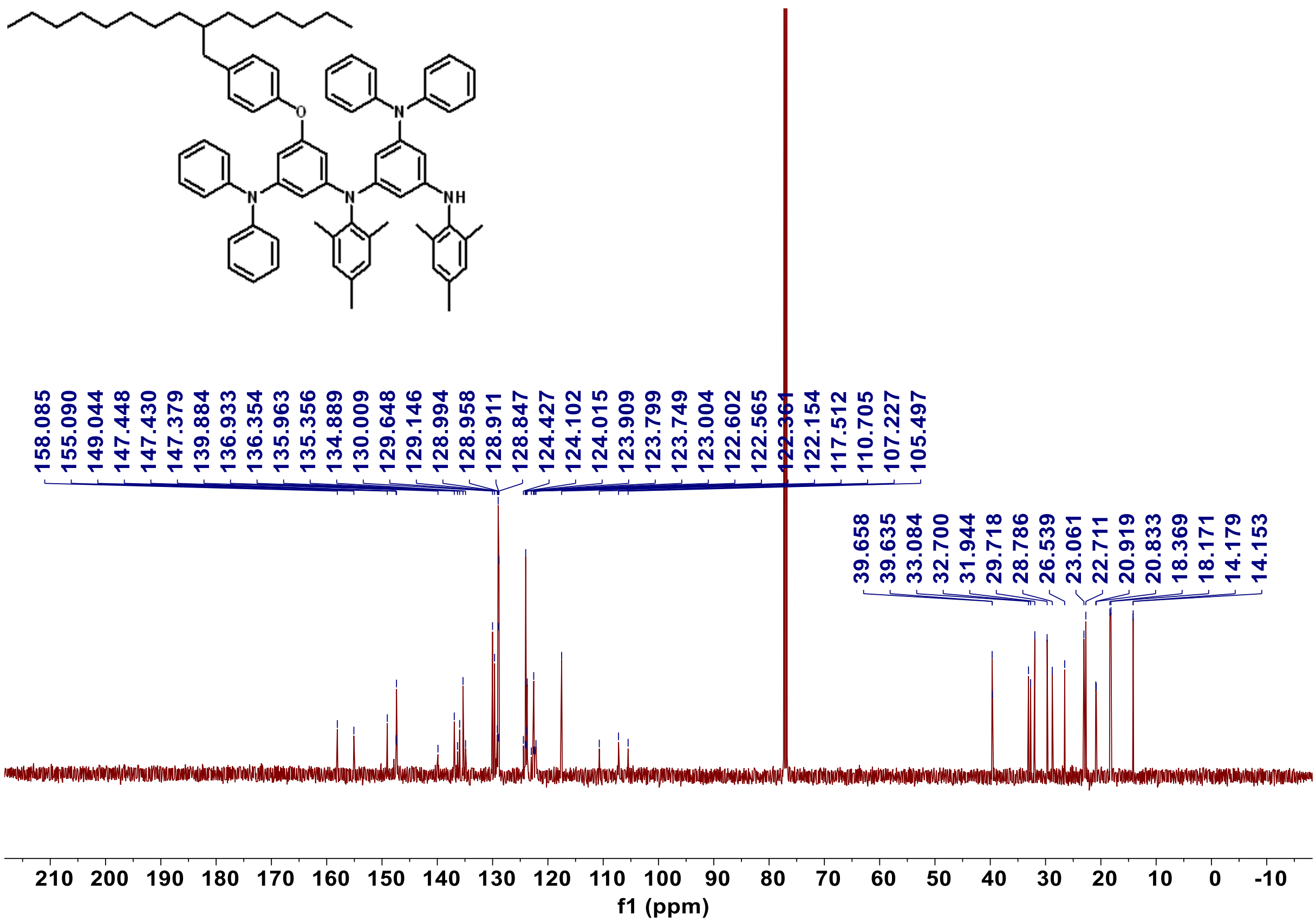

Figure S16. ^{13}C NMR spectrum of compound **11** in $CDCl_3$.

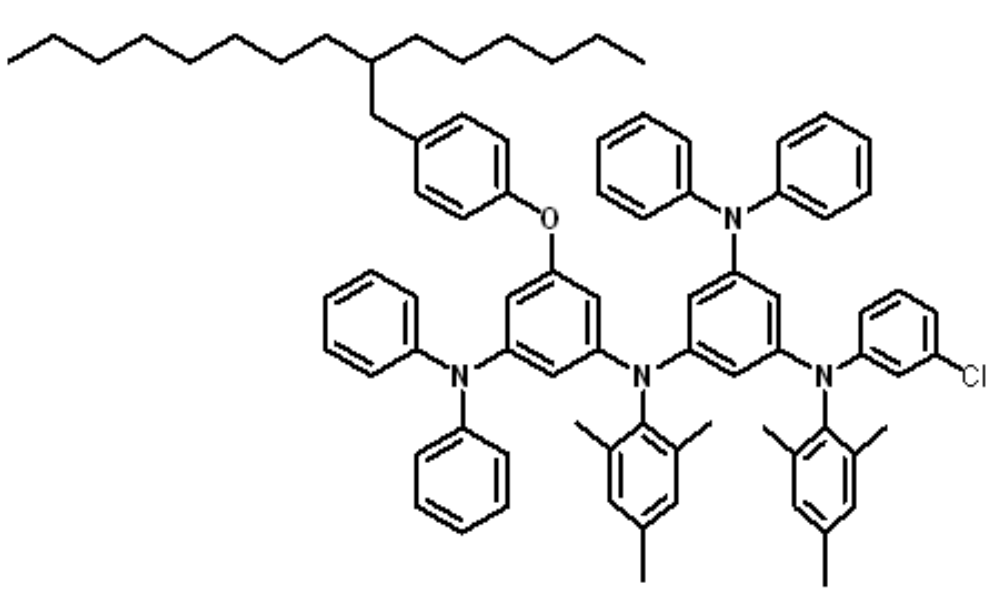

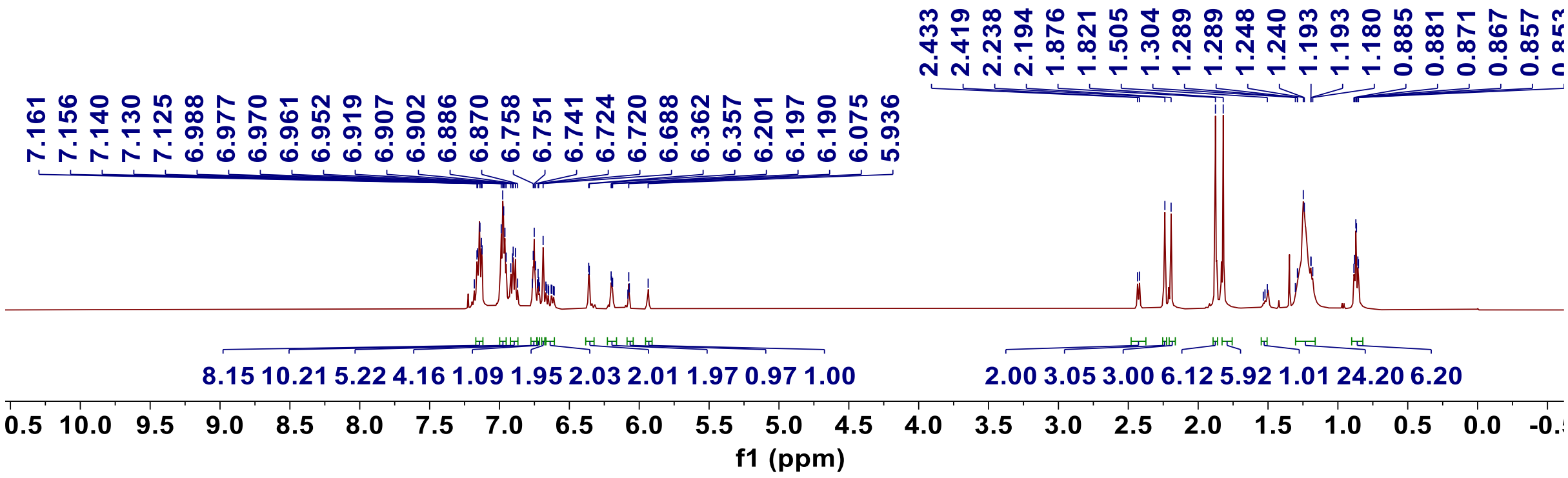

Figure S17. ^{1}H NMR spectrum of compound **12** in $CDCl_3$.

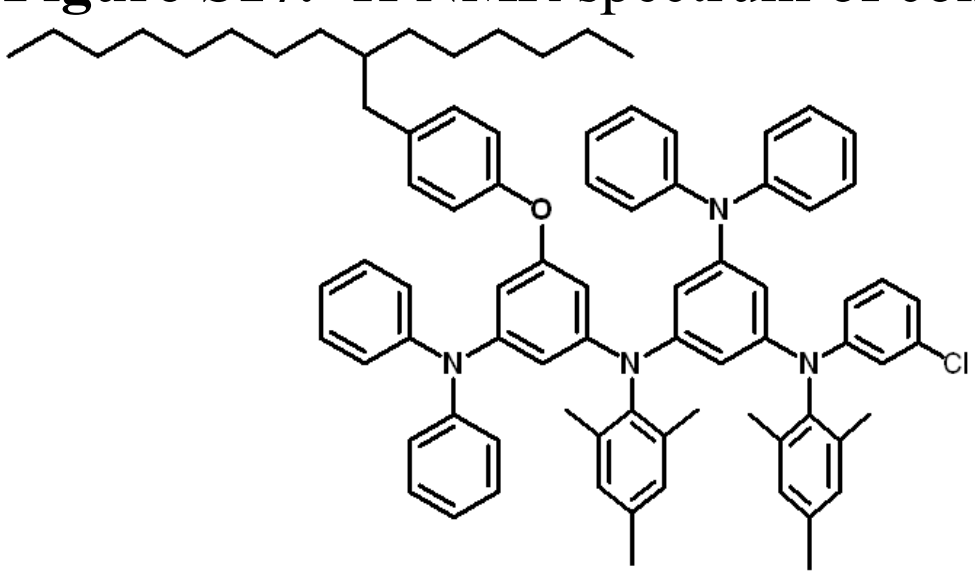

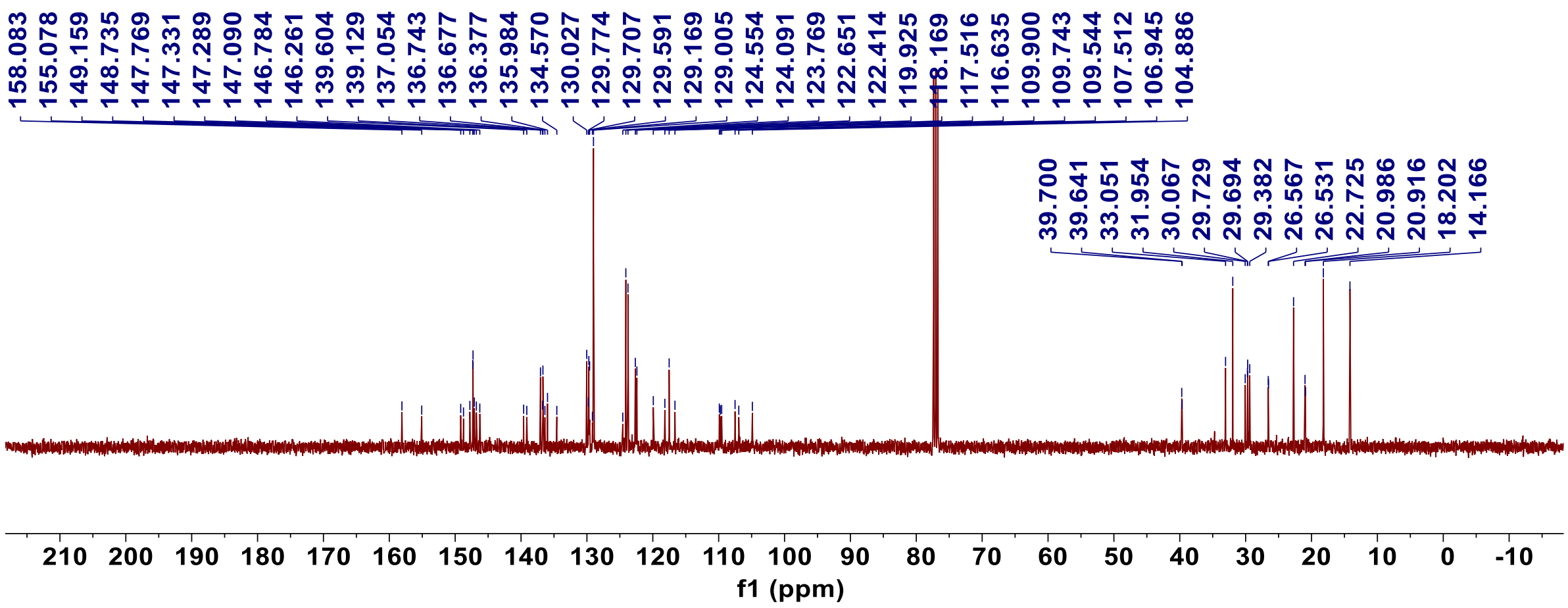

Figure S18. ^{13}C NMR spectrum of compound **12** in $CDCl_3$.

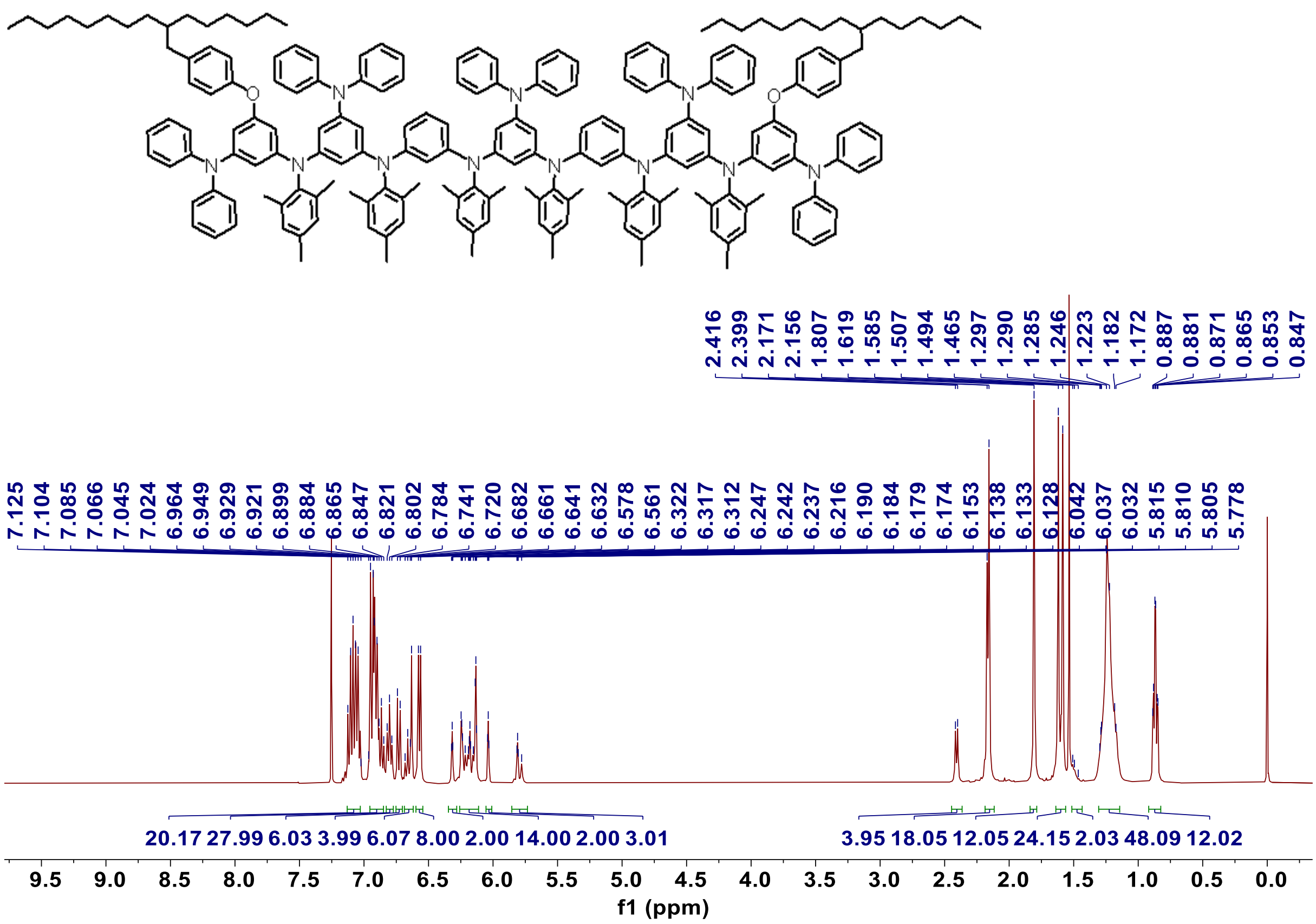

Figure S19. ^{1}H NMR spectrum of compound **14** in $CDCl_3$.

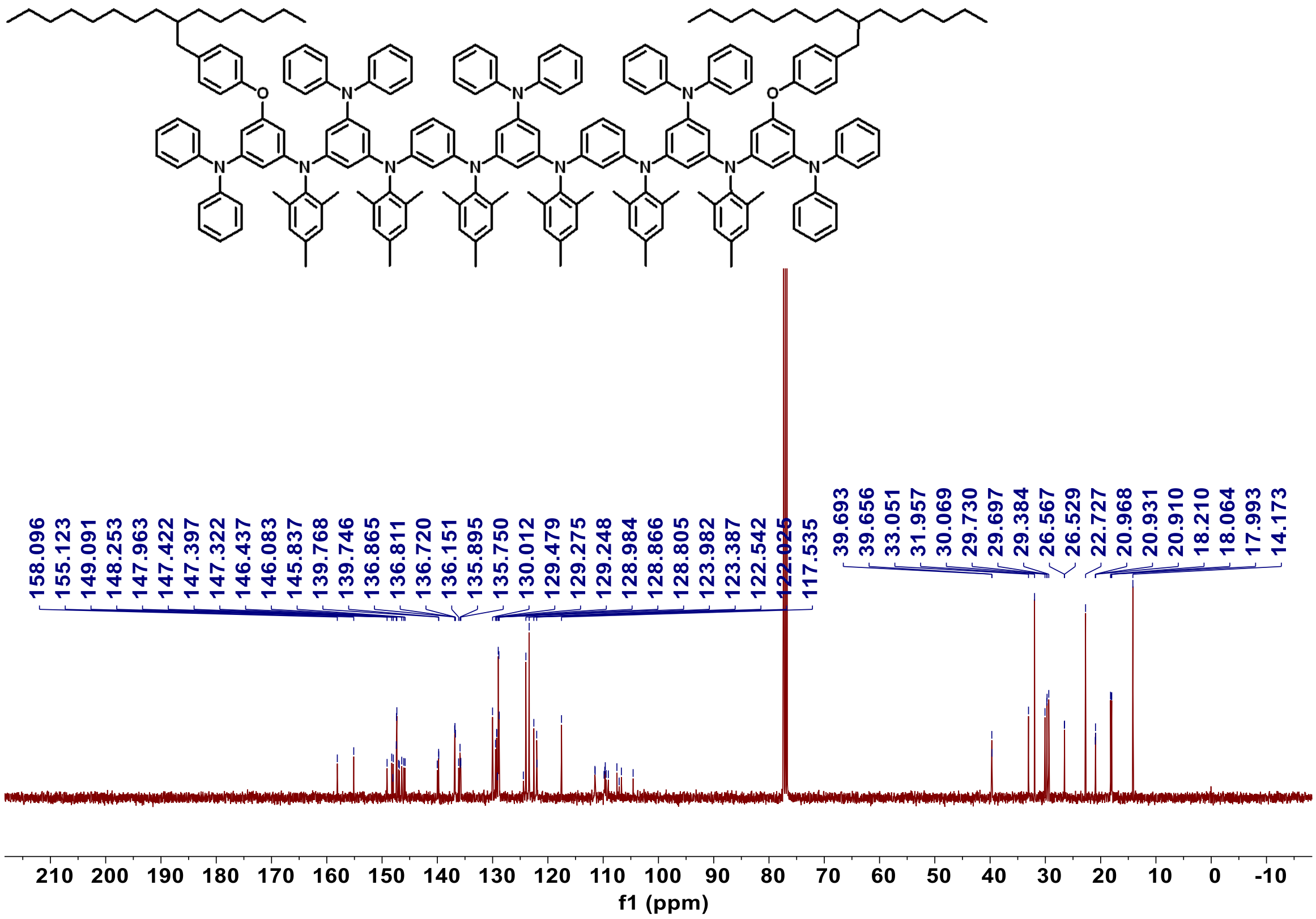

Figure S20. ^{13}C NMR spectrum of compound **14** in $CDCl_3$.

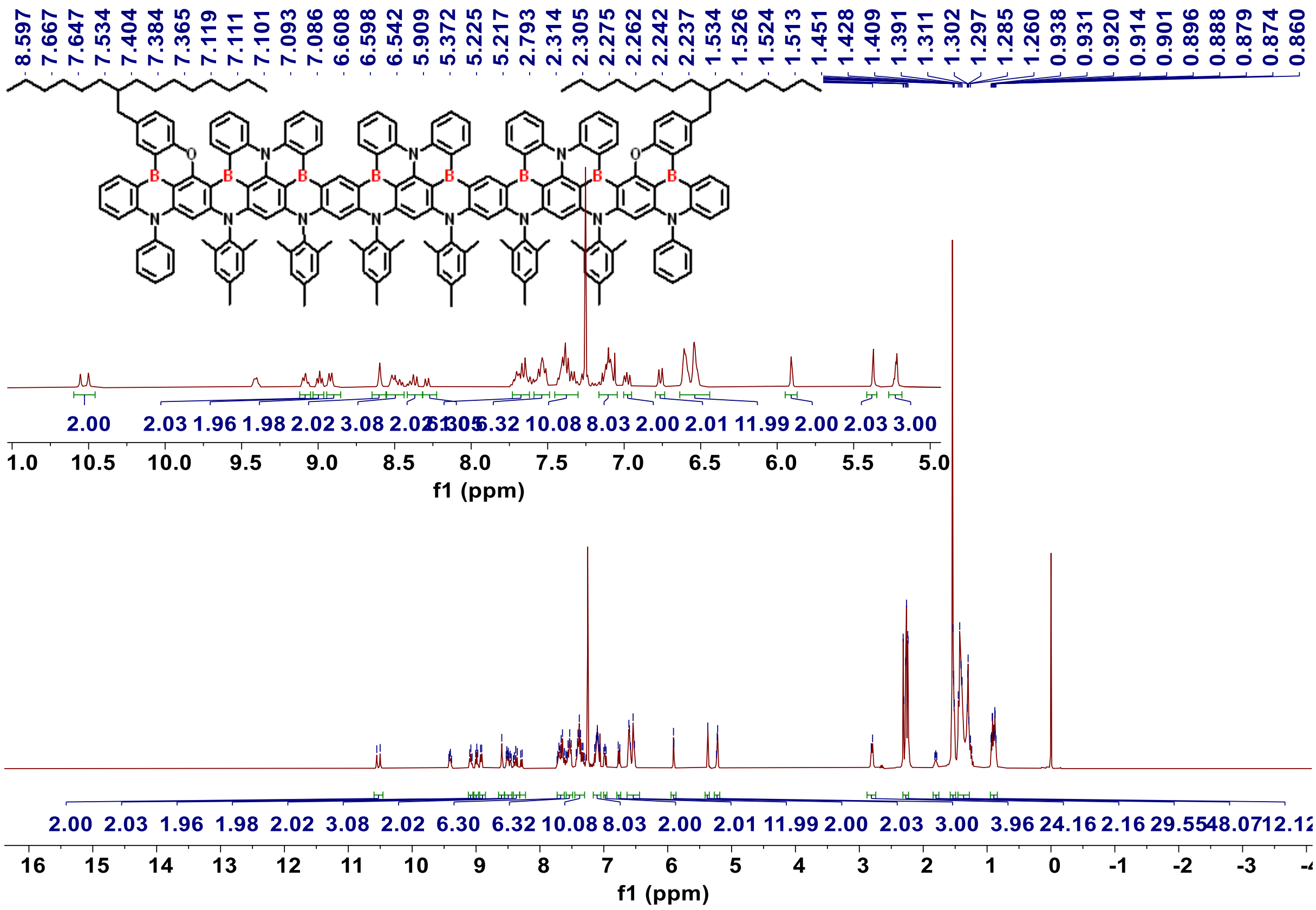

Figure S21. ^{1}H NMR spectrum of compound **DPA-B8** in $CDCl_3$.

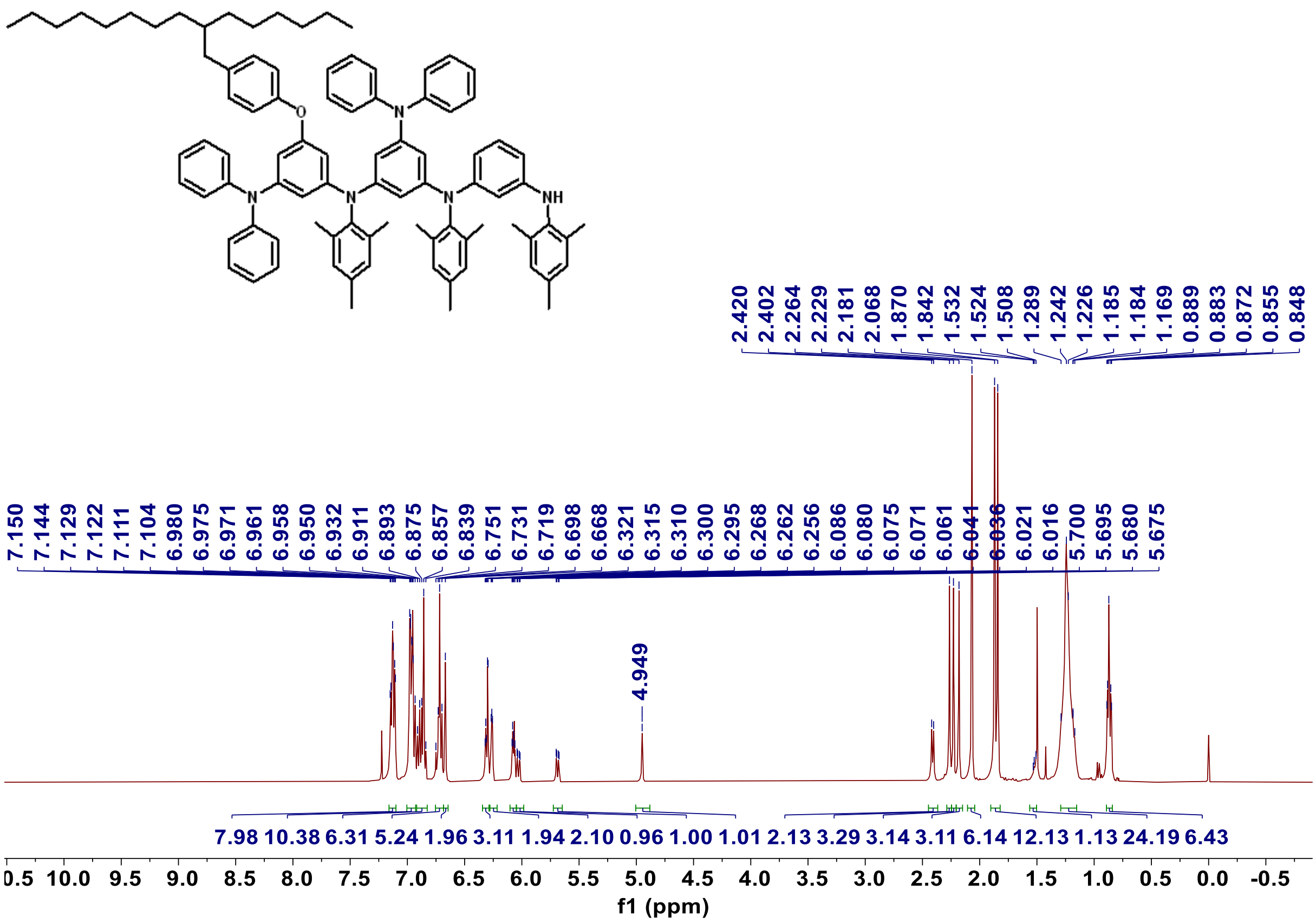

Figure S22 ^{1}H NMR spectrum of compound **15** in $CDCl_3$.

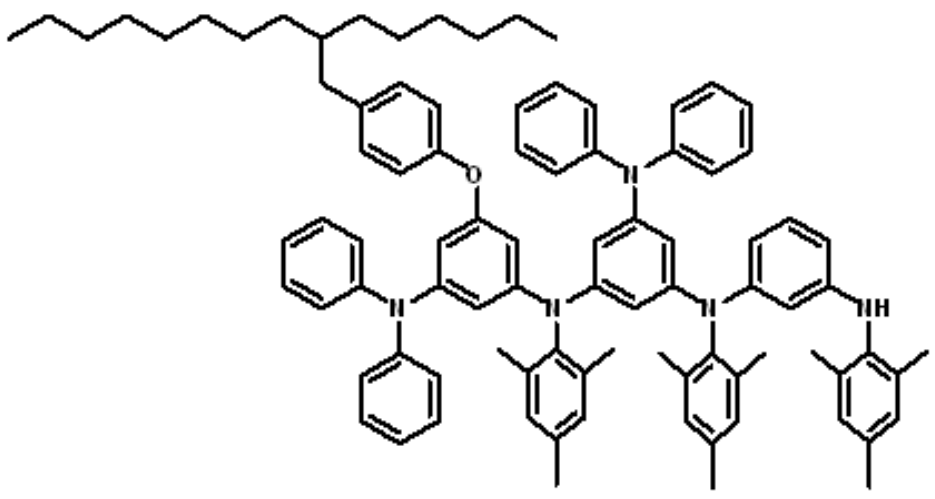

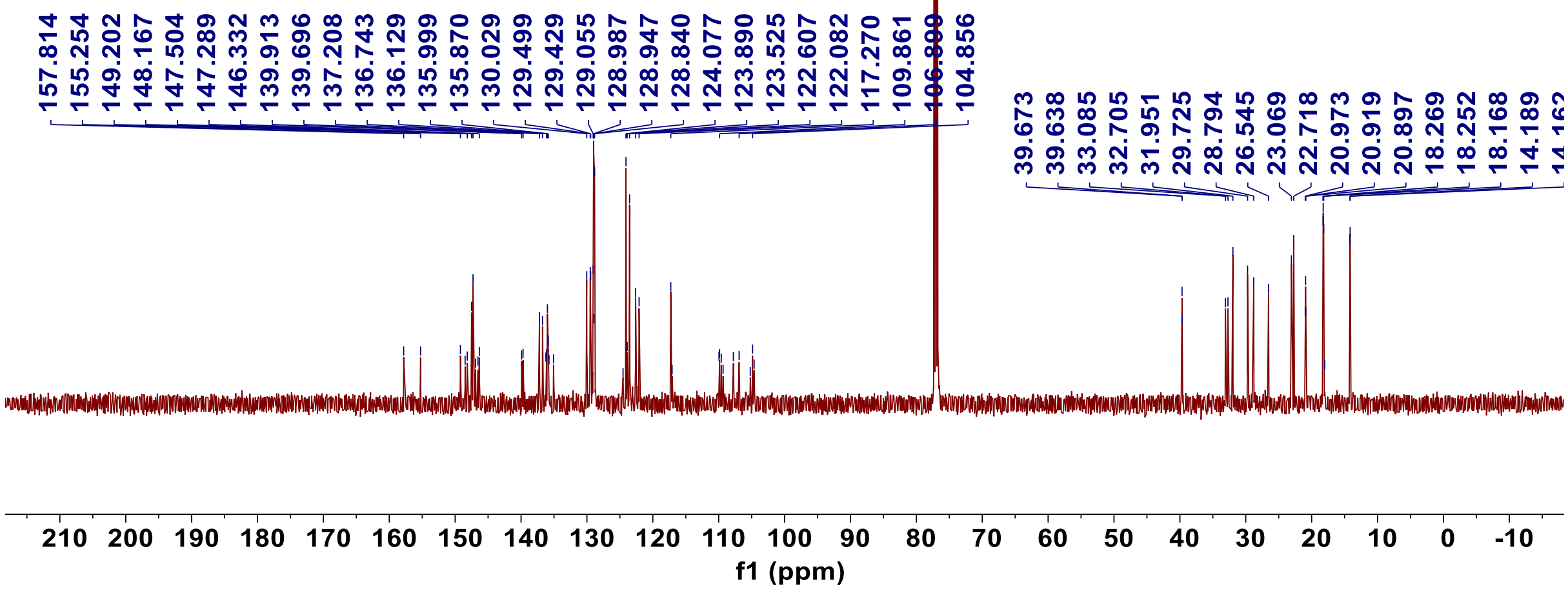

Figure S23. ^{13}C NMR spectrum of compound **15** in $CDCl_3$.

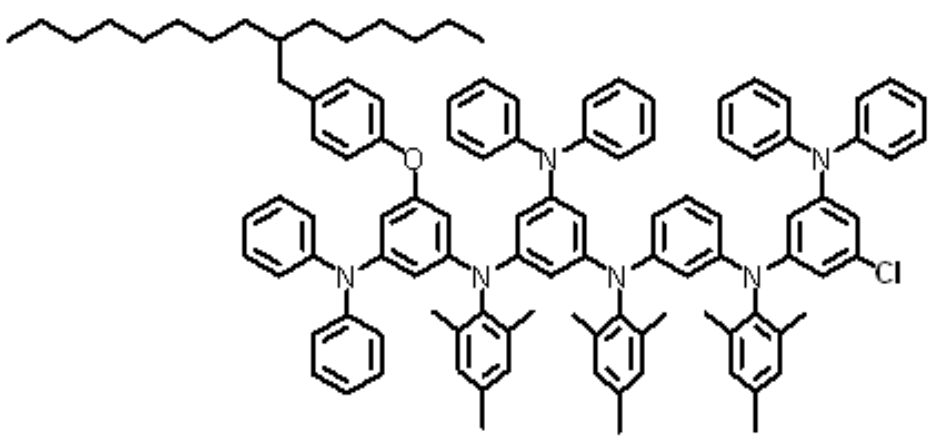

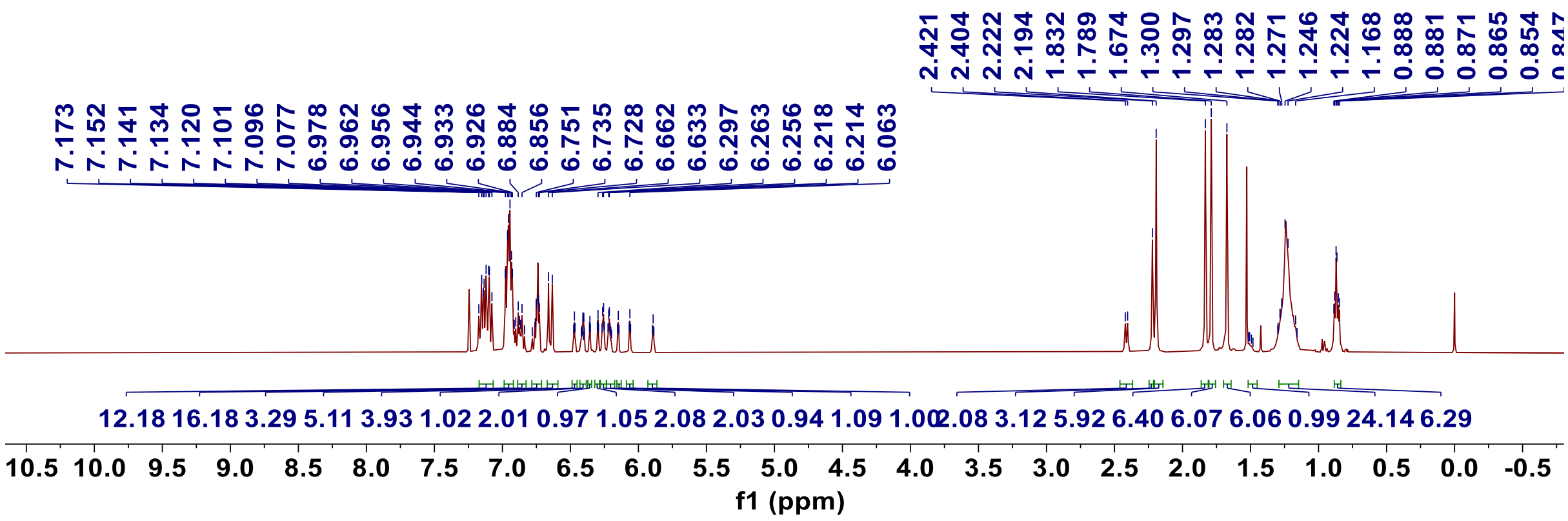

Figure S24. ^{1}H NMR spectrum of compound **16** in $CDCl_3$.

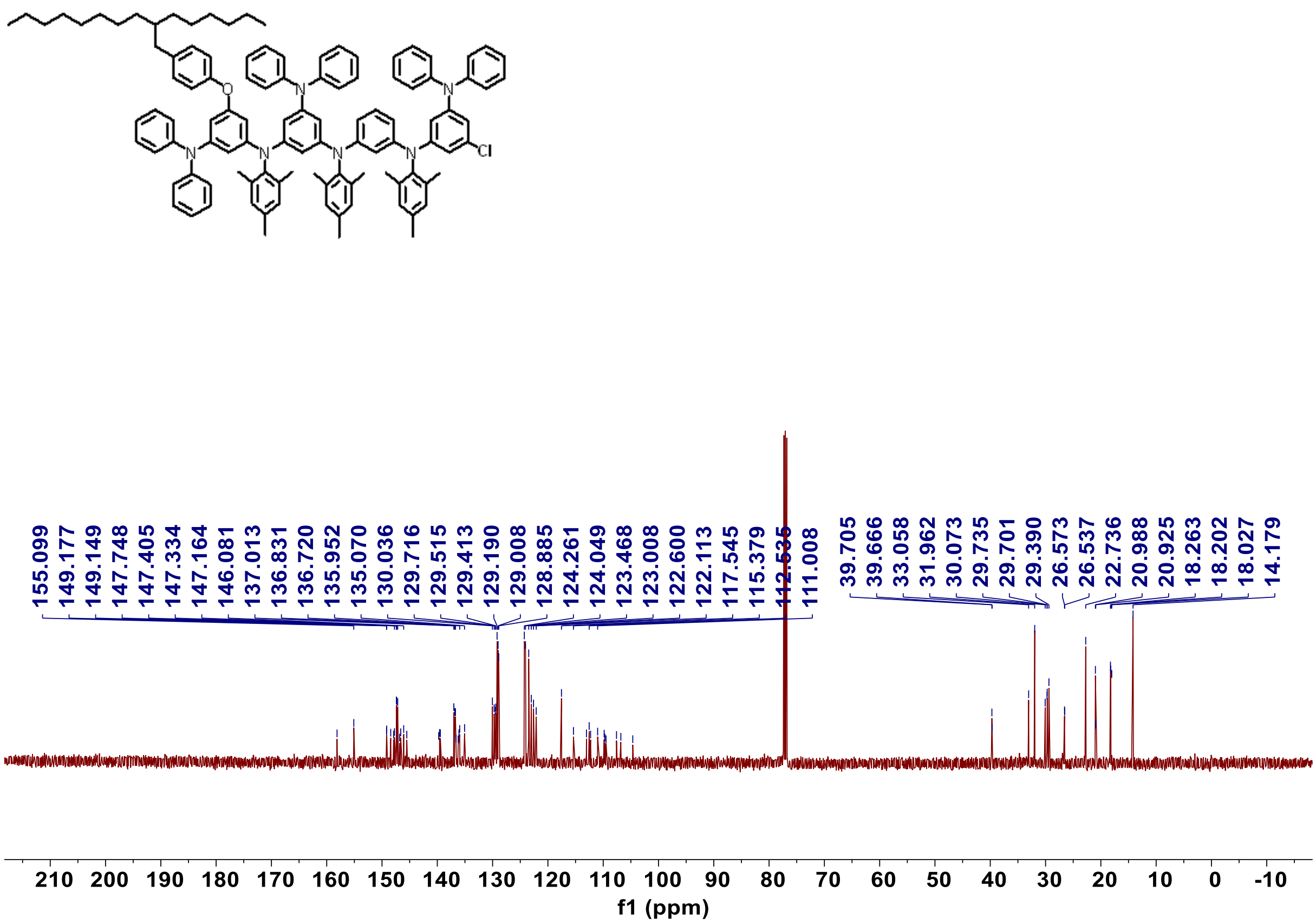

Figure S25. ^{13}C NMR spectrum of compound **16** in $CDCl_3$.

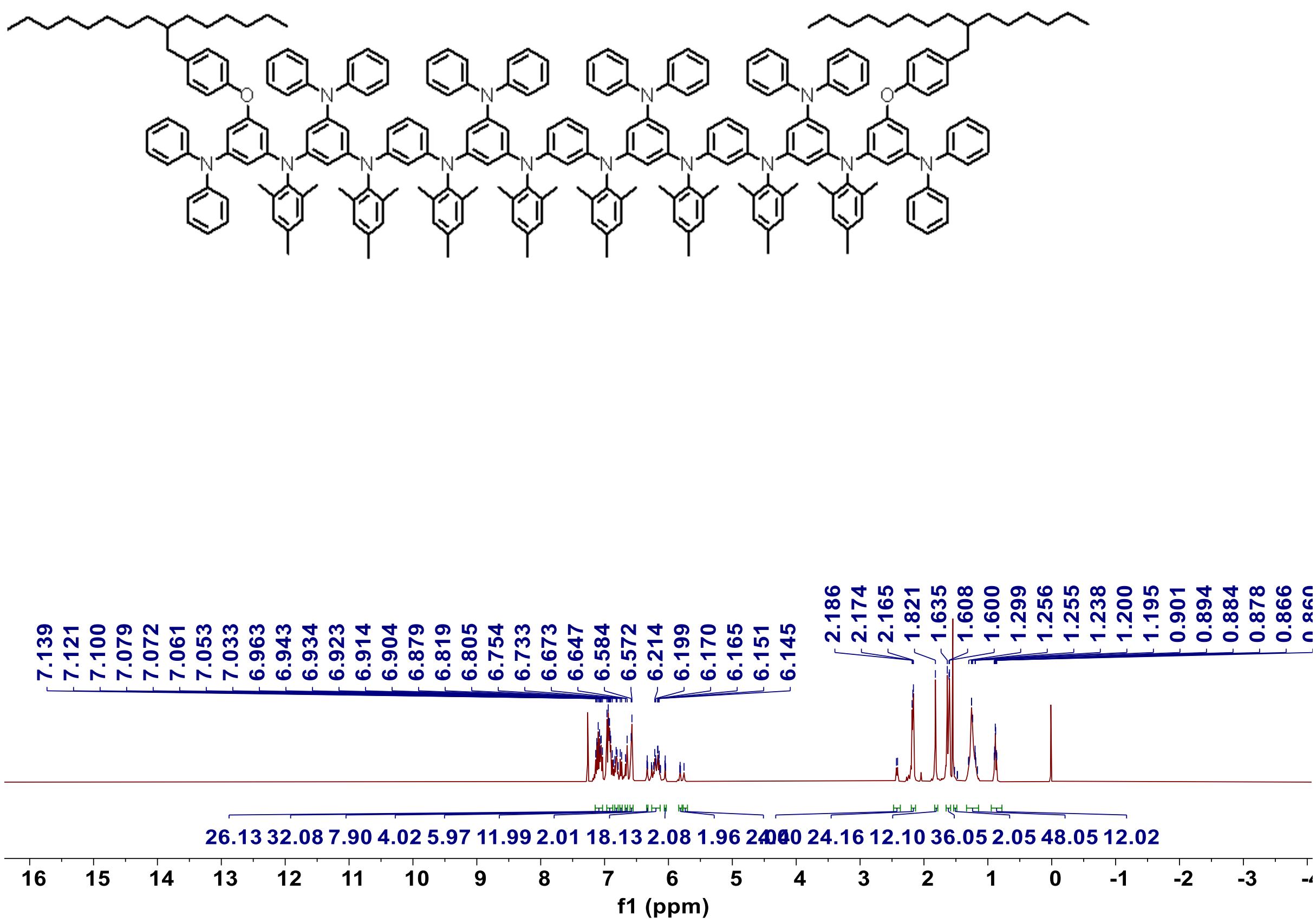

Figure S26. ^{1}H NMR spectrum of compound **17** in $CDCl_3$.

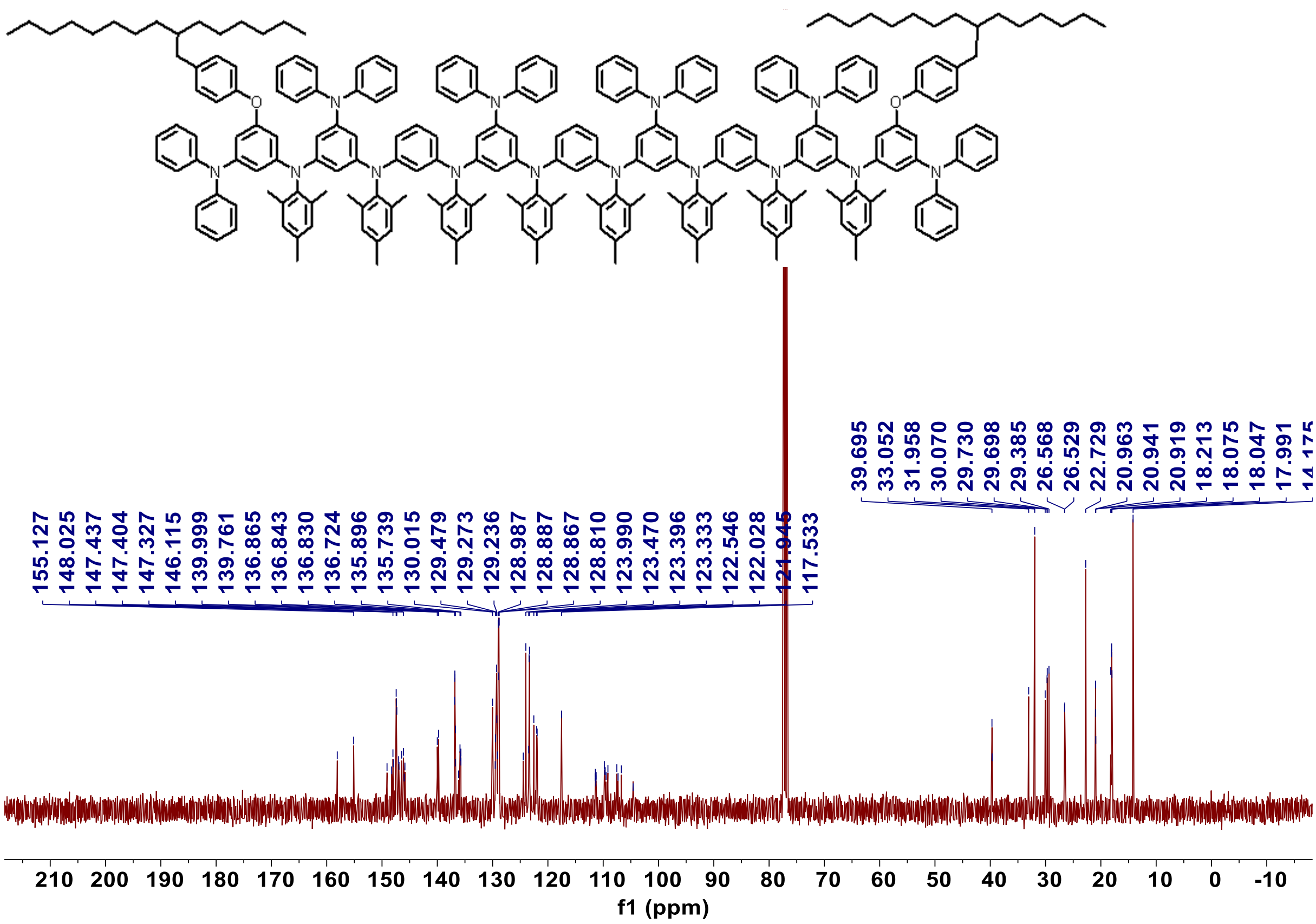

Figure S27. ^{13}C NMR spectrum of compound **17** in $CDCl_3$.

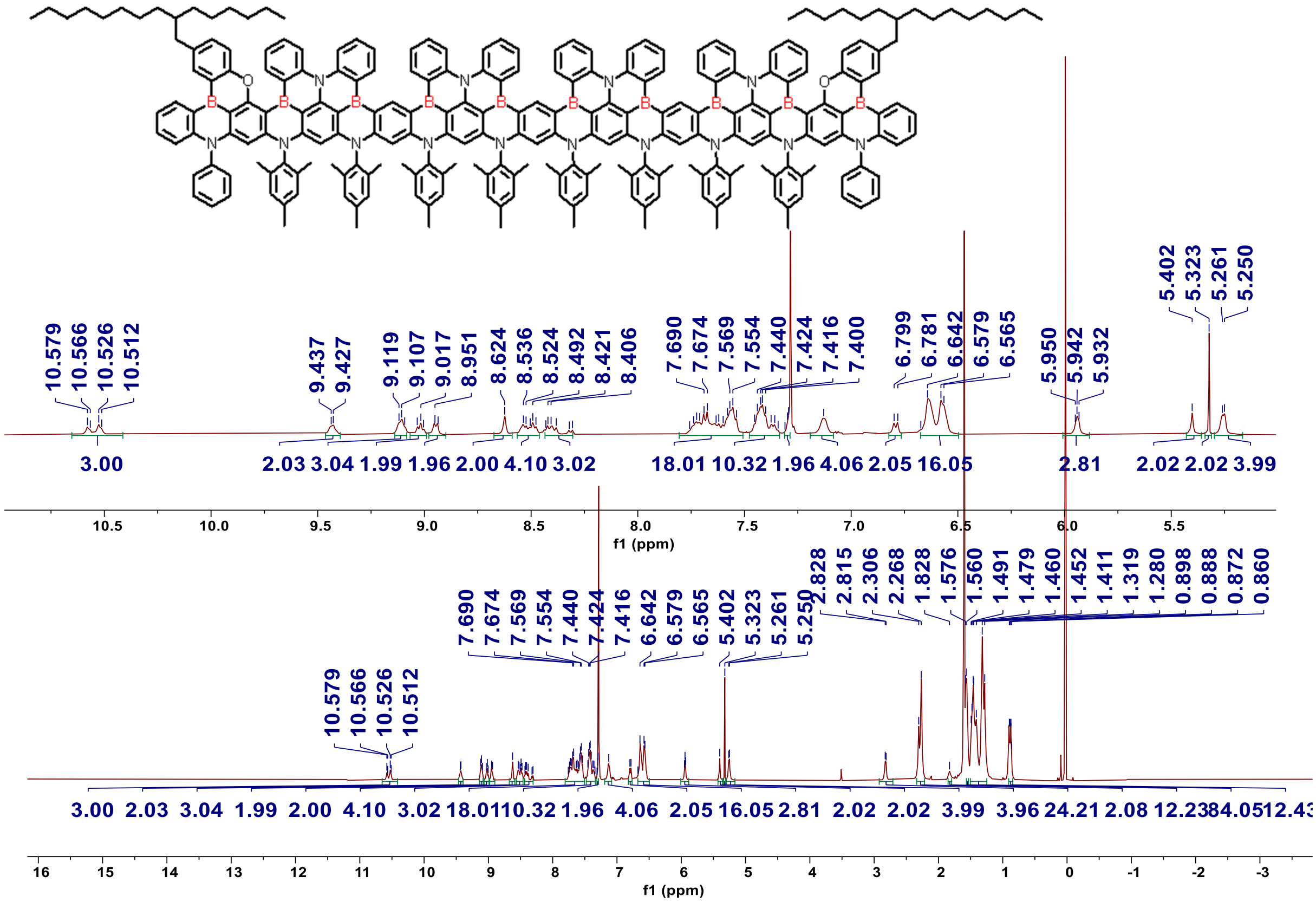

Figure S28. ^{1}H NMR spectrum of compound **DPA-B10** in $CDCl_3$.

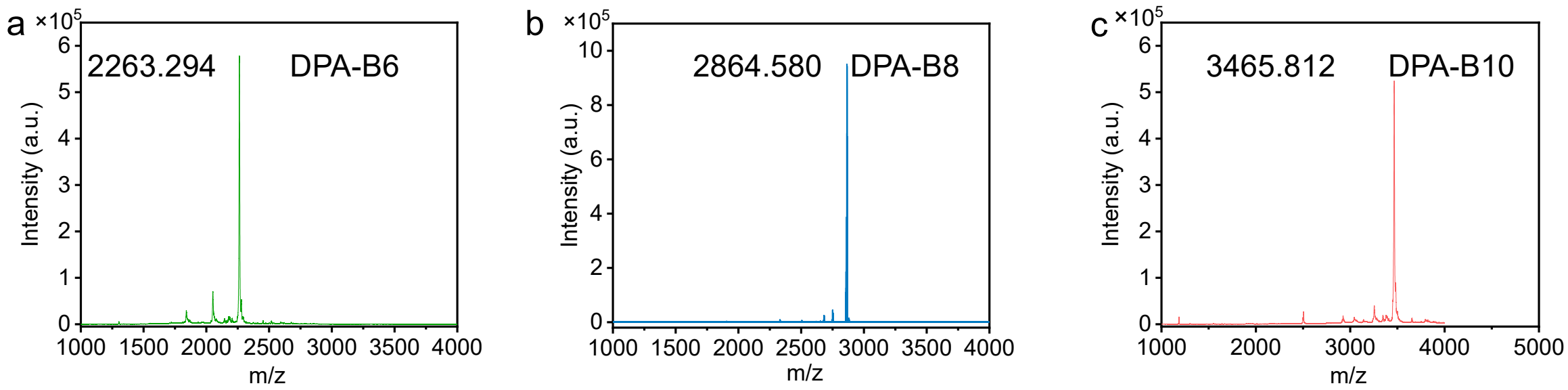

Figure S29. MALDI-TOF spectra of **DPA-B6** (a), **DPA-B8** (b), and **DPA-B10** (c).

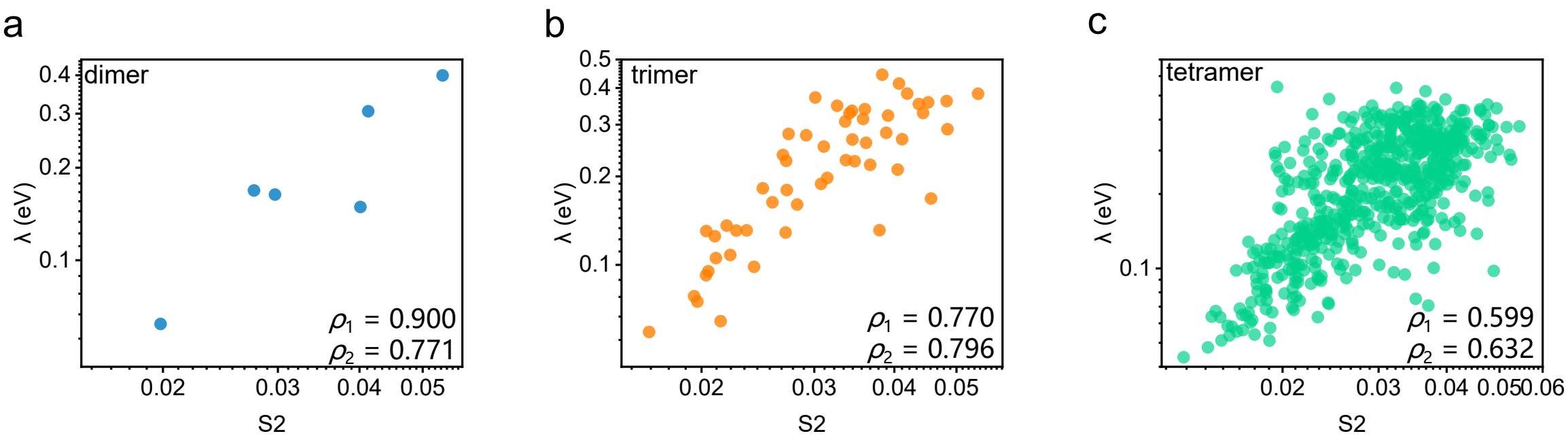

Figure S30. Correlation of dimeric (a), trimeric (b), and tetrameric (c) reorganization energies with respect to S2 (Pearman correlation ρ_1, Spearman correlation ρ_2).

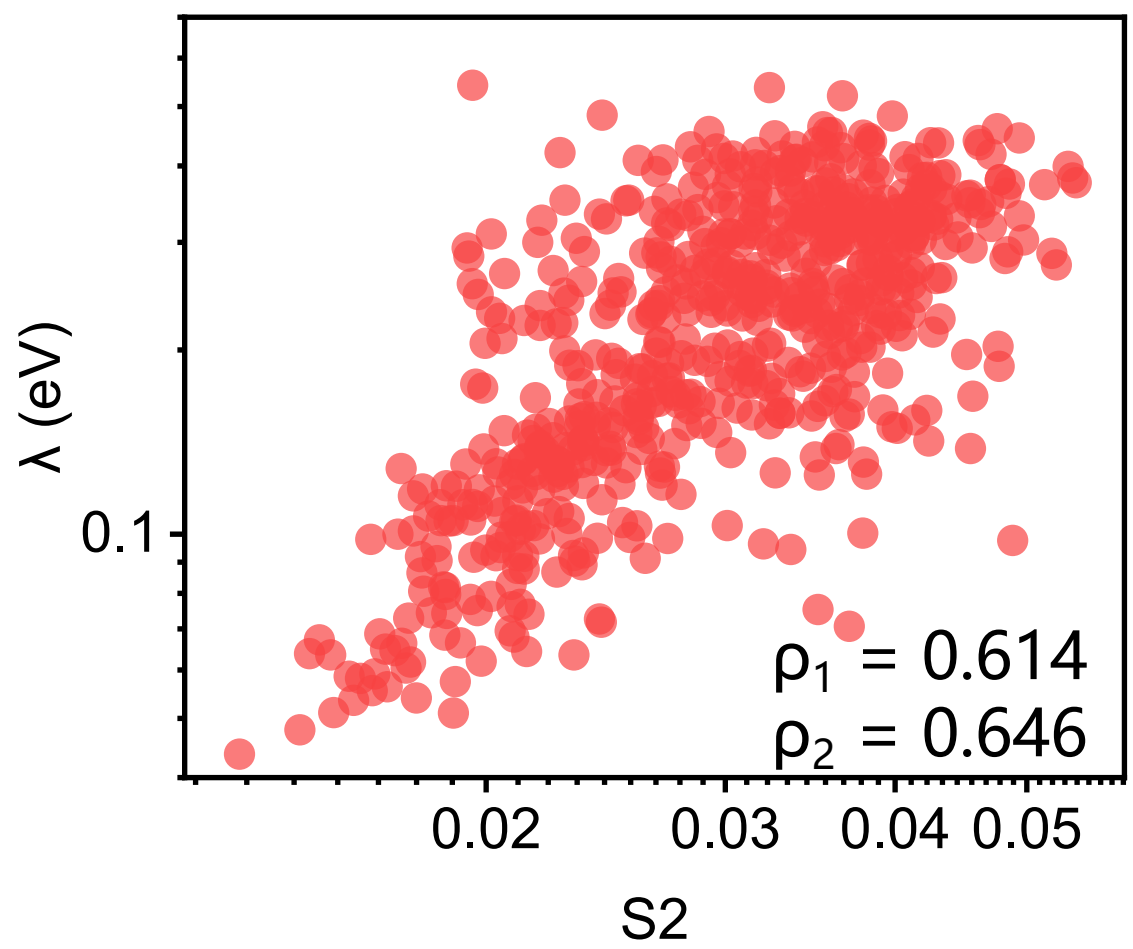

Figure S31. Correlation of the reorganization energies of all oligomers with respect to S2 (Pearman correlation $\rho_1 = 0.614$, Spearman correlation $\rho_2 = 0.646$).

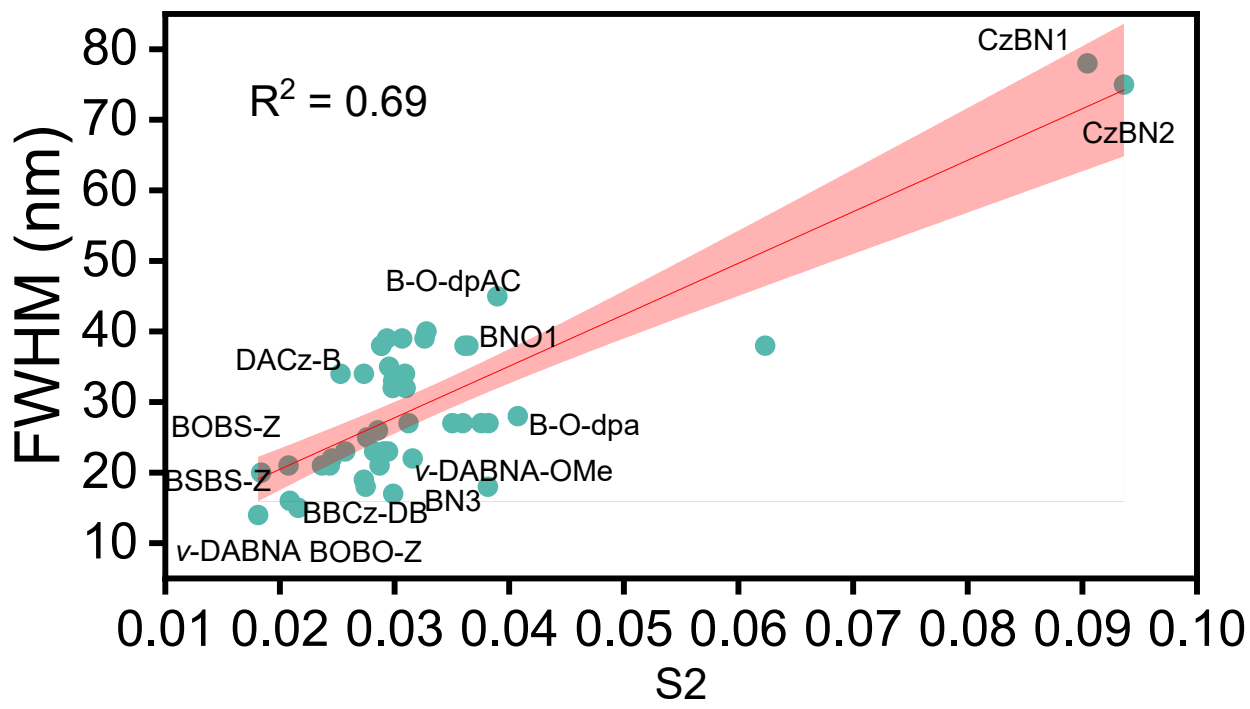

Figure S32. Correlation between FWHM and S2 for representative MR-TADF emitters.

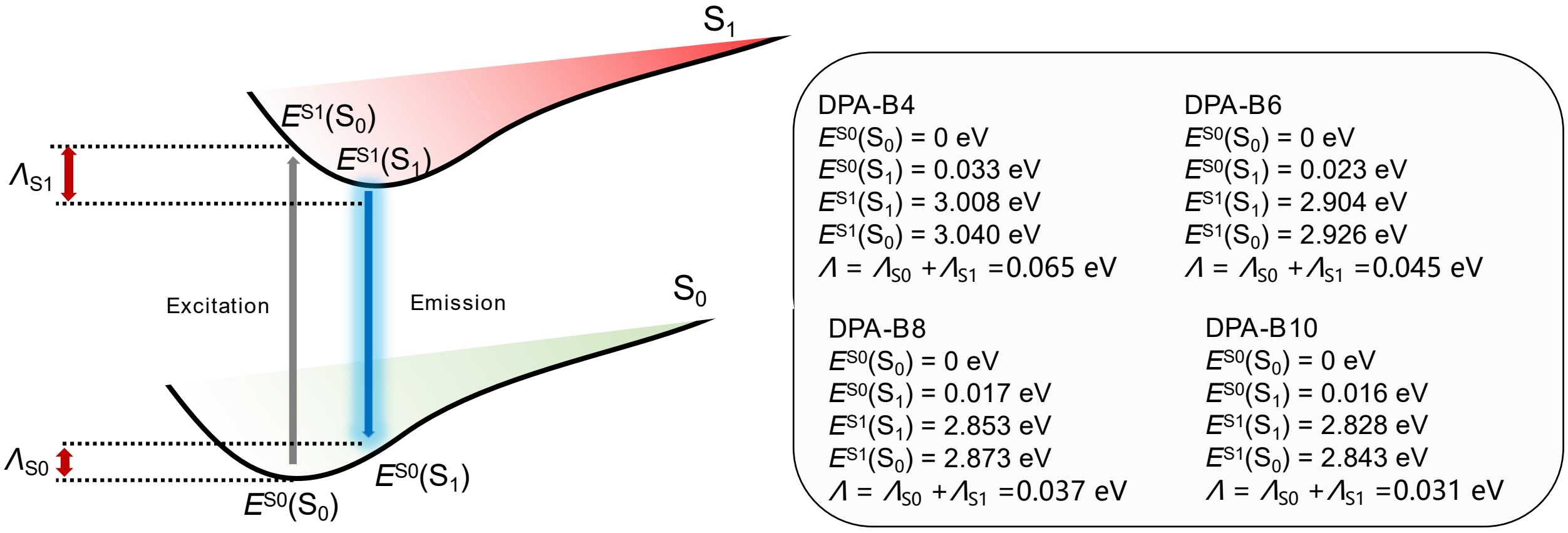

Figure S33. Reorganization energies (λ) values of DPA-Bn calculated by four-point method.

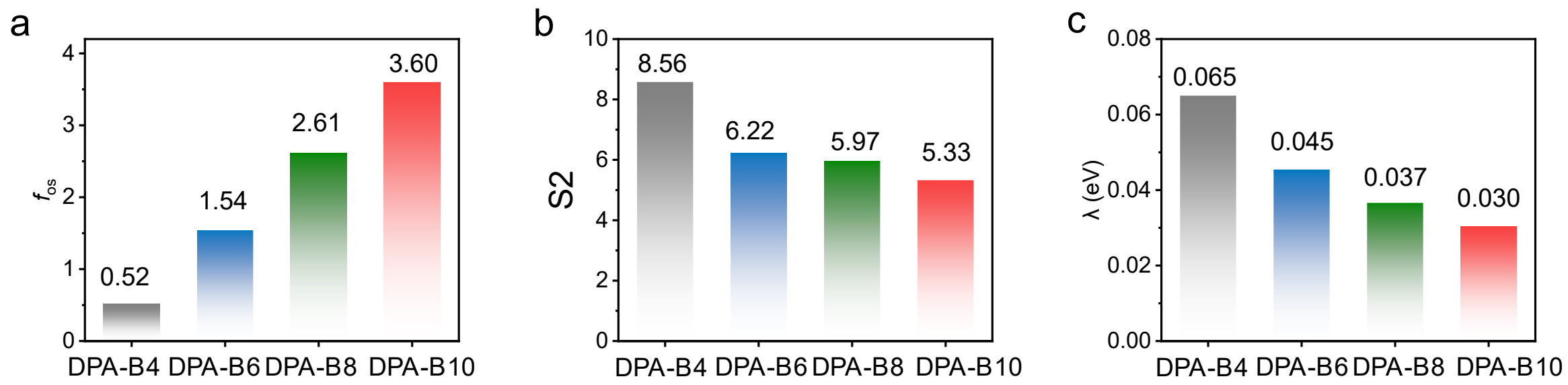

Figure S34. Oscillator strength (f_{os}), square of bond-order change (S2), and reorganization energies (λ) of **DPA-B4**, **DPA-B6**, **DPA-B8**, and **DPA-B10**.

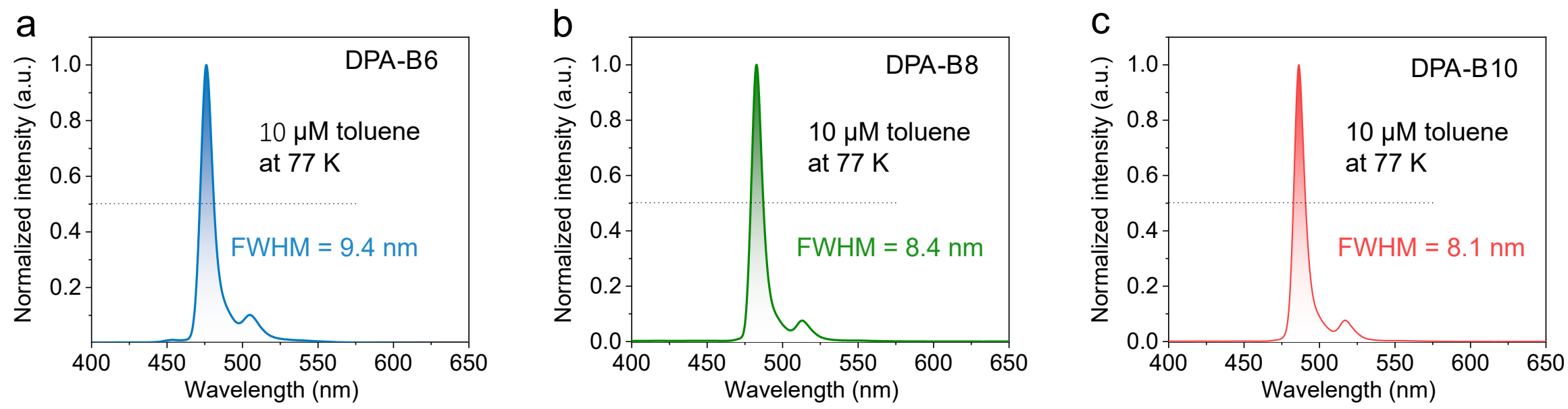

Figure S35. Normalized PL spectra of **DPA-B6** (a), **DPA-B8** (b), and **DPA-B10** (c) in toluene (1 × 10^{-5} M) at 77 K.

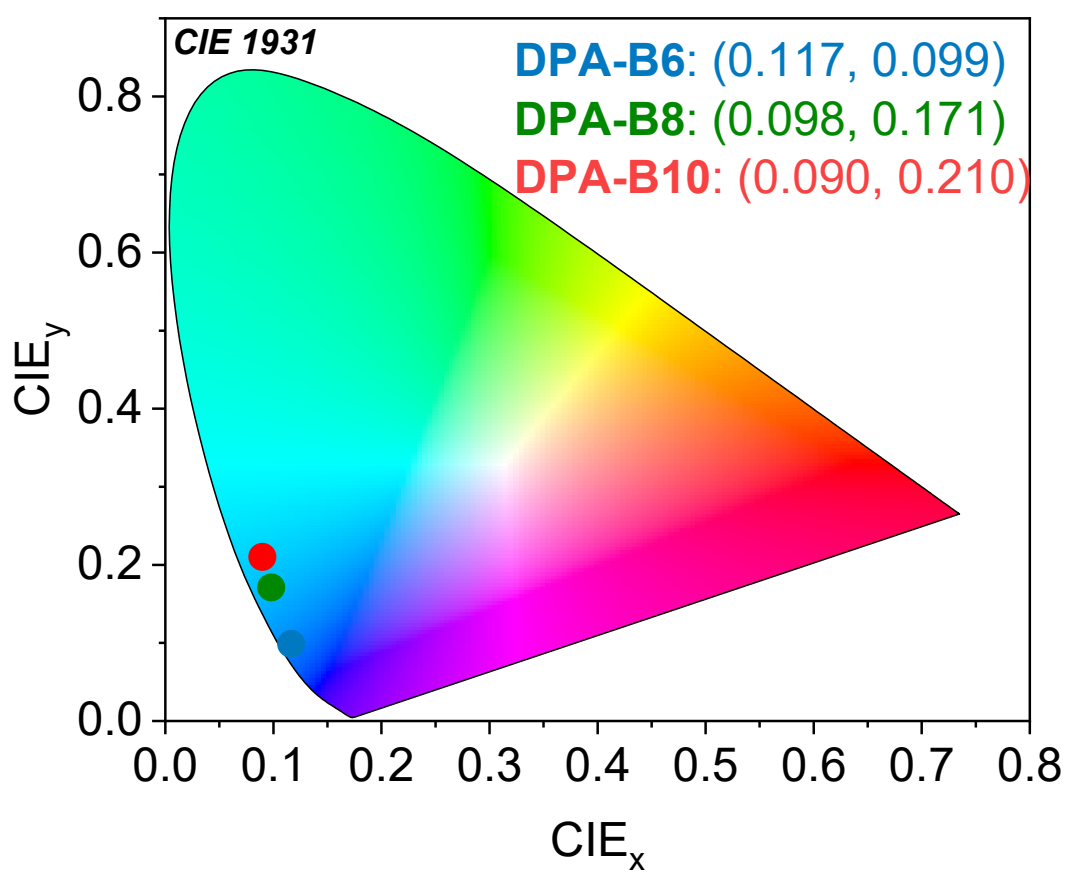

Figure S36. CIE coordinates of DPA-Bn series in toluene (1 × 10^{-5} M) at room temperature.

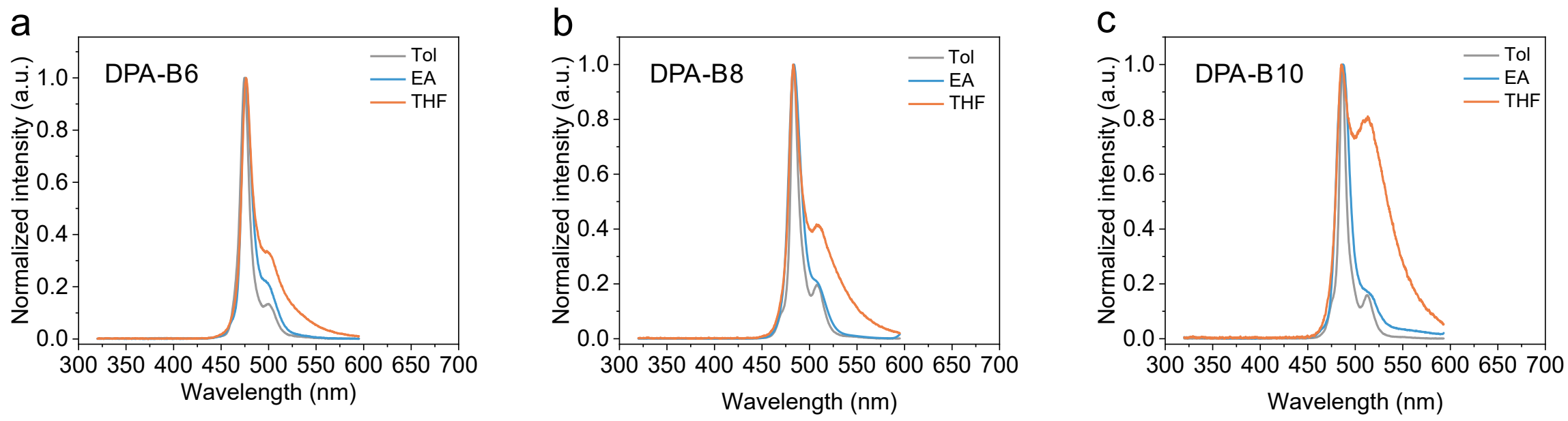

Figure S37. Normalized fluorescence spectra of DPA-Bn in different solvents at 1 × 10^{-5} M (Tol: toluene, EA: ethyl acetate, and THF: tetrahydrofuran).

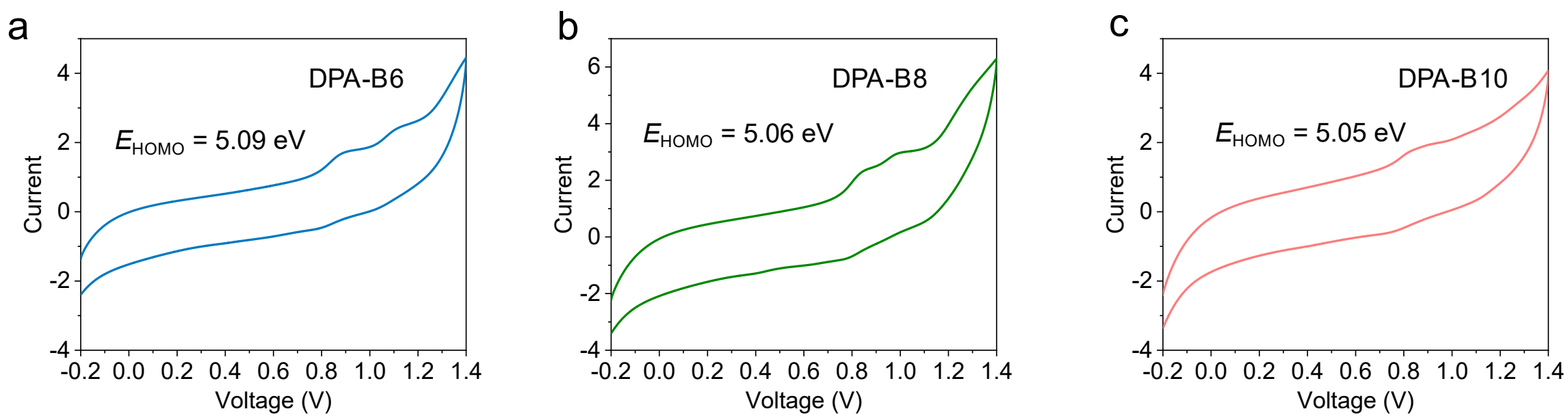

Figure S38. cyclic voltammograms curves of **DPA-B6** (a), **DPA-B8** (b), and **DPA-B10** (c) in dichloromethane.

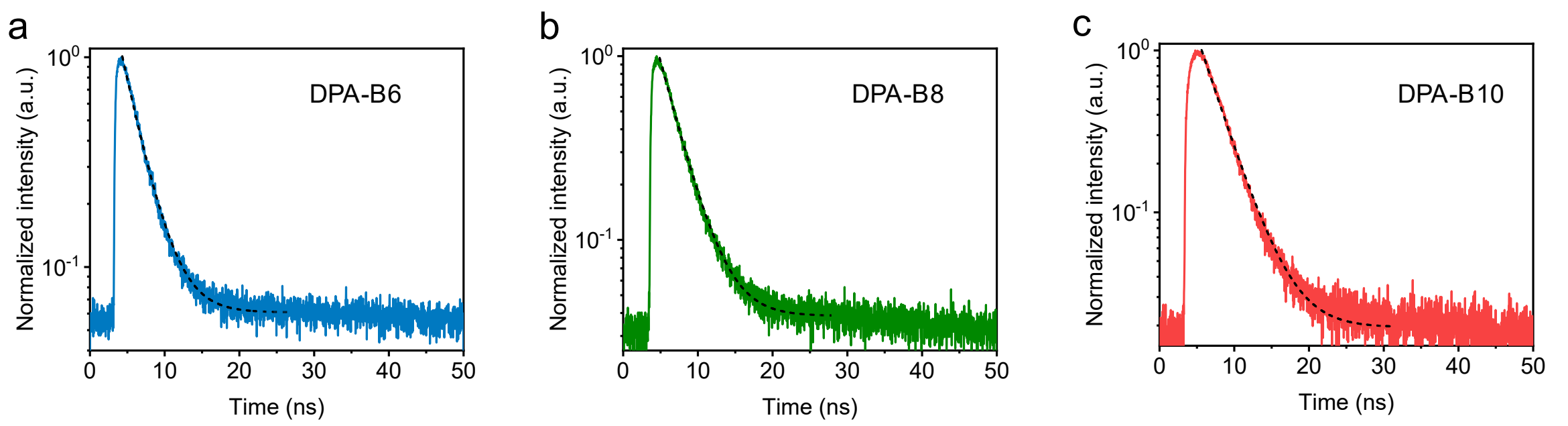

Figure S39. Transient photoluminescence decay curves of **DPA-B6** (a), **DPA-B8** (b), **DPA-B10** (c) in dilute toluene at ns-scale, theoretical decay curves from bi-exponential fit procedure are shown as black dash lines.

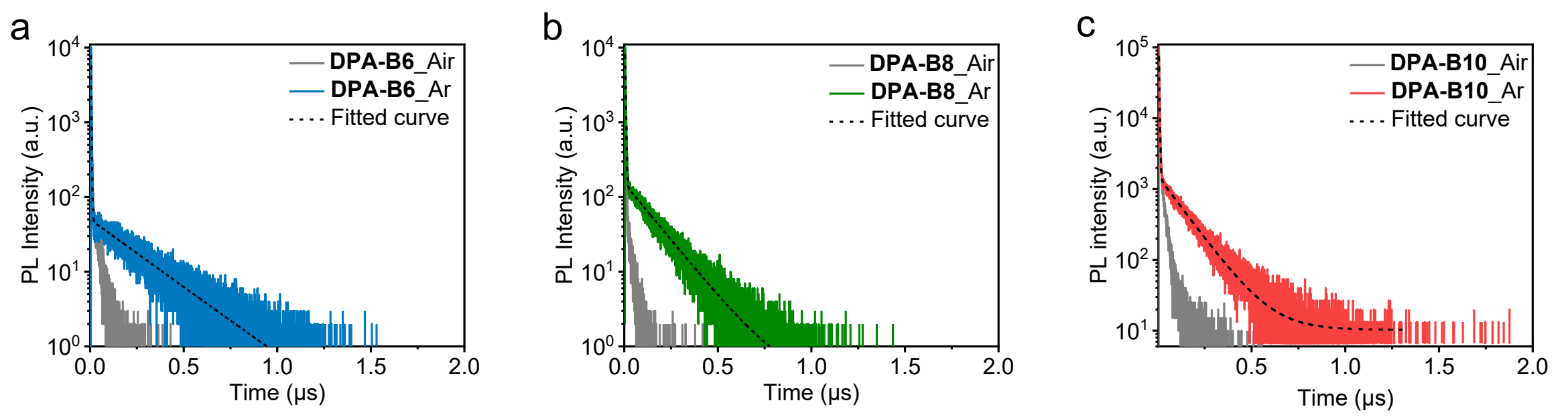

Figure S40. Transient photoluminescence decay curves of **DPA-B6** (a), **DPA-B8** (b), **DPA-B10** (c) in dilute toluene at μs-scale, theoretical decay curves from bi-exponential fit procedure are shown as black dash lines.

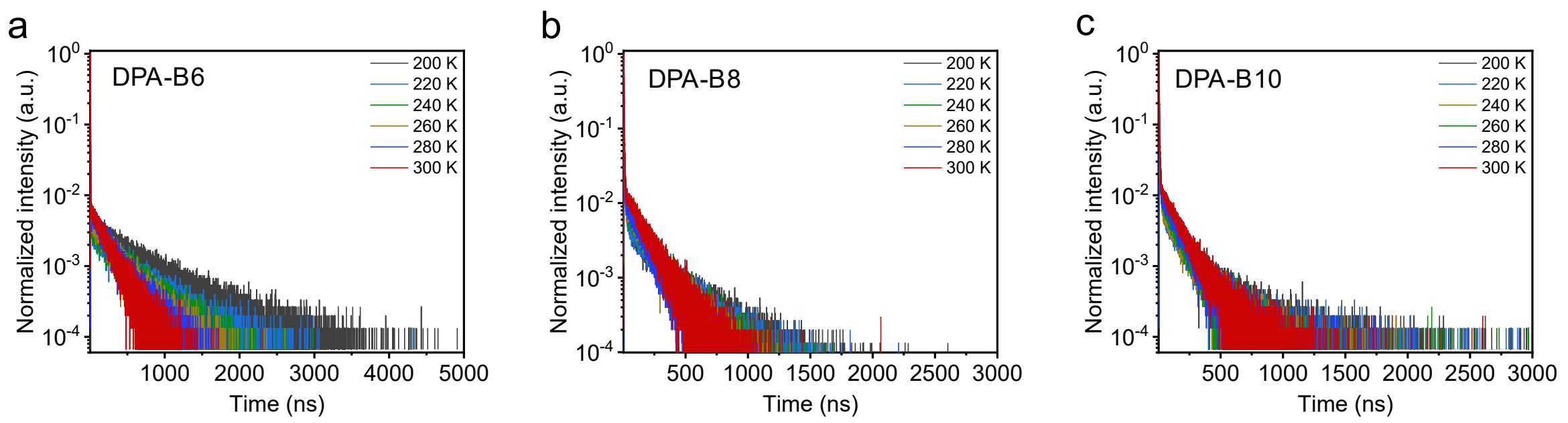

Figure S41. Temperature-dependent transient decay characteristics of **DPA-B6** (a), **DPA-B8** (b), and **DPA-B10** (c) in toluene (1 $\times 10^{-5}$ M).

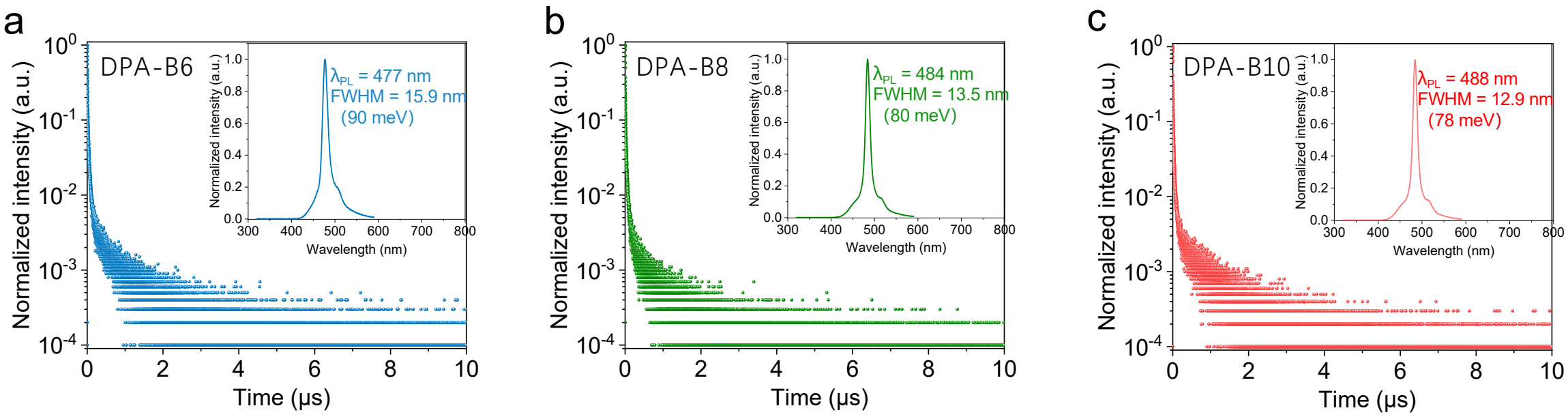

Figure S42. Transient PL decay curves of **DPA-B6** (a), **DPA-B8** (b), and **DPA-B10** (c) doped films (Insert: PL spectra of doped films).

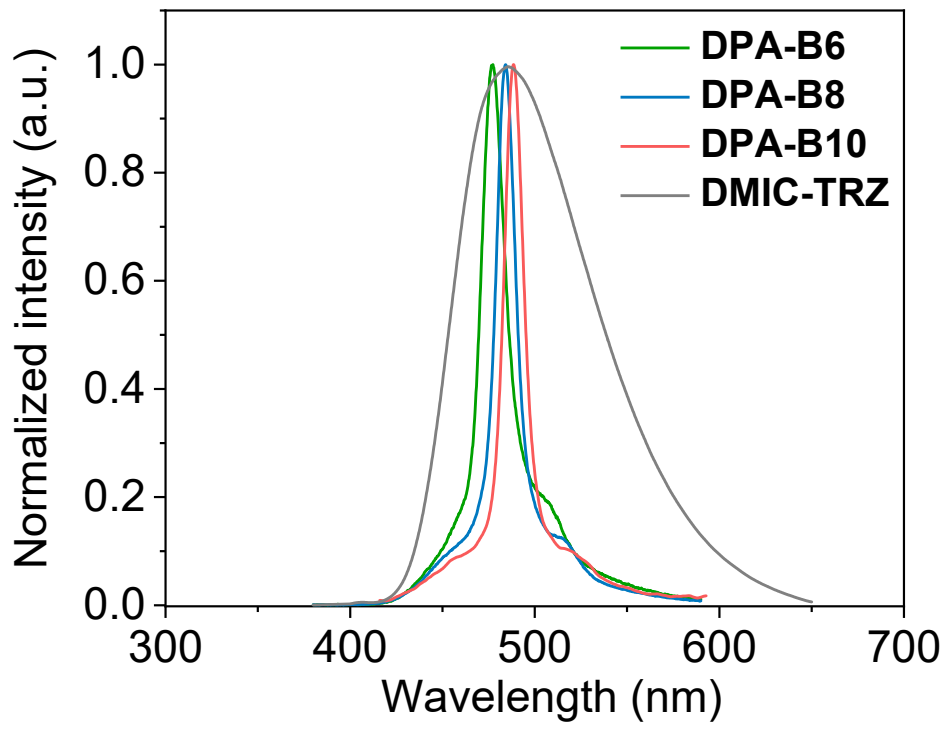

Figure S43. Photoluminescence spectra of neat DMIC-TRZ and DPA-B*n*-doped films (0.6 wt% in DMIC-TRZ) at 300 K. The doped films show weak residual host emission under optical excitation, while these host-related features are negligible in the EL spectra, consistent with more efficient host-to-dopant exciton transfer under electrical operation.

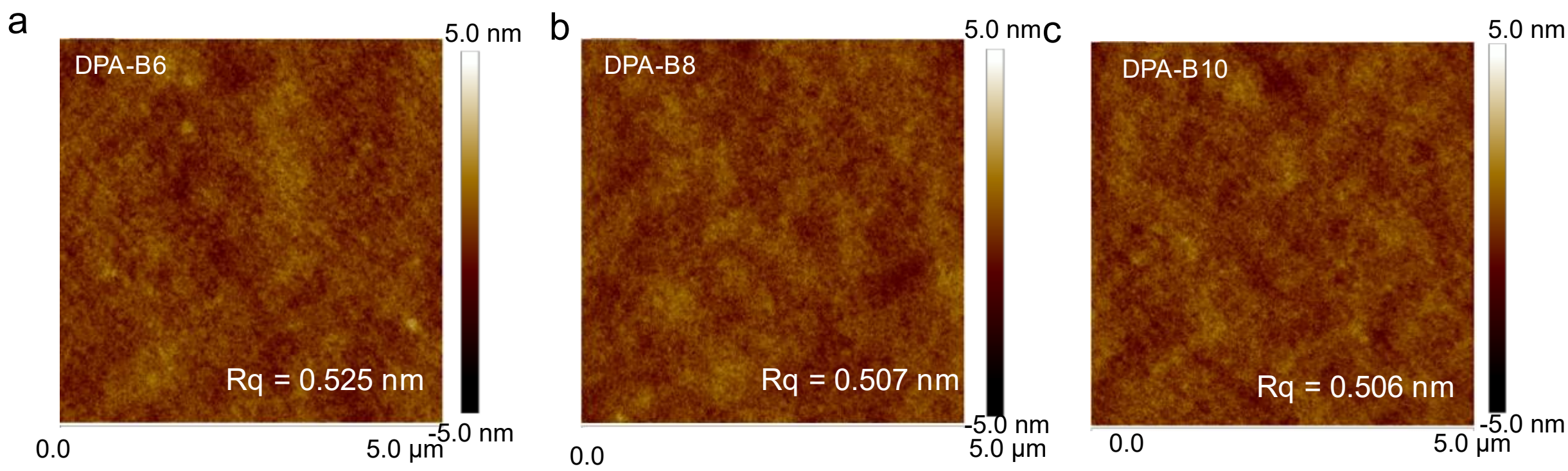

Figure S44. Photographs of surface morphology for DPA-B6 (a), DPA-B8 (b), and DPA-B10 (c) doped in DMIC-TRZ host (0.6 wt%) measured by atomic force microscopy (AFM).

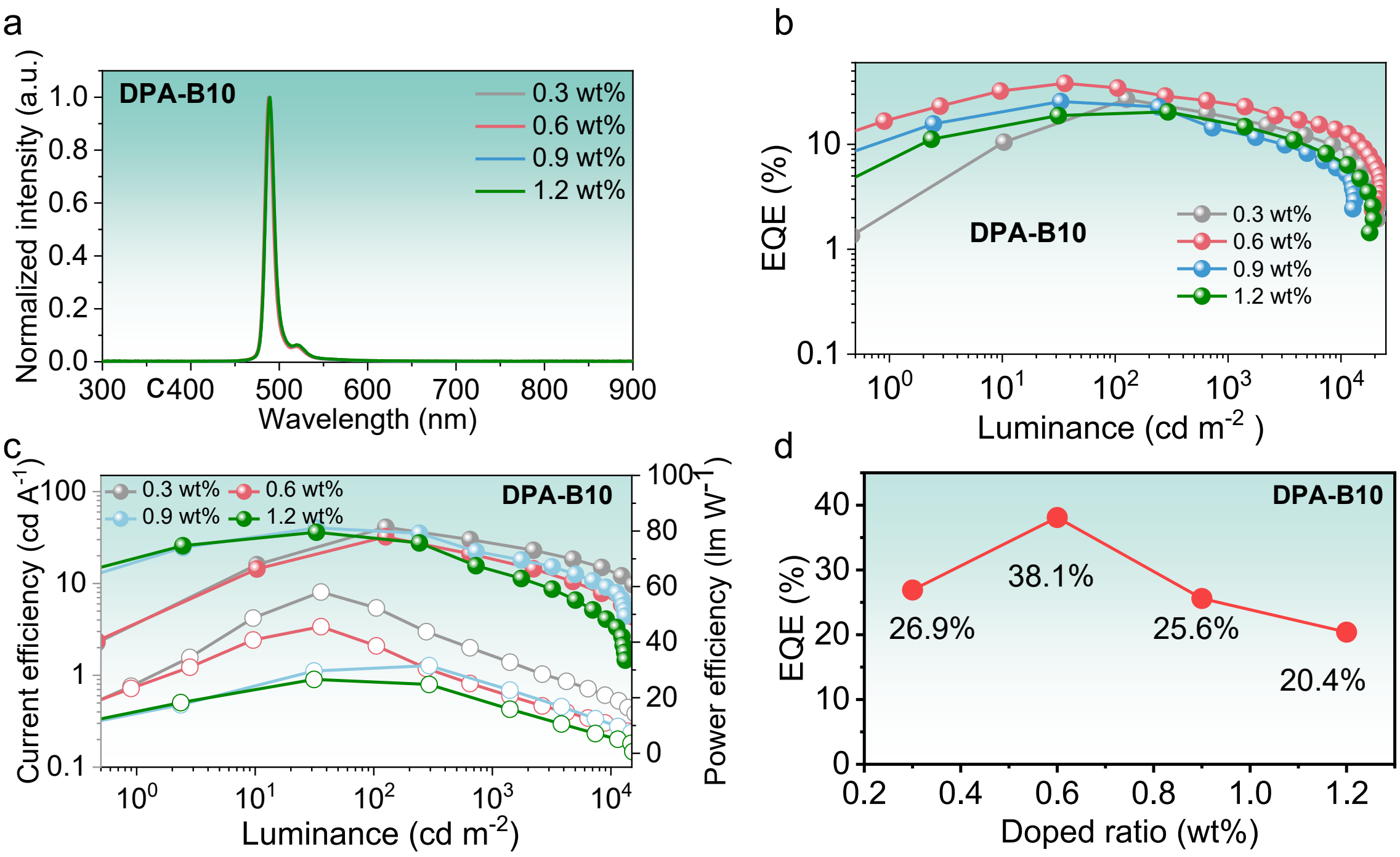

Figure S45. EL performance of DPA-B10 at different doped concentration. EL spectra (a); Maximum EQE versus luminance characteristics (b); Current efficiency and power efficiency versus luminance curves (c); Maximum EQE versus doped ratio curve (d).

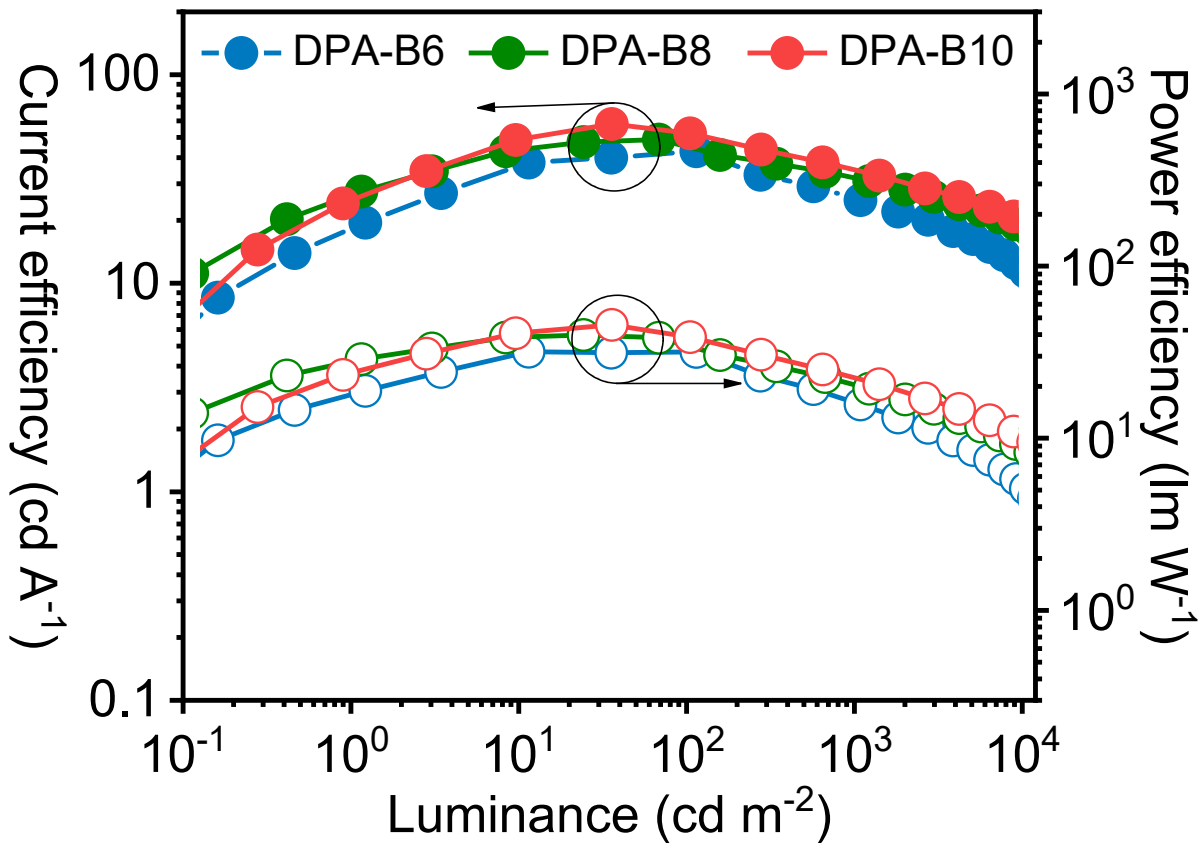

Figure S46. Current efficiency and power efficiency versus luminance characteristics.

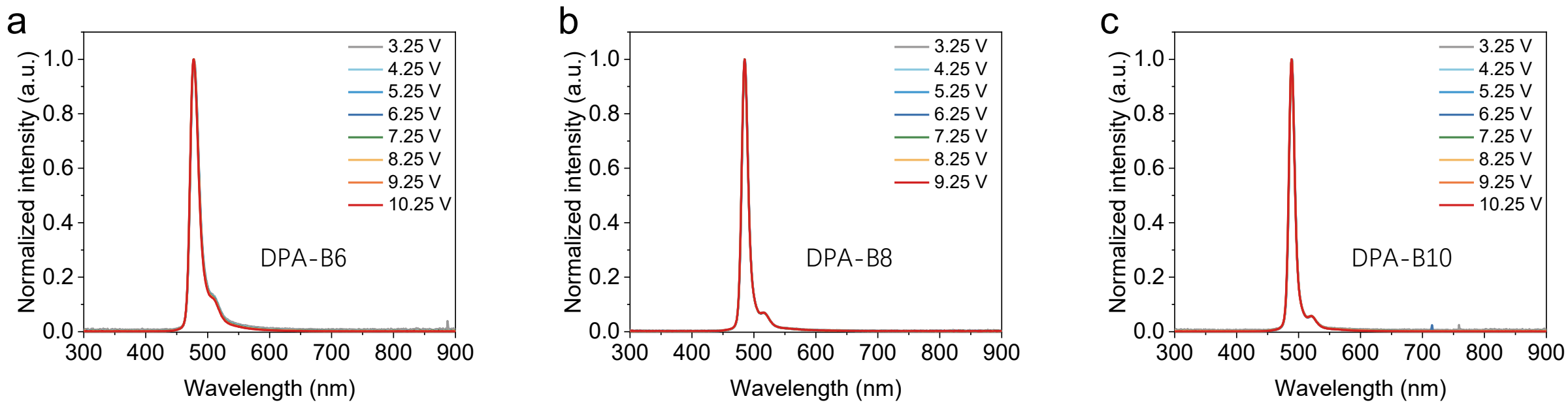

Figure S47. Normalized EL spectra at different operation voltages for DPA-Bn-based devices.

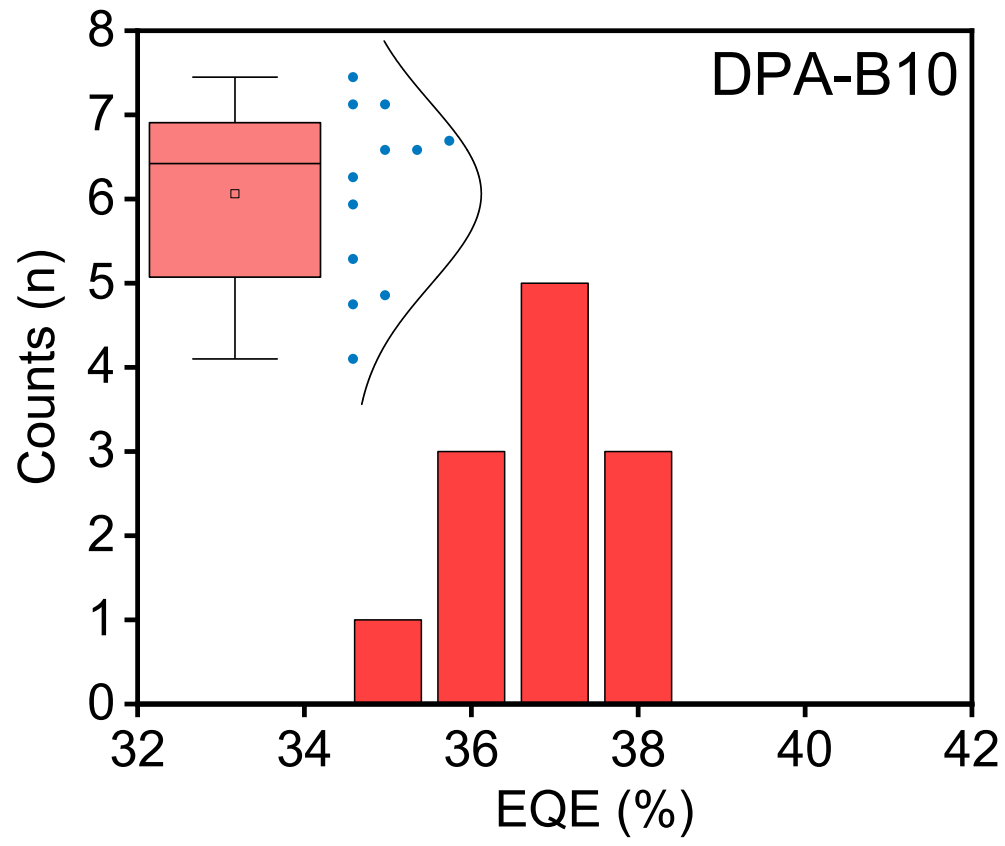

Figure S48. External quantum efficiency histograms for non-sensitized OLEDs with DPA-B10 based on 12 devices (Insert: Box charts showing external quantum efficiency variations, each species based on 12 independent devices).

Table S1. Bond length and bond order parameter between S_0 and S_1 state for **DPA-B6**.

Atoms	Elements	Bond order (S_0)	Bond order (S_1)	Δ BO	Bond length (S_0)	Bond length (S_1)	Δ BL	Δ BL^2
('81', '82')	('C', 'C')	1.24098262	1.20732903	-0.03365359	1.424788	1.432678	0.00789	0.622521
('78', '79')	('C', 'C')	1.24098338	1.20733101	-0.03365237	1.424787	1.432677	0.00789	0.622521
('8', '79')	('N', 'C')	1.15700999	1.18962184	0.03261185	1.377165	1.370973	-0.006192	0.38340864
('22', '81')	('N', 'C')	1.15700981	1.18961483	0.03260502	1.377164	1.370977	-0.006187	0.38278969
('23', '24')	('C', 'C')	1.24367196	1.22088839	-0.02278357	1.42279	1.427807	0.005017	0.25170289
('9', '10')	('C', 'C')	1.2436725	1.22089238	-0.02278012	1.42279	1.427804	0.005014	0.25140196
('82', '83')	('C', 'C')	1.28897569	1.26728039	-0.0216953	1.400334	1.405211	0.004877	0.23785129
('78', '83')	('C', 'C')	1.28897581	1.26728983	-0.02168598	1.400334	1.405208	0.004874	0.23755876
('21', '23')	('N', 'C')	1.16725743	1.18597701	0.01871958	1.375325	1.370833	-0.004492	0.20178064
('7', '9')	('N', 'C')	1.16725726	1.18596856	0.0187113	1.375325	1.370837	-0.004488	0.20142144
('125', '135')	('C', 'C')	1.33862313	1.31279176	-0.02583137	1.416844	1.420405	0.003561	0.12680721
('29', '31')	('C', 'C')	1.33862398	1.31279516	-0.02582882	1.416844	1.420403	0.003559	0.12666481
('17', '18')	('C', 'C')	1.20408376	1.20038787	-0.00369589	1.416696	1.413176	-0.00352	0.123904
('3', '4')	('C', 'C')	1.20408332	1.2003827	-0.00370062	1.416696	1.413177	-0.003519	0.12383361
('3', '8')	('C', 'N')	1.16843106	1.15124259	-0.01718847	1.372219	1.375708	0.003489	0.12173121
('17', '22')	('C', 'N')	1.16843094	1.15124263	-0.01718831	1.372219	1.375707	0.003488	0.12166144
('43', '82')	('B', 'C')	1.1156541	1.13515532	0.01950122	1.543353	1.540093	-0.00326	0.106276
('41', '78')	('B', 'C')	1.11565562	1.13514235	0.01948673	1.543351	1.5401	-0.003251	0.10569001
('30', '75')	('C', 'N')	0.9965159	1.01397626	0.01746036	1.416666	1.413794	-0.002872	0.08248384
('124', '136')	('C', 'N')	0.99651402	1.01395113	0.01743711	1.416666	1.413803	-0.002863	0.08196769
('15', '16')	('C', 'C')	1.29662172	1.27907864	-0.01754308	1.397803	1.400507	0.002704	0.07311616
('1', '2')	('C', 'C')	1.2966213	1.27908911	-	1.397803	1.400505	0.002702	0.07300804

				0.01753219				
('1', '7')	('C', 'N')	1.16271294	1.15095981	-0.01175313	1.372043	1.37468	0.002637	0.06953769
('15', '21')	('C', 'N')	1.16271298	1.1509699	-0.01174308	1.372043	1.374675	0.002632	0.06927424
('30', '40')	('C', 'C')	1.34392735	1.32501583	-0.01891152	1.41614	1.418509	0.002369	0.05612161
('124', '126')	('C', 'C')	1.34392712	1.32502884	-0.01889828	1.416141	1.418503	0.002362	0.05579044
('79', '80')	('C', 'C')	1.31785885	1.30207046	-0.01578839	1.397602	1.399932	0.00233	0.054289
('80', '81')	('C', 'C')	1.3178582	1.30208418	-0.01577402	1.397603	1.399928	0.002325	0.05405625
('18', '43')	('C', 'B')	1.15685995	1.16572759	0.00886764	1.514273	1.516505	0.002232	0.04981824
('4', '41')	('C', 'B')	1.15686037	1.16573392	0.00887355	1.514273	1.516505	0.002232	0.04981824
('29', '75')	('C', 'N')	1.00778427	1.01467369	0.00688942	1.414102	1.416284	0.002182	0.04761124
('125', '136')	('C', 'N')	1.00778327	1.01469556	0.00691229	1.414103	1.416274	0.002171	0.04713241
('13', '45')	('C', 'N')	1.18053523	1.18655525	0.00602002	1.366967	1.364802	-0.002165	0.04687225
('27', '46')	('C', 'N')	1.18053537	1.18655293	0.00601756	1.366967	1.364803	-0.002164	0.04682896
('2', '3')	('C', 'C')	1.29697702	1.29115076	-0.00582626	1.397765	1.399726	0.001961	0.03845521
('16', '17')	('C', 'C')	1.2969763	1.29115435	-0.00582195	1.397765	1.399726	0.001961	0.03845521
('46', '49')	('N', 'C')	1.14661025	1.14129053	-0.00531972	1.378921	1.38074	0.001819	0.03308761
('45', '50')	('N', 'C')	1.14661025	1.14129205	-0.0053182	1.378921	1.380739	0.001818	0.03305124
('26', '27')	('C', 'C')	1.18263092	1.17137284	-0.01125808	1.419553	1.421314	0.001761	0.03101121
('12', '13')	('C', 'C')	1.18263206	1.1713732	-0.01125886	1.419553	1.421313	0.00176	0.030976
('1', '6')	('C', 'C')	1.20916023	1.20987827	0.00071804	1.414577	1.412838	-0.001739	0.03024121
('15', '20')	('C', 'C')	1.20916029	1.20987378	0.00071349	1.414577	1.412839	-0.001738	0.03020644
('34', '35')	('C', 'C')	1.41848579	1.40561701	-0.01286878	1.385147	1.386817	0.00167	0.027889
('131', '132')	('C', 'C')	1.41848429	1.40563233	-0.01285196	1.385147	1.386813	0.001666	0.02775556
('29', '35')	('C', 'C')	1.3375525	1.33895205	0.00139955	1.407261	1.40565	-0.001611	0.02595321
('31', '43')	('C', 'B')	1.08850938	1.10356439	0.01505501	1.548513	1.546905	-0.001608	0.02585664

('41', '135')	('B', 'C')	1.08851003	1.1035739	0.01506387	1.548511	1.546903	-0.001608	0.02585664
('125', '131')	('C', 'C')	1.3375552	1.33893297	0.00137777	1.407261	1.405655	-0.001606	0.02579236
('6', '42')	('C', 'B')	1.14426189	1.14004089	-0.004221	1.518752	1.520356	0.001604	0.02572816
('20', '44')	('C', 'B')	1.14426419	1.1400433	-0.00422089	1.518752	1.520356	0.001604	0.02572816
('5', '6')	('C', 'C')	1.25281687	1.24090781	-0.01190906	1.411489	1.412951	0.001462	0.02137444
('19', '20')	('C', 'C')	1.25281552	1.24091183	-0.01190369	1.411489	1.412948	0.001459	0.02128681
('32', '33')	('C', 'C')	1.42256971	1.41885194	-0.00371777	1.385301	1.386746	0.001445	0.02088025
('133', '134')	('C', 'C')	1.42256906	1.41885813	-0.00371093	1.385301	1.386743	0.001442	0.02079364
('49', '51')	('C', 'C')	1.29545175	1.30376587	0.00831412	1.40771	1.40628	-0.00143	0.020449
('50', '60')	('C', 'C')	1.29545167	1.30376456	0.00831289	1.40771	1.406281	-0.001429	0.02042041
('11', '47')	('C', 'O')	1.1169429	1.10815786	-0.00878504	1.342504	1.3439	0.001396	0.01948816
('25', '48')	('C', 'O')	1.1169394	1.10816691	-0.00877249	1.342503	1.343899	0.001396	0.01948816
('10', '42')	('C', 'B')	1.10937427	1.11218905	0.00281478	1.546465	1.545077	-0.001388	0.01926544
('24', '44')	('C', 'B')	1.1093759	1.11218951	0.00281361	1.546463	1.545079	-0.001384	0.01915456
('51', '52')	('C', 'C')	1.441296	1.426101	-0.015195	1.38315	1.384301	0.001151	0.01324801
('59', '60')	('C', 'C')	1.44129596	1.42610087	-0.01519509	1.38315	1.3843	0.00115	0.013225
('30', '36')	('C', 'C')	1.33972655	1.32926713	-0.01045942	1.407132	1.408258	0.001126	0.01267876
('124', '130')	('C', 'C')	1.33972968	1.32928089	-0.01044879	1.407132	1.408252	0.00112	0.012544
('61', '72')	('C', 'C')	1.31409648	1.30786216	-0.00623432	1.406816	1.407916	0.0011	0.0121
('62', '63')	('C', 'C')	1.31409701	1.30785989	-0.00623712	1.406817	1.407916	0.001099	0.01207801
('38', '39')	('C', 'C')	1.41873887	1.41233144	-0.00640743	1.385322	1.386275	0.000953	0.00908209
('127', '128')	('C', 'C')	1.41873978	1.4123312	-0.00640858	1.385322	1.386275	0.000953	0.00908209
('24', '25')	('C', 'C')	1.22278204	1.22202255	-0.00075949	1.411611	1.412494	0.000883	0.00779689
('10', '11')	('C', 'C')	1.22278237	1.22203269	-	1.411612	1.412489	0.000877	0.00769129

				0.00074968				
('134', '135')	('C', 'C')	1.28721646	1.2806091	-0.00660736	1.410068	1.410877	0.000809	0.00654481
('31', '32')	('C', 'C')	1.28721487	1.28061583	-0.00659904	1.410069	1.410876	0.000807	0.00651249
('37', '38')	('C', 'C')	1.32634703	1.32052447	-0.00582256	1.397913	1.398715	0.000802	0.00643204
('128', '129')	('C', 'C')	1.32634808	1.32053155	-0.00581653	1.397913	1.398714	0.000801	0.00641601
('42', '126')	('B', 'C')	1.08316419	1.0884809	0.00531671	1.553801	1.553012	-0.000789	0.00622521
('40', '44')	('C', 'B')	1.08316753	1.08848152	0.00531399	1.5538	1.553012	-0.000788	0.00620944
('70', '71')	('C', 'C')	1.44165692	1.43589268	-0.00576424	1.389769	1.390495	0.000726	0.00527076
('64', '65')	('C', 'C')	1.44165608	1.43588709	-0.00576899	1.389769	1.390495	0.000726	0.00527076
('53', '54')	('C', 'C')	1.42972159	1.42524476	-0.00447683	1.386116	1.386802	0.000686	0.00470596
('57', '58')	('C', 'C')	1.42972145	1.4252514	-0.00447005	1.386116	1.386802	0.000686	0.00470596
('66', '67')	('C', 'C')	1.41661321	1.40744615	-0.00916706	1.382104	1.38277	0.000666	0.00443556
('68', '69')	('C', 'C')	1.41661399	1.40744704	-0.00916695	1.382104	1.382769	0.000665	0.00442225
('52', '53')	('C', 'C')	1.32815952	1.33317042	0.0050109	1.399905	1.399258	-0.000647	0.00418609
('58', '59')	('C', 'C')	1.32815949	1.33316672	0.00500723	1.399905	1.399258	-0.000647	0.00418609
('5', '136')	('C', 'N')	1.12113973	1.1349812	0.01384147	1.388264	1.387678	-0.000586	0.00343396
('19', '75')	('C', 'N')	1.12114145	1.13497818	0.01383673	1.388264	1.38768	-0.000584	0.00341056
('72', '74')	('C', 'B')	1.08768369	1.09085845	0.00317476	1.55357	1.554133	0.000563	0.00316969
('63', '73')	('C', 'B')	1.08768355	1.09085847	0.00317492	1.55357	1.55413	0.00056	0.003136
('65', '66')	('C', 'C')	1.32162586	1.32482798	0.00320212	1.407419	1.406878	-0.000541	0.00292681
('69', '70')	('C', 'C')	1.32162516	1.32482611	0.00320095	1.40742	1.406879	-0.000541	0.00292681
('39', '40')	('C', 'C')	1.29681741	1.29745548	0.00063807	1.410299	1.409801	-0.000498	0.00248004
('126', '127')	('C', 'C')	1.29681795	1.29745429	0.00063634	1.4103	1.409804	-0.000496	0.00246016
('23', '28')	('C', 'C')	1.27010932	1.26292044	-	1.403452	1.403886	0.000434	0.00188356

				0.00718888				
('9', '14')	('C', 'C')	1.27010916	1.26292995	-0.00717921	1.403452	1.403884	0.000432	0.00186624
('36', '37')	('C', 'C')	1.42010642	1.41985361	-0.00025281	1.384826	1.38442	-0.000406	0.00164836
('129', '130')	('C', 'C')	1.42010725	1.41984394	-0.00026331	1.384826	1.384423	-0.000403	0.00162409
('11', '12')	('C', 'C')	1.25315125	1.24972755	-0.0034237	1.403337	1.402942	-0.000395	0.00156025
('25', '26')	('C', 'C')	1.25315233	1.24973287	-0.00341946	1.403336	1.402942	-0.000394	0.00155236
('48', '62')	('O', 'C')	1.04098686	1.04062736	-0.0003595	1.356507	1.356857	0.00035	0.001225
('47', '61')	('O', 'C')	1.0409897	1.04062608	-0.00036362	1.356507	1.356855	0.000348	0.00121104
('62', '67')	('C', 'C')	1.33457581	1.33884234	0.00426653	1.400757	1.400419	-0.000338	0.00114244
('61', '68')	('C', 'C')	1.33457544	1.33884172	0.00426628	1.400758	1.40042	-0.000338	0.00114244
('63', '64')	('C', 'C')	1.25411422	1.25393408	-0.00018014	1.412105	1.411772	-0.000333	0.00110889
('71', '72')	('C', 'C')	1.25411357	1.25393015	-0.00018342	1.412105	1.411773	-0.000332	0.00110224
('132', '133')	('C', 'C')	1.32916919	1.32587283	-0.00329636	1.398175	1.39843	0.000255	0.00065025
('33', '34')	('C', 'C')	1.32916854	1.32588495	-0.00328359	1.398175	1.398428	0.000253	0.00064009
('50', '56')	('C', 'C')	1.29387679	1.28935105	-0.00452574	1.420183	1.420421	0.000238	0.00056644
('49', '55')	('C', 'C')	1.29387668	1.28935329	-0.00452339	1.420183	1.420421	0.000238	0.00056644
('13', '14')	('C', 'C')	1.33804646	1.33973097	0.00168451	1.392037	1.392252	0.000215	0.00046225
('27', '28')	('C', 'C')	1.33804544	1.33974154	0.0016961	1.392037	1.392248	0.000211	0.00044521
('4', '5')	('C', 'C')	1.25703196	1.23893696	-0.018095	1.411495	1.411673	0.000178	0.00031684
('18', '19')	('C', 'C')	1.2570309	1.23893132	-0.01809958	1.411495	1.411672	0.000177	0.00031329
('54', '55')	('C', 'C')	1.28426715	1.27893658	-0.00533057	1.410237	1.410365	0.000128	0.00016384
('56', '57')	('C', 'C')	1.28426735	1.27892818	-0.00533917	1.410238	1.410366	0.000128	0.00016384
('26', '73')	('C', 'B')	1.15819683	1.16532757	0.00713074	1.520964	1.520859	-0.000105	0.00011025
('12', '74')	('C', 'B')	1.15819661	1.16532839	0.00713178	1.520964	1.520861	-0.000103	0.00010609
('70', '76')	('C', 'C')	1.05255471	1.05273246	0.00017775	1.501067	1.501017	-5E-05	2.5E-05

('65', '77')	('C', 'C')	1.05255469	1.05273246	0.00017777	1.501067	1.501017	-5E-05	2.5E-05
('55', '73')	('C', 'B')	1.10792712	1.11421347	0.00628635	1.553609	1.553584	-2.5E-05	6.25E-06
('56', '74')	('C', 'B')	1.10792712	1.11421845	0.00629133	1.553609	1.553585	-2.4E-05	5.76E-06

Table S2. Bond length and bond order parameter of S_0 and S_1 state for **DPA-B8**.

**Atoms**	**Elements**	**Bond order (S_0)**	**Bond order (S_1)**	**Δ BO**	**Bond length (S_0)**	**Bond length (S_1)**	**Δ BL**	**Δ BL^2**
('22', '61')	('N', 'C')	1.15642625	1.18728903	0.03086278	1.377279	1.370493	-0.006786	0.46049796
('21', '23')	('N', 'C')	1.1564259	1.18728998	0.03086408	1.377279	1.370493	-0.006786	0.46049796
('61', '62')	('C', 'C')	1.24166903	1.21357527	-0.02809376	1.424605	1.431339	0.006734	0.45346756
('23', '24')	('C', 'C')	1.24166856	1.21357404	-0.02809452	1.424606	1.431339	0.006733	0.45333289
('114', '116')	('C', 'C')	1.24130546	1.21669266	-0.0246128	1.4248	1.43046	0.00566	0.320356
('58', '59')	('C', 'C')	1.24130452	1.21669562	-0.0246089	1.4248	1.430459	0.005659	0.32024281
('17', '22')	('C', 'N')	1.16943089	1.15004699	-0.0193839	1.372113	1.376454	0.004341	0.18844281
('15', '21')	('C', 'N')	1.1694297	1.15004827	-0.01938143	1.372113	1.376453	0.00434	0.188356
('116', '119')	('C', 'N')	1.1576129	1.17824269	0.02062979	1.376887	1.372694	-0.004193	0.17581249
('8', '59')	('N', 'C')	1.15761255	1.17824054	0.02062799	1.376887	1.372695	-0.004192	0.17572864
('113', '114')	('C', 'C')	1.28883794	1.26683149	-0.02200645	1.400514	1.404431	0.003917	0.15342889
('58', '63')	('C', 'C')	1.28883786	1.26683246	-0.0220054	1.400515	1.40443	0.003915	0.15327225
('17', '18')	('C', 'C')	1.20264696	1.20242718	-0.00021978	1.417183	1.413877	-0.003306	0.10929636
('15', '20')	('C', 'C')	1.20264806	1.20242531	-0.00022275	1.417182	1.413878	-0.003304	0.10916416
('26', '36')	('C', 'C')	1.34041057	1.31908128	-0.02132929	1.416514	1.419394	0.00288	0.082944
('25', '27')	('C', 'C')	1.34040805	1.31908352	-0.02132453	1.416516	1.419392	0.002876	0.08271376
('114', '115')	('C', 'B')	1.11497448	1.1328101	0.01783562	1.543763	1.540915	-0.002848	0.08111104
('37', '58')	('B', 'C')	1.1149739	1.13280659	0.01783269	1.543764	1.540917	-0.002847	0.08105409
('60', '61')	('C', 'C')	1.31837872	1.29892346	-0.01945526	1.397347	1.400123	0.002776	0.07706176
('23', '120')	('C', 'C')	1.31837944	1.29892411	-0.01945533	1.397347	1.400123	0.002776	0.07706176
('119', '121')	('N', 'C')	1.1681856	1.15560429	-0.01258131	1.372155	1.374878	0.002723	0.07414729
('3', '8')	('C', 'N')	1.16818376	1.15560592	-0.01257784	1.372156	1.374877	0.002721	0.07403841
('144', '154')	('C', 'C')	1.24348692	1.23044305	-0.01304387	1.422927	1.425571	0.002644	0.06990736
('9', '10')	('C', 'C')	1.24348797	1.23044257	-0.0130454	1.422927	1.425571	0.002644	0.06990736
('36', '40')	('C', 'B')	1.0865167	1.10152262	0.01500592	1.548012	1.545378	-0.002634	0.06937956
('27', '39')	('C', 'B')	1.08652058	1.10151958	0.014999	1.548009	1.54538	-0.002629	0.06911641
('140', '144')	('N', 'C')	1.16718261	1.17818783	0.01100522	1.375192	1.372658	-0.002534	0.06421156
('7', '9')	('N', 'C')	1.16718226	1.17818811	0.01100585	1.375192	1.372658	-0.002534	0.06421156
('117', '121')	('C', 'C')	1.20404086	1.20342266	-0.0006182	1.416568	1.414116	-0.002452	0.06012304
('3', '4')	('C', 'C')	1.20404099	1.20341919	-0.0006218	1.416567	1.414118	-0.002449	0.05997601
('19', '56')	('C', 'N')	1.12000734	1.13836613	0.01835879	1.390204	1.3878	-0.002404	0.05779216
('16', '17')	('C', 'C')	1.29590048	1.28431721	-0.01158327	1.397823	1.400057	0.002234	0.04990756
('15', '16')	('C', 'C')	1.29590087	1.28431702	-0.01158385	1.397823	1.400057	0.002234	0.04990756
('127', '133')	('C', 'C')	1.29615527	1.28229751	-0.01385776	1.397635	1.399646	0.002011	0.04044121
('1', '2')	('C', 'C')	1.29615381	1.28229793	-0.01385588	1.397636	1.399647	0.002011	0.04044121
('93', '103')	('C', 'C')	1.33841961	1.32330033	-0.01511928	1.416914	1.418871	0.001957	0.03829849
('118', '123')	('C', 'C')	1.33841783	1.32330242	-0.01511541	1.416914	1.418869	0.001955	0.03822025
('62', '63')	('C', 'C')	1.28885406	1.28319583	-0.00565823	1.400509	1.402407	0.001898	0.03602404
('24', '113')	('C', 'C')	1.28885332	1.28319947	-0.00565385	1.40051	1.402406	0.001896	0.03594816
('18', '19')	('C', 'C')	1.25509329	1.23933749	-0.0157558	1.411565	1.413436	0.001871	0.03500641

('19', '20')	('C', 'C')	1.2550922	1.2393429	-0.0157493	1.411565	1.413434	0.001869	0.03493161
('123', '129')	('C', 'N')	1.00794891	1.01038981	0.0024409	1.414216	1.415945	0.001729	0.02989441
('93', '104')	('C', 'N')	1.00794331	1.01039759	0.00245428	1.414219	1.415941	0.001722	0.02965284
('129', '136')	('N', 'C')	0.9963397	1.0065655	0.0102258	1.416837	1.415219	-0.001618	0.02617924
('92', '104')	('C', 'N')	0.99634299	1.00655748	0.01021449	1.416835	1.415225	-0.00161	0.025921
('4', '37')	('C', 'B')	1.15734822	1.16140592	0.0040577	1.51418	1.515768	0.001588	0.02521744
('115', '117')	('B', 'C')	1.15734992	1.16140644	0.00405652	1.514181	1.515768	0.001587	0.02518569
('1', '6')	('C', 'C')	1.20933352	1.21216019	0.00282667	1.414513	1.413002	-0.001511	0.02283121
('128', '133')	('C', 'C')	1.20933555	1.21215914	0.00282359	1.414513	1.413003	-0.00151	0.022801
('20', '40')	('C', 'B')	1.15762037	1.16228165	0.00466128	1.513798	1.515304	0.001506	0.02268036
('18', '39')	('C', 'B')	1.1576227	1.16228245	0.00465975	1.513798	1.515303	0.001505	0.02265025
('1', '7')	('C', 'N')	1.16312218	1.15684955	-0.00627263	1.371835	1.373223	0.001388	0.01926544
('133', '140')	('C', 'N')	1.1631205	1.15685056	-0.00626994	1.371836	1.373222	0.001386	0.01920996
('136', '141')	('C', 'C')	1.34381081	1.33327265	-0.01053816	1.416142	1.417459	0.001317	0.01734489
('92', '94')	('C', 'C')	1.34380809	1.33327474	-0.01053335	1.416144	1.417458	0.001314	0.01726596
('5', '6')	('C', 'C')	1.25245388	1.24500174	-0.00745214	1.411698	1.412754	0.001056	0.01115136
('122', '128')	('C', 'C')	1.25244946	1.24500368	-0.00744578	1.411699	1.412752	0.001053	0.01108809
('33', '34')	('C', 'C')	1.33061336	1.32311532	-0.00749804	1.397926	1.398966	0.00104	0.010816
('29', '30')	('C', 'C')	1.3306107	1.3231184	-0.0074923	1.397926	1.398965	0.001039	0.01079521
('12', '13')	('C', 'C')	1.18274736	1.17662853	-0.00611883	1.419586	1.420543	0.000957	0.00915849
('156', '158')	('C', 'C')	1.18274325	1.17663216	-0.00611109	1.419587	1.420542	0.000955	0.00912025
('123', '130')	('C', 'C')	1.33730693	1.33899719	0.00169026	1.407312	1.406374	-0.000938	0.00879844
('93', '99')	('C', 'C')	1.33731061	1.3389939	0.00168329	1.407312	1.406376	-0.000936	0.00876096
('130', '137')	('C', 'C')	1.41874869	1.41066142	-0.00808727	1.385183	1.386112	0.000929	0.00863041
('99', '100')	('C', 'C')	1.41874633	1.41066496	-0.00808137	1.385183	1.386111	0.000928	0.00861184
('162', '166')	('N', 'C')	1.14651631	1.14374288	-0.00277343	1.378908	1.379825	0.000917	0.00840889
('37', '103')	('B', 'C')	1.08899089	1.09831867	0.00932778	1.548242	1.547327	-0.000915	0.00837225
('41', '43')	('N', 'C')	1.14651207	1.14374341	-0.00276866	1.37891	1.379824	0.000914	0.00835396
('115', '118')	('B', 'C')	1.08899329	1.09831493	0.00932164	1.54824	1.547328	-0.000912	0.00831744
('27', '28')	('C', 'C')	1.28951959	1.28133508	-0.00818451	1.40989	1.410802	0.000912	0.00831744
('35', '36')	('C', 'C')	1.28952165	1.28133607	-0.00818558	1.409891	1.410802	0.000911	0.00829921
('13', '41')	('C', 'N')	1.1806498	1.18290909	0.00225929	1.366965	1.3661	-0.000865	0.00748225
('158', '162')	('C', 'N')	1.18065389	1.18290799	0.0022541	1.366963	1.366101	-0.000862	0.00743044
('24', '40')	('C', 'B')	1.11598999	1.12452552	0.00853553	1.543053	1.542244	-0.000809	0.00654481
('39', '62')	('B', 'C')	1.11599074	1.12452921	0.00853847	1.543052	1.542243	-0.000809	0.00654481
('128', '135')	('C', 'B')	1.14423559	1.14126307	-0.00297252	1.51882	1.519591	0.000771	0.00594441
('6', '38')	('C', 'B')	1.14423432	1.14126348	-0.00297084	1.51882	1.51959	0.00077	0.005929
('136', '142')	('C', 'C')	1.33972167	1.33326402	-0.00645765	1.407131	1.407818	0.000687	0.00471969
('92', '98')	('C', 'C')	1.33972139	1.33326968	-0.00645171	1.407131	1.407816	0.000685	0.00469225
('124', '131')	('C', 'C')	1.42242176	1.42052865	-0.00189311	1.385239	1.385905	0.000666	0.00443556
('101', '102')	('C', 'C')	1.42242227	1.42052988	-0.00189239	1.385239	1.385904	0.000665	0.00442225
('166', '171')	('C', 'C')	1.29547792	1.29948443	0.00400651	1.407675	1.407029	-0.000646	0.00417316
('43', '48')	('C', 'C')	1.29548061	1.29948392	0.00400331	1.407674	1.407029	-0.000645	0.00416025

('155', '157')	('C', 'O')	1.11673129	1.11276323	-0.00396806	1.342629	1.343265	0.000636	0.00404496
('11', '42')	('C', 'O')	1.11672973	1.11276042	-0.00396931	1.34263	1.343265	0.000635	0.00403225
('116', '120')	('C', 'C')	1.3173821	1.31221782	-0.00516428	1.39763	1.398224	0.000594	0.00352836
('59', '60')	('C', 'C')	1.31738253	1.31221773	-0.0051648	1.39763	1.398224	0.000594	0.00352836
('171', '177')	('C', 'C')	1.44125846	1.43390603	-0.00735243	1.383133	1.383725	0.000592	0.00350464
('47', '48')	('C', 'C')	1.44125624	1.43390689	-0.00734935	1.383135	1.383725	0.00059	0.003481
('10', '38')	('C', 'B')	1.10907779	1.1103939	0.00131611	1.546747	1.54623	-0.000517	0.00267289
('135', '141')	('B', 'C')	1.08330587	1.08598977	0.0026839	1.553549	1.553032	-0.000517	0.00267289
('38', '94')	('B', 'C')	1.08330765	1.0859891	0.00268145	1.553548	1.553033	-0.000515	0.00265225
('135', '154')	('B', 'C')	1.10907963	1.11039126	0.00131163	1.546744	1.546232	-0.000512	0.00262144
('118', '124')	('C', 'C')	1.28750028	1.28342159	-0.00407869	1.410015	1.410524	0.000509	0.00259081
('102', '103')	('C', 'C')	1.28749856	1.28342008	-0.00407848	1.410015	1.410524	0.000509	0.00259081
('144', '161')	('C', 'C')	1.27009345	1.26502578	-0.00506767	1.403359	1.403855	0.000496	0.00246016
('147', '149')	('C', 'C')	1.3264006	1.32271238	-0.00368822	1.397906	1.398402	0.000496	0.00246016
('96', '97')	('C', 'C')	1.3264014	1.32271443	-0.00368697	1.397906	1.398402	0.000496	0.00246016
('9', '14')	('C', 'C')	1.27008977	1.26502531	-0.00506446	1.40336	1.403855	0.000495	0.00245025
('121', '127')	('C', 'C')	1.29722752	1.29668343	-0.00054409	1.397499	1.397938	0.000439	0.00192721
('2', '3')	('C', 'C')	1.29722956	1.29668501	-0.00054455	1.3975	1.397938	0.000438	0.00191844
('122', '129')	('C', 'N')	1.12141861	1.12862649	0.00720788	1.388343	1.387907	-0.000436	0.00190096
('5', '104')	('C', 'N')	1.12141778	1.12862587	0.00720809	1.388342	1.387907	-0.000435	0.00189225
('49', '54')	('C', 'C')	1.31407434	1.31102922	-0.00304512	1.406778	1.4072	0.000422	0.00178084
('160', '164')	('C', 'C')	1.31406877	1.3110294	-0.00303937	1.406779	1.407199	0.00042	0.001764
('30', '31')	('C', 'C')	1.41828109	1.4132536	-0.00502749	1.385403	1.385809	0.000406	0.00164836
('32', '33')	('C', 'C')	1.41828032	1.41325867	-0.00502165	1.385403	1.385808	0.000405	0.00164025
('28', '29')	('C', 'C')	1.42214608	1.42368722	0.00154114	1.385257	1.385638	0.000381	0.00145161
('34', '35')	('C', 'C')	1.42214436	1.42368668	0.00154232	1.385258	1.385637	0.000379	0.00143641
('95', '96')	('C', 'C')	1.41884281	1.41545652	-0.00338629	1.385263	1.385627	0.000364	0.00132496
('146', '149')	('C', 'C')	1.41884441	1.41545778	-0.00338663	1.385263	1.385626	0.000363	0.00131769
('52', '53')	('C', 'C')	1.44153109	1.43848889	-0.0030422	1.389762	1.390098	0.000336	0.00112896
('169', '174')	('C', 'C')	1.44152846	1.43848877	-0.00303969	1.389764	1.390098	0.000334	0.00111556
('168', '172')	('C', 'C')	1.42965198	1.42666372	-0.00298826	1.386136	1.386467	0.000331	0.00109561
('45', '46')	('C', 'C')	1.42963832	1.42666634	-0.00297198	1.386137	1.386467	0.00033	0.001089
('50', '51')	('C', 'C')	1.41670317	1.4124803	-0.00422287	1.382116	1.382434	0.000318	0.00101124
('165', '170')	('C', 'C')	1.41669541	1.41248206	-0.00421335	1.382118	1.382433	0.000315	0.00099225
('142', '147')	('C', 'C')	1.42011542	1.42093108	0.00081566	1.384886	1.384572	-0.000314	0.00098596
('97', '98')	('C', 'C')	1.42011553	1.42092731	0.00081178	1.384886	1.384573	-0.000313	0.00097969
('25', '31')	('C', 'C')	1.33861441	1.33640365	-0.00221076	1.407113	1.406803	-0.00031	0.000961
('46', '47')	('C', 'C')	1.32821772	1.33105724	0.00283952	1.399928	1.399621	-0.000307	0.00094249
('172', '177')	('C', 'C')	1.32821285	1.33105889	0.00284604	1.399928	1.399621	-0.000307	0.00094249
('26', '32')	('C', 'C')	1.3386172	1.33639711	-0.00222009	1.407112	1.406805	-0.000307	0.00094249
('10', '11')	('C', 'C')	1.2230757	1.22332276	0.00024706	1.411679	1.411971	0.000292	0.00085264
('154', '155')	('C', 'C')	1.22307771	1.22332214	0.00024443	1.411679	1.41197	0.000291	0.00084681
('25', '56')	('C', 'N')	1.0036075	1.01268732	0.00907982	1.41649	1.416771	0.000281	0.00078961

('26', '56')	('C', 'N')	1.00360243	1.01269773	0.0090953	1.416492	1.416764	0.000272	0.00073984
('94', '95')	('C', 'C')	1.29692902	1.29759977	0.00067075	1.410248	1.409997	-0.000251	0.00063001
('141', '146')	('C', 'C')	1.29692952	1.29759916	0.00066964	1.410248	1.409998	-0.00025	0.000625
('159', '163')	('B', 'C')	1.10787717	1.11028251	0.00240534	1.553607	1.553382	-0.000225	0.00050625
('44', '55')	('C', 'B')	1.10787374	1.11028323	0.00240949	1.553604	1.553383	-0.000221	0.00048841
('51', '52')	('C', 'C')	1.32167054	1.32309326	0.00142272	1.40742	1.407205	-0.000215	0.00046225
('170', '174')	('C', 'C')	1.32167915	1.3230922	0.00141305	1.407416	1.407206	-0.00021	0.000441
('157', '160')	('O', 'C')	1.04075761	1.04047687	-0.00028074	1.356513	1.356713	0.0002	0.0004
('42', '49')	('O', 'C')	1.04075997	1.04047477	-0.0002852	1.356514	1.356713	0.000199	0.00039601
('117', '122')	('C', 'C')	1.25641973	1.24534272	-0.01107701	1.411695	1.411869	0.000174	0.00030276
('4', '5')	('C', 'C')	1.25641932	1.24534758	-0.01107174	1.411696	1.411868	0.000172	0.00029584
('156', '159')	('C', 'B')	1.15840487	1.1621161	0.00371123	1.520862	1.52069	-0.000172	0.00029584
('12', '55')	('C', 'B')	1.15840187	1.16211742	0.00371555	1.52086	1.52069	-0.00017	0.000289
('49', '50')	('C', 'C')	1.33457474	1.33652737	0.00195263	1.40073	1.400567	-0.000163	0.00026569
('160', '165')	('C', 'C')	1.33458118	1.33652576	0.00194458	1.400729	1.400567	-0.000162	0.00026244
('155', '156')	('C', 'C')	1.25308212	1.25153671	-0.00154541	1.403446	1.403294	-0.000152	0.00023104
('11', '12')	('C', 'C')	1.25308661	1.25153578	-0.00155083	1.403445	1.403294	-0.000151	0.00022801
('53', '54')	('C', 'C')	1.25421188	1.25439758	0.0001857	1.412094	1.411946	-0.000148	0.00021904
('164', '169')	('C', 'C')	1.25421547	1.25439832	0.00018285	1.412092	1.411946	-0.000146	0.00021316
('100', '101')	('C', 'C')	1.32921319	1.32720534	-0.00200785	1.398173	1.398314	0.000141	0.00019881
('131', '137')	('C', 'C')	1.32921148	1.3272085	-0.00200298	1.398174	1.398313	0.000139	0.00019321
('158', '161')	('C', 'C')	1.33805614	1.34044152	0.00238538	1.391935	1.391843	-9.2E-05	8.464E-05
('13', '14')	('C', 'C')	1.33806048	1.34044129	0.00238081	1.391934	1.391842	-9.2E-05	8.464E-05
('54', '55')	('C', 'B')	1.08759474	1.08912348	0.00152874	1.553513	1.553571	5.8E-05	3.364E-05
('159', '164')	('B', 'C')	1.08759345	1.0891243	0.00153085	1.553517	1.553569	5.2E-05	2.704E-05
('52', '57')	('C', 'C')	1.05254666	1.05259707	5.041E-05	1.501045	1.501007	-3.8E-05	1.444E-05
('174', '180')	('C', 'C')	1.0525458	1.05259704	5.124E-05	1.501045	1.501007	-3.8E-05	1.444E-05
('43', '44')	('C', 'C')	1.29392729	1.29230318	-0.00162411	1.420176	1.420188	1.2E-05	1.44E-06
('163', '166')	('C', 'C')	1.2939238	1.29230379	-0.00162001	1.420178	1.420188	1E-05	1E-06
('44', '45')	('C', 'C')	1.28433448	1.28268205	-0.00165243	1.410229	1.410234	5E-06	2.5E-07
('163', '168')	('C', 'C')	1.28432062	1.28268525	-0.00163537	1.41023	1.410233	3E-06	9E-08

Table S3. Bond length and bond order parameter of S_0 and S_1 state for **DPA-B10**.

**Atoms**	**Elements**	**Bond order (S_0)**	**Bond order (S_1)**	**Δ BO**	**Bond length (S_0)**	**Bond length (S_1)**	**Δ BL**	**Δ BL^2**
('114', '116')	('C', 'C')	1.24166646	1.21759735	-0.02406911	1.424693	1.430875	0.006182	0.38217124
('23', '24')	('C', 'C')	1.2416665	1.21759739	-0.02406911	1.424693	1.430875	0.006182	0.38217124
('21', '23')	('N', 'C')	1.15669766	1.18185411	0.02515645	1.377266	1.371689	- 0.005577	0.31102929
('116', '119')	('C', 'N')	1.15669777	1.18184916	0.02515139	1.377266	1.371691	- 0.005575	0.31080625
('22', '61')	('N', 'C')	1.15606439	1.17927716	0.02321277	1.377411	1.372112	- 0.005299	0.28079401
('140', '144')	('N', 'C')	1.15606435	1.17926903	0.02320468	1.377411	1.372117	- 0.005294	0.28026436
('61', '62')	('C', 'C')	1.24155568	1.22091742	-0.02063826	1.424641	1.429307	0.004666	0.21771556
('144', '154')	('C', 'C')	1.2415556	1.22092292	-0.02063268	1.424641	1.429303	0.004662	0.21734244
('58', '59')	('C', 'C')	1.24115126	1.22402162	-0.01712964	1.424823	1.428498	0.003675	0.13505625
('156', '159')	('C', 'C')	1.24115122	1.22402536	-0.01712586	1.424823	1.428493	0.00367	0.134689
('15', '21')	('C', 'N')	1.16906311	1.15187319	-0.01718992	1.372207	1.375705	0.003498	0.12236004
('119', '121')	('N', 'C')	1.16906324	1.15187805	-0.01718519	1.372207	1.375704	0.003497	0.12229009
('17', '22')	('C', 'N')	1.16968865	1.15505887	-0.01462978	1.37207	1.375155	0.003085	0.09517225
('133', '140')	('C', 'N')	1.16968852	1.1550682	-0.01462032	1.37207	1.375151	0.003081	0.09492561
('15', '20')	('C', 'C')	1.20254297	1.20316869	0.00062572	1.417086	1.414056	-0.00303	0.091809
('117', '121')	('C', 'C')	1.20254298	1.20316592	0.00062294	1.417086	1.41406	- 0.003026	0.09156676
('8', '59')	('N', 'C')	1.15758708	1.17030341	0.01271633	1.377018	1.374169	- 0.002849	0.08116801
('159', '162')	('C', 'N')	1.1575874	1.17029662	0.01270922	1.377018	1.374171	- 0.002847	0.08105409
('113', '114')	('C', 'C')	1.28871292	1.27421332	-0.0144996	1.400309	1.403149	0.00284	0.080656
('24', '113')	('C', 'C')	1.2887131	1.27422353	-0.01448957	1.400309	1.403146	0.002837	0.08048569
('17', '18')	('C', 'C')	1.2026876	1.20381626	0.00112866	1.417086	1.41438	- 0.002706	0.07322436
('128', '133')	('C', 'C')	1.20268759	1.20381921	0.00113162	1.417086	1.414381	- 0.002705	0.07317025
('58', '63')	('C', 'C')	1.28924284	1.27365331	-0.01558953	1.400316	1.402996	0.00268	0.071824
('155', '156')	('C', 'C')	1.28924254	1.27366607	-0.01557647	1.400316	1.402994	0.002678	0.07171684
('26', '36')	('C', 'C')	1.34052791	1.32322877	-0.01729914	1.41661	1.418931	0.002321	0.05387041
('118', '123')	('C', 'C')	1.34052789	1.32323337	-0.01729452	1.41661	1.418929	0.002319	0.05377761
('136', '141')	('C', 'C')	1.34063101	1.32482475	-0.01580626	1.416575	1.418736	0.002161	0.04669921
('25', '27')	('C', 'C')	1.34063096	1.32483083	-0.01580013	1.416575	1.418732	0.002157	0.04652649
('19', '56')	('C', 'N')	1.12020315	1.13419909	0.01399594	1.389846	1.387699	- 0.002147	0.04609609
('122', '129')	('C', 'N')	1.12020318	1.13418822	0.01398504	1.389846	1.387708	-	0.04571044

							0.002138	
('144', '163')	('C', 'C')	1.31842409	1.30324952	-0.01517457	1.397457	1.3994	0.001943	0.03775249
('60', '61')	('C', 'C')	1.31842373	1.30324946	-0.01517427	1.397457	1.399398	0.001941	0.03767481
('20', '40')	('C', 'B')	1.15754468	1.16145875	0.00391407	1.513675	1.51554	0.001865	0.03478225
('115', '117')	('B', 'C')	1.15754471	1.16145486	0.00391015	1.513675	1.51554	0.001865	0.03478225
('127', '133')	('C', 'C')	1.29571055	1.28436337	-0.01134718	1.398003	1.399841	0.001838	0.03378244
('16', '17')	('C', 'C')	1.29571034	1.28436826	-0.01134208	1.398003	1.399838	0.001835	0.03367225
('115', '118')	('B', 'C')	1.08642843	1.09976704	0.01333861	1.548398	1.546638	-0.00176	0.030976
('36', '40')	('C', 'B')	1.08642854	1.09977173	0.01334319	1.548398	1.546638	-0.00176	0.030976
('114', '115')	('C', 'B')	1.11575679	1.12945812	0.01370133	1.543113	1.541496	-0.001617	0.02614689
('24', '40')	('C', 'B')	1.11575683	1.12944924	0.01369241	1.543113	1.541498	-0.001615	0.02608225
('27', '39')	('C', 'B')	1.0861789	1.09624173	0.01006283	1.548421	1.546814	-0.001607	0.02582449
('23', '120')	('C', 'C')	1.31782744	1.30469872	-0.01312872	1.397604	1.399209	0.001605	0.02576025
('135', '141')	('B', 'C')	1.08617893	1.09623962	0.01006069	1.548421	1.546817	-0.001604	0.02572816
('116', '120')	('C', 'C')	1.31782715	1.30470482	-0.01312233	1.397604	1.399206	0.001602	0.02566404
('18', '19')	('C', 'C')	1.25530703	1.24420761	-0.01109942	1.411389	1.412987	0.001598	0.02553604
('122', '128')	('C', 'C')	1.25530707	1.24421441	-0.01109266	1.411389	1.412984	0.001595	0.02544025
('3', '8')	('C', 'N')	1.16788078	1.16030255	-0.00757823	1.372325	1.373854	0.001529	0.02337841
('162', '164')	('N', 'C')	1.16788082	1.16030813	-0.00757269	1.372325	1.373852	0.001527	0.02331729
('7', '9')	('N', 'C')	1.16697484	1.17354833	0.00657349	1.375397	1.373871	-0.001526	0.02328676
('183', '187')	('N', 'C')	1.16697501	1.17354378	0.00656877	1.375396	1.373873	-0.001523	0.02319529
('9', '10')	('C', 'C')	1.24369198	1.23600035	-0.00769163	1.422789	1.424265	0.001476	0.02178576
('187', '196')	('C', 'C')	1.24369291	1.23600753	-0.00768538	1.422788	1.424258	0.00147	0.021609
('19', '20')	('C', 'C')	1.25500046	1.24092628	-0.01407418	1.411454	1.412896	0.001442	0.02079364
('117', '122')	('C', 'C')	1.25500032	1.24092559	-0.01407473	1.411454	1.412892	0.001438	0.02067844
('37', '58')	('B', 'C')	1.11506868	1.12706132	0.01199264	1.543517	1.542124	-0.001393	0.01940449
('156', '158')	('C', 'B')	1.11506871	1.12705091	0.0119822	1.543517	1.54213	-0.001387	0.01923769
('3', '4')	('C', 'C')	1.20407489	1.2040499	-2.499E-05	1.416675	1.415344	-0.001331	0.01771561
('160', '164')	('C', 'C')	1.20407496	1.20404778	-2.718E-05	1.416675	1.415347	-0.001328	0.01763584
('15', '16')	('C', 'C')	1.29634908	1.29011622	-0.00623286	1.397888	1.399197	0.001309	0.01713481
('121', '127')	('C', 'C')	1.29634892	1.29012094	-0.00622798	1.397888	1.399194	0.001306	0.01705636
('161', '166')	('C', 'C')	1.33857031	1.32938106	-0.00918925	1.416859	1.418099	0.00124	0.015376
('93', '103')	('C', 'C')	1.33857064	1.32938047	-0.00919017	1.416859	1.418098	0.001239	0.01535121

('123', '129')	('C', 'N')	1.00365765	1.00777308	0.00411543	1.416377	1.417576	0.001199	0.01437601
('26', '56')	('C', 'N')	1.00365763	1.00778193	0.0041243	1.416377	1.417569	0.001192	0.01420864
('170', '176')	('C', 'C')	1.29618739	1.28672527	-0.00946212	1.397878	1.399044	0.001166	0.01359556
('1', '2')	('C', 'C')	1.29618634	1.2867239	-0.00946244	1.397878	1.399044	0.001166	0.01359556
('158', '160')	('B', 'C')	1.15716456	1.15891238	0.00174782	1.514195	1.51536	0.001165	0.01357225
('4', '37')	('C', 'B')	1.15716469	1.15891786	0.00175317	1.514195	1.51536	0.001165	0.01357225
('93', '104')	('C', 'N')	1.0078727	1.00977849	0.00190579	1.414071	1.41521	0.001139	0.01297321
('166', '172')	('C', 'N')	1.00787297	1.00978686	0.00191389	1.414071	1.415204	0.001133	0.01283689
('128', '135')	('C', 'B')	1.15708463	1.15917802	0.00209339	1.513785	1.514792	0.001007	0.01014049
('18', '39')	('C', 'B')	1.15708463	1.15918202	0.00209739	1.513785	1.514792	0.001007	0.01014049
('1', '6')	('C', 'C')	1.2092625	1.21208594	0.00282344	1.414542	1.413561	-0.000981	0.00962361
('171', '176')	('C', 'C')	1.20926339	1.21208824	0.00282485	1.414542	1.413562	-0.00098	0.009604
('154', '155')	('C', 'C')	1.28841447	1.28803912	-0.00037535	1.400349	1.4013	0.000951	0.00904401
('62', '63')	('C', 'C')	1.28841425	1.28804215	-0.0003721	1.400349	1.401298	0.000949	0.00900601
('118', '124')	('C', 'C')	1.28919487	1.28132031	-0.00787456	1.409965	1.410906	0.000941	0.00885481
('35', '36')	('C', 'C')	1.28919496	1.2813205	-0.00787446	1.409964	1.410905	0.000941	0.00885481
('29', '30')	('C', 'C')	1.33062656	1.32377517	-0.00685139	1.397901	1.398762	0.000861	0.00741321
('147', '149')	('C', 'C')	1.33062658	1.32377607	-0.00685051	1.397901	1.398761	0.00086	0.007396
('5', '6')	('C', 'C')	1.25301768	1.24775777	-0.00525991	1.411455	1.412246	0.000791	0.00625681
('165', '171')	('C', 'C')	1.25301873	1.24776099	-0.00525774	1.411455	1.412243	0.000788	0.00620944
('172', '179')	('N', 'C')	0.99633662	1.00202557	0.00568895	1.416721	1.41596	-0.000761	0.00579121
('92', '104')	('C', 'N')	0.99633686	1.00202513	0.00568827	1.416721	1.415961	-0.00076	0.005776
('92', '94')	('C', 'C')	1.34394736	1.33799761	-0.00594975	1.416136	1.416861	0.000725	0.00525625
('179', '184')	('C', 'C')	1.34394783	1.33799915	-0.00594868	1.416136	1.416859	0.000723	0.00522729
('123', '130')	('C', 'C')	1.33860047	1.33965323	0.00105276	1.407094	1.406378	-0.000716	0.00512656
('26', '32')	('C', 'C')	1.3386005	1.33964882	0.00104832	1.407094	1.40638	-0.000714	0.00509796
('176', '183')	('C', 'N')	1.16304164	1.15997112	-0.00307052	1.371953	1.372629	0.000676	0.00456976
('1', '7')	('C', 'N')	1.16304321	1.15997173	-0.00307148	1.371952	1.372627	0.000675	0.00455625
('33', '34')	('C', 'C')	1.33056162	1.3254384	-0.00512322	1.397904	1.398563	0.000659	0.00434281
('131', '137')	('C', 'C')	1.33056159	1.32543997	-0.00512162	1.397904	1.398563	0.000659	0.00434281
('129', '136')	('N', 'C')	1.00334596	1.01257212	0.00922616	1.416438	1.415831	-0.000607	0.00368449
('27', '28')	('C', 'C')	1.28933716	1.28367916	-0.005658	1.409957	1.410564	0.000607	0.00368449
('141', '146')	('C', 'C')	1.28933716	1.28368879	-0.00564837	1.409957	1.410563	0.000606	0.00367236
('25', '56')	('C', 'N')	1.003346	1.01255458	0.00920858	1.416438	1.415845	-0.000593	0.00351649
('198', '200')	('C', 'C')	1.18259519	1.17896535	-0.00362984	1.419562	1.420128	0.000566	0.00320356
('12', '13')	('C', 'C')	1.18260164	1.17898658	-0.00361506	1.419561	1.420123	0.000562	0.00315844
('130', '137')	('C', 'C')	1.41829252	1.4115818	-0.00671072	1.385357	1.385897	0.00054	0.002916

('32', '33')	('C', 'C')	1.41829245	1.41158484	-0.00670761	1.385357	1.385897	0.00054	0.002916
('6', '38')	('C', 'B')	1.14391174	1.14185201	-0.00205973	1.518823	1.519359	0.000536	0.00287296
('171', '178')	('C', 'B')	1.14391085	1.14185069	-0.00206016	1.518822	1.519355	0.000533	0.00284089
('200', '204')	('C', 'N')	1.18075073	1.18191855	0.00116782	1.366909	1.366409	-0.0005	0.0025
('13', '41')	('C', 'N')	1.18074587	1.18190296	0.00115709	1.366912	1.366418	-0.000494	0.00244036
('93', '99')	('C', 'C')	1.33758406	1.33799199	0.00040793	1.407261	1.406787	-0.000474	0.00224676
('166', '173')	('C', 'C')	1.33758388	1.33798703	0.00040315	1.407261	1.40679	-0.000471	0.00221841
('158', '161')	('B', 'C')	1.08876772	1.09422036	0.00545264	1.548417	1.547956	-0.000461	0.00212521
('37', '103')	('B', 'C')	1.08876771	1.09422165	0.00545394	1.548417	1.547957	-0.00046	0.002116
('99', '100')	('C', 'C')	1.4185324	1.41414128	-0.00439112	1.385143	1.385582	0.000439	0.00192721
('173', '180')	('C', 'C')	1.41853245	1.41414779	-0.00438466	1.385143	1.385581	0.000438	0.00191844
('41', '43')	('N', 'C')	1.14650303	1.14511938	-0.00138365	1.378933	1.379356	0.000423	0.00178929
('204', '208')	('N', 'C')	1.14650817	1.14513473	-0.00137344	1.37893	1.379348	0.000418	0.00174724
('11', '42')	('C', 'O')	1.11708172	1.11515094	-0.00193078	1.342475	1.342856	0.000381	0.00145161
('197', '199')	('C', 'O')	1.11709085	1.1151846	-0.00190625	1.342472	1.342846	0.000374	0.00139876
('124', '131')	('C', 'C')	1.42220726	1.42384734	0.00164008	1.385325	1.385695	0.00037	0.001369
('34', '35')	('C', 'C')	1.42220721	1.4238474	0.00164019	1.385325	1.385695	0.00037	0.001369
('5', '104')	('C', 'N')	1.12130213	1.12568471	0.00438258	1.388214	1.387845	-0.000369	0.00136161
('165', '172')	('C', 'N')	1.12130154	1.12567318	0.00437164	1.388215	1.387854	-0.000361	0.00130321
('92', '98')	('C', 'C')	1.33973085	1.33587227	-0.00385858	1.407128	1.407482	0.000354	0.00125316
('179', '185')	('C', 'C')	1.33973085	1.33587062	-0.00386023	1.407128	1.40748	0.000352	0.00123904
('102', '103')	('C', 'C')	1.28711192	1.28475266	-0.00235926	1.410076	1.410411	0.000335	0.00112225
('161', '167')	('C', 'C')	1.28711198	1.28475746	-0.00235452	1.410076	1.41041	0.000334	0.00111556
('43', '48')	('C', 'C')	1.29546308	1.29742012	0.00195704	1.407699	1.407386	-0.000313	0.00097969
('208', '213')	('C', 'C')	1.29545934	1.29740838	0.00194904	1.4077	1.40739	-0.00031	0.000961
('101', '102')	('C', 'C')	1.4226113	1.42139061	-0.00122069	1.385286	1.385595	0.000309	0.00095481
('167', '174')	('C', 'C')	1.4226113	1.42138813	-0.00122317	1.385286	1.385595	0.000309	0.00095481
('10', '11')	('C', 'C')	1.22255325	1.22225471	-0.00029854	1.411665	1.411964	0.000299	0.00089401
('196', '197')	('C', 'C')	1.22255204	1.22224687	-0.00030517	1.411665	1.411963	0.000298	0.00088804
('190', '192')	('C', 'C')	1.32641828	1.32445486	-0.00196342	1.397905	1.398191	0.000286	0.00081796
('96', '97')	('C', 'C')	1.32641808	1.32445284	-0.00196524	1.397905	1.398191	0.000286	0.00081796
('47', '48')	('C', 'C')	1.44129266	1.43761067	-0.00368199	1.383139	1.383416	0.000277	0.00076729
('213', '219')	('C', 'C')	1.44129644	1.43762315	-0.00367329	1.383137	1.38341	0.000273	0.00074529
('28', '29')	('C', 'C')	1.4221565	1.42369068	0.00153418	1.385326	1.385571	0.000245	0.00060025
('146', '149')	('C', 'C')	1.42215641	1.4236831	0.00152669	1.385326	1.385571	0.000245	0.00060025
('9', '14')	('C', 'C')	1.27030845	1.26713384	-0.00317461	1.403395	1.403633	0.000238	0.00056644

('178', '184')	('B', 'C')	1.08316872	1.08417737	0.00100865	1.553781	1.553546	- 0.000235	0.00055225
('38', '94')	('B', 'C')	1.08317	1.08418226	0.00101226	1.553782	1.553548	- 0.000234	0.00054756
('187', '203')	('C', 'C')	1.27031145	1.26714477	-0.00316668	1.403394	1.403627	0.000233	0.00054289
('136', '142')	('C', 'C')	1.33870151	1.33478694	-0.00391457	1.407062	1.407276	0.000214	0.00045796
('25', '31')	('C', 'C')	1.33870152	1.33479467	-0.00390685	1.407062	1.407274	0.000212	0.00044944
('202', '206')	('C', 'C')	1.31410028	1.31235197	-0.00174831	1.406824	1.407033	0.000209	0.00043681
('189', '192')	('C', 'C')	1.41865994	1.41652869	-0.00213125	1.385326	1.385534	0.000208	0.00043264
('49', '54')	('C', 'C')	1.31410093	1.31235194	-0.00174899	1.406822	1.407029	0.000207	0.00042849
('95', '96')	('C', 'C')	1.41865997	1.41653172	-0.00212825	1.385326	1.385533	0.000207	0.00042849
('97', '98')	('C', 'C')	1.42006856	1.4205046	0.00043604	1.384827	1.384623	- 0.000204	0.00041616
('185', '190')	('C', 'C')	1.42006811	1.42050531	0.0004372	1.384827	1.384625	- 0.000202	0.00040804
('42', '49')	('O', 'C')	1.0408873	1.04070889	-0.00017841	1.356531	1.356729	0.000198	0.00039204
('46', '47')	('C', 'C')	1.32815052	1.32959953	0.00144901	1.399902	1.399707	- 0.000195	0.00038025
('214', '219')	('C', 'C')	1.32814434	1.32958099	0.00143665	1.399901	1.399707	- 0.000194	0.00037636
('199', '202')	('O', 'C')	1.04089276	1.04072948	-0.00016328	1.35653	1.356722	0.000192	0.00036864
('159', '163')	('C', 'C')	1.31727299	1.3166171	-0.00065589	1.397731	1.397544	- 0.000187	0.00034969
('59', '60')	('C', 'C')	1.31727327	1.31661113	-0.00066214	1.397731	1.397546	- 0.000185	0.00034225
('50', '51')	('C', 'C')	1.41663088	1.41455083	-0.00208005	1.382098	1.382274	0.000176	0.00030976
('52', '53')	('C', 'C')	1.44174829	1.4402837	-0.00146459	1.389769	1.389944	0.000175	0.00030625
('13', '14')	('C', 'C')	1.3378391	1.33972426	0.00188516	1.392057	1.391882	- 0.000175	0.00030625
('207', '212')	('C', 'C')	1.41663037	1.41455319	-0.00207718	1.382096	1.382269	0.000173	0.00029929
('211', '216')	('C', 'C')	1.44176283	1.44033653	-0.0014263	1.389769	1.389941	0.000172	0.00029584
('200', '203')	('C', 'C')	1.33783649	1.33971404	0.00187755	1.392058	1.391887	- 0.000171	0.00029241
('178', '196')	('B', 'C')	1.10960786	1.11051898	0.00091112	1.546411	1.546256	- 0.000155	0.00024025
('10', '38')	('C', 'B')	1.10960669	1.1105125	0.00090581	1.546412	1.546265	- 0.000147	0.00021609
('212', '216')	('C', 'C')	1.32160019	1.32225507	0.00065488	1.40742	1.407274	- 0.000146	0.00021316
('51', '52')	('C', 'C')	1.32160245	1.32226885	0.0006664	1.407421	1.407276	- 0.000145	0.00021025
('142', '147')	('C', 'C')	1.41817847	1.41705324	-0.00112523	1.385379	1.385242	- 0.000137	0.00018769

('30', '31')	('C', 'C')	1.41817848	1.41704719	-0.00113129	1.385379	1.385242	- 0.000137	0.00018769
('45', '46')	('C', 'C')	1.42984192	1.42829823	-0.00154369	1.386108	1.386235	0.000127	0.00016129
('210', '214')	('C', 'C')	1.42985996	1.42835343	-0.00150653	1.386107	1.386232	0.000125	0.00015625
('198', '201')	('C', 'B')	1.15810788	1.15972014	0.00161226	1.521015	1.52114	0.000125	0.00015625
('4', '5')	('C', 'C')	1.25680122	1.25012733	-0.00667389	1.411543	1.411668	0.000125	0.00015625
('160', '165')	('C', 'C')	1.25680113	1.2501375	-0.00666363	1.411543	1.411663	0.00012	0.000144
('44', '55')	('C', 'B')	1.10794504	1.10903681	0.00109177	1.553638	1.553524	- 0.000114	0.00012996
('12', '55')	('C', 'B')	1.1581049	1.1597161	0.0016112	1.52101	1.521122	0.000112	0.00012544
('184', '189')	('C', 'C')	1.29685154	1.29770774	0.0008562	1.4103	1.410194	- 0.000106	0.00011236
('2', '3')	('C', 'C')	1.29743363	1.29785844	0.00042481	1.397676	1.39757	- 0.000106	0.00011236
('164', '170')	('C', 'C')	1.29743261	1.29785781	0.0004252	1.397676	1.39757	- 0.000106	0.00011236
('94', '95')	('C', 'C')	1.29685209	1.29770735	0.00085526	1.4103	1.410195	- 0.000105	0.00011025
('201', '205')	('B', 'C')	1.10794806	1.10904589	0.00109783	1.553646	1.553547	-9.9E-05	9.801E-05
('54', '55')	('C', 'B')	1.08769738	1.08871028	0.0010129	1.553597	1.553498	-9.9E-05	9.801E-05
('201', '206')	('B', 'C')	1.08769766	1.08871124	0.00101358	1.553606	1.553525	-8.1E-05	6.561E-05
('53', '54')	('C', 'C')	1.25403217	1.2540289	-3.27E-06	1.412107	1.412031	-7.6E-05	5.776E-05
('206', '211')	('C', 'C')	1.25401941	1.25398073	-3.868E-05	1.412108	1.412036	-7.2E-05	5.184E-05
('205', '210')	('C', 'C')	1.28414915	1.28327905	-0.0008701	1.410247	1.410317	7E-05	4.9E-05
('11', '12')	('C', 'C')	1.25324282	1.25287807	-0.00036475	1.40333	1.403261	-6.9E-05	4.761E-05
('197', '198')	('C', 'C')	1.25323606	1.25286319	-0.00037287	1.403332	1.403264	-6.8E-05	4.624E-05
('44', '45')	('C', 'C')	1.28416684	1.28333268	-0.00083416	1.410245	1.410313	6.8E-05	4.624E-05
('135', '154')	('B', 'C')	1.11632448	1.12000256	0.00367808	1.542936	1.543	6.4E-05	4.096E-05
('39', '62')	('B', 'C')	1.1163246	1.12000847	0.00368387	1.542936	1.542998	6.2E-05	3.844E-05
('49', '50')	('C', 'C')	1.33455018	1.33544395	0.00089377	1.400756	1.400702	-5.4E-05	2.916E-05
('174', '180')	('C', 'C')	1.32919197	1.32773706	-0.00145491	1.398185	1.398238	5.3E-05	2.809E-05
('43', '44')	('C', 'C')	1.29389319	1.29322793	-0.00066526	1.420183	1.42013	-5.3E-05	2.809E-05
('202', '207')	('C', 'C')	1.33454883	1.33543679	0.00088796	1.400757	1.400705	-5.2E-05	2.704E-05
('100', '101')	('C', 'C')	1.3291919	1.3277391	-0.0014528	1.398185	1.398237	5.2E-05	2.704E-05
('205', '208')	('C', 'C')	1.29388952	1.29321687	-0.00067265	1.420184	1.420135	-4.9E-05	2.401E-05
('216', '222')	('C', 'C')	1.05255665	1.05260643	4.978E-05	1.501061	1.501012	-4.9E-05	2.401E-05
('52', '57')	('C', 'C')	1.05255656	1.05260282	4.626E-05	1.501061	1.501013	-4.8E-05	2.304E-05

Table S4. Physical data of DPA-B6, DPA-B8, and DPA-B10.

Emitter	λ_{abs}[a] [nm]	λ_{em}[b] [nm]	FWHM[c] [nm/meV]	E_{HOMO}[d] [eV]	E_{LUMO}[d] [eV]	Φ_{PL}[e] (%)	τ_{PF}[f] [ns]	τ_{DF}[f] [ns]
**DPA-B6**	467	472	11.4 (67)	-5.09	-2.48	98	2.44	181.2
**DPA-B8**	475	479	10.4 (55)	-5.06	-2.50	99	2.66	143.0
**DPA-B10**	478	482	9.1 (49)	-5.05	-2.51	100	2.78	136.4

[a]Maximum absorption wavelength measured in dilute toluene. [b]Maximum emission wavelength measured in toluene. [c]Full-width at half-maximum of the PL spectra. [d]HOMO and LUMO energies recorded in dichloromethane solution. [e]Photoluminescence quantum yields measured after nitrogen bubbling into toluene solution. Emission lifetime for prompt (τ_{PF}) and delayed fluorescence (τ_{DF}) obtained from time-resolved photoluminescence spectra.

Table S5. EL performance of the MR-TADF emitters-based solution-processed devices.

Emitters	Doped ratio (wt%)	EL (nm)	FWHM (nm)	EQE_{max} (%)	EQE_{1000} (%)	References
TriCzBN	2	491	27	17.8	2.9	*Chem. Eng. J.* **493,** 152857 (2024)
TriCzBN (HF)	2	496	30	28.8	26.1	
FlCzBN-BO	2	504	31	16.3	10.35/500 cd m^{-2}	*Chem. Eng. J.* **506,** 160139 (2025)
BNB′-1 (HF)	1.6	539	30	36.2	10.4	*Adv. Funct. Mater.* **33,** 2306394 (2023)
PCzTBN5	30	511	44	22.2	4.0	*Adv. Funct. Mater.* **34**, 2408119 (2024)
OAB-ABP-1	2	505	33	21.8	17.4	*Adv. Mater.* **32***,* 2004072 (2020)
PSi3	30	506	42	28.8	2.2	*Adv. Mater.* **37***,* 2502180 (2025)
PSi3 (HF)	30	506	44	30.2	3.3	
2GtBuCzCO2HDCzB	30	495	27	27.9	27.6 (2000)	*Adv. Mater.* **37,** 2415289 (2025)
4FlCzBN	16	500	30	10.9	4.6	*Adv. Optical Mater.* **12,** 2301217 (2024)
4FlCzBN (HF)	4	496	30	12.2	11.0	
3CzSF-BN	2	522	36	20.3	4.8	*Adv. Optical Mater.* **12,** 2400490 (2024)
3CzSF-BN (HF)	2	519	37	23.1	21.3	
D1-DBN	0.6	471	18	26.3	21.4	*Angew. Chem. Int. Ed.* **64,** e202423812 (2025)
D1-DBN (HF)	0.6	472	18	30.2	20.4	
D2-DBN	0.6	471	16	28.3	23.0	
D2-DBN (HF)	0.6	471	17	35.3	29.5	
PCzBN1	60	491	27	17.8	/	*Angew. Chem. Int. Ed.* **134,** e202211172 (2022)
DiKTa-LC	10	492	51	13.6	/	*Angew. Chem. Int. Ed.* **135,** e202218911 (2023)
BN-36Cz-BN	5	495	31	27.1	2.9	*Angew. Chem. Int. Ed.* **135,** e202301988 (2023)
BN-R	1	617	47	22	7.1	*Angew. Chem. Int. Ed.* **135,** e202216473 (2022)
BN-R (HF)	1	618	47	20.1	13.3	
P10	10	505	42	19.4	/	*Chem. Commun.* **60,** 10318－10321 (2024)
Cy-tmCPBN	4	496	36	12.3	/	*Chem. Sci.* **15,** 12361－12368 (2024)
tBuCzBN-PXZ	1	480	27	21.3	/	*Chem. Sci.* **15,** 18335－18346 (2024)
tBuCzBN-Ph-PSeZ	1	496	36	19.4	/	
m-DT-BCzBN	2	492	29	27.8	21.5（500）	*Chem. Sci.* **16,** 3904－3915 (2025)
p-DT-BCzBN	2	494	29	25.4	11.0	
DOBN	1	449	20	6.75	/	*ACS Materials Lett.* **6,** 3246－3253 (2024)
DOBN (HF)	1	447	20	27.7	/	

PCzPBN5	20	462	31	10.1	/	*Macromolecules* **58,** 363－371 (2025)
6TBN	20	496	25	23	6.4	*ACS Appl. Mater. Interfaces* **15,** 39669−39676 (2023)
t-BuCz-DABNA (HF)	1.5	472	16.6	29.2	26.0	*Angew. Chem. Int. Ed.* **135,** e202313084 (2023)
tBO-4B	1	452	16	30.3	/	*Adv. Mater.* **37,** 2500010 (2025)
Cz-pDBIC(HF)	1.5	520	34	40.6	30.6	*Adv. Mater.* **38**, e13676 (2025)
C^{+}NiPr-BPhF20	2	558	44	29.4	/	*Angew. Chem. Int. Ed.* **64**, e202510511(2025)
NCON-Mes	2	524	39	27.7	13.8	*Chem. Eng. J.* **507**, 160102 (2025)
BNCZ-DPAB	2	624	52	27.7	/	*Sci. Bull.* **69**, 2983-2986 (2024)
Endo-D2	10	472	38	22.6	/	*Angew. Chem. Int. Ed.* **64**, e202415607 (2025)
BN-CP1	1	492	28	26.6	/	*Adv. Mater.* **35**, 2300510 (2023)
V-DABNA-Mes	1	480	27	22.9	10.9	*J. Am. Chem. Soc.* **144**, 106-112 (2022)
m-Cz-BNCz	5	519	42	24.2	17.7	*Chem. Eng. J.* **447**, 137517 (2022)
TCzBN-SO		516	41	25.5	/	*Chem. Eng. J.* 465, 142900 (2023)
DtCzBN-CNBT2	15	507	39	24.4	5.6	*Adv. Mater.* **36**, 2412761 (2024,)
BNCz-4S (HF)	1	496	43	24.1	8.66	*Adv. Optical Mater.* **13**, 2403566 (2025)
BN-R2	1	601	40	21.1	9.9	*Adv. Optical Mater.* **12**, 2302811 (2024)
tBuCzBN-PXZ	1	480	27	21.3	/	*Chem. Sci.* **15**, 18335-18346 (2024)
PCzDBN3	70	479	39	17.9	4.0	*Adv. Funct. Mater.* **34**, 2310042 (2024)
BN-N-TTz	1	490	28	25.9	/	*Chem. Sci.* **16**, 19956-19966 (2025)
2,7-tBuCzNB	5	500	41	11.4	/	*Org. Chem. Front.,* **12**, 3279-3287 (2025)
DPA-B6	0.6	474	16.2	33.0	20.0	*This work*
DPA-B8	0.6	479	12.0	35	23.0	
DPA-B10	0.6	484	10.9	38.1	22.7	

Table S6. EL performance of the representation solution-processed devices based on TADF emitters.

**Emitters**	**Doped ratio (wt%)**	**EL (nm)**	**EQE$_{max}$ (%)**	**EQE$_{1000}$ (%)**	**References**
5CzBN-12CSP	100	508	17.6	/	*Org. Electron.* **128,** 107043 (2024)
5CzBN-9CSP	100	508	21.1	/	
TBP-DMAc	10	522	22.1	20.7	*Adv. Funct. Mater.* **28,** 1704927 (2018)
PCzAQC0.5	100	620	12.5	/	*Adv. Funct. Mater.* **30,** 2002493 (2020)
NAI_R3	1.5	620	22.5	3.4	*Adv. Mater.* **31,** 1901404 (2019)
2tDMG	10	504	20.1	5.1	*Adv. Mater.* **32,** 2003885 (2020)
3tDMG	10	486	16.2	6.4	
MTPA-BPSB	7	527	21	/	*Adv. Optical Mater.* **8,** 1901021 (2020)
SAQF10	40	608	24.8	/	*Adv. Optical Mater.* **9,** *2001981* (2021)
BNCz-4S	1	496	24.1	/	*Adv. Optical Mater.* **23,** 2403566 (2025)
BNCz-2S	1	492	19.4	/	
T-CNDF-T-*t*Cz	100	484	21.0	/	*Adv. Sci.* **7,** 1902087 (2020)
oDTBPZ-DPXZ	8	612	18.5	/	*Angew. Chem. Int. Ed.* **60,** 2478 – 2484 (2021)
poly(TMTPA – DCB)	10	532	24.0	18.2	*Angew. Chem. Int. Ed.* **60,** 9635 – 9641 (2021)
YD – TF	3	552	21.9	18.6	*Angew. Chem. Int. Ed.* **60,** 16585 – 16593 (2021)
5CzBN-ESF	100	508	30.6	/	*Angew. Chem. Int. Ed.* **61,** e202212861 (2022)
QX36	30	602	32.3	20.6	*Angew. Chem. Int. Ed.* **64,** e202501179 (2025)
QX27	30	596	24.9	11.7	
DCz-DPS-TCz	100	500	24.0	/	*Angew. Chem. Int. Ed.* **134,** e202115140 (2022)
DCz-DPS-Cz	100	498	23.3	/	
DDPhCz-DCPP	20	578	20.6	/	*J. Mater. Chem. C* **7,** 12321—12327 (2019)
ND37DBT	2	606	22.1	/	*Chem. Eur. J.* **31,** e202501391 (2025)
ND28DBT	2	608	19.0	/	
ICz-TDBA	20	431	13.0	11.8	*J. Mater. Chem. C* **11,** 8767 – 8775 (2023)
p-2PXZ-XN	90	568	25.6	23.1	*Small* **21,** 2502892 (2025)
Cz-pDBIC	1.5	520	40.6	30.6	*Adv. Mater.* **38**, e13676 (2026)
DPA-B6	0.6	477	33.0	19.9	*This work*
DPA-B8	0.6	485	35.0	21.9	
DPA-B10	0.6	489	38.1	24.4	

Table S7. Cartesian coordinates of **DPA-B6** at the optimized S_0 geometry.

C	-6.02956	1.92937	-0.26694	B	12.63057	1.34035	-0.18279
C	-4.74833	2.4787	-0.16439	B	-12.63057	1.34035	0.18278
C	-3.65586	1.61242	-0.06535	N	5.30545	-1.68682	0.23309
C	-3.82008	0.20558	-0.0358	C	-15.54977	-2.85665	1.05787
C	-5.12357	-0.31096	-0.19827	C	15.54979	-2.85665	-1.05775
C	-6.2477	0.53297	-0.32627	C	-1.23991	-0.02191	0.06915
N	-7.12239	2.75735	-0.31848	C	-1.21202	1.40189	0.02397
N	-2.38473	2.12379	0.0101	C	-0.00001	2.09742	0.00008
C	-8.44277	2.37618	-0.26544	C	1.212	1.40189	-0.02383
C	-8.7943	0.9979	-0.29907	C	1.2399	-0.0219	-0.06903
C	-10.16682	0.73044	-0.10594	C	-0.00001	-0.66906	0.00004
C	-11.16595	1.70969	0.00438	H	-4.60503	3.56286	-0.15364
C	-10.74896	3.0652	-0.05739	H	-6.94181	3.75169	-0.28479
C	-9.40166	3.39656	-0.17034	H	-2.29706	3.13102	-0.01446
C	6.02956	1.92937	0.26694	H	-9.08745	4.44413	-0.17093
C	4.74832	2.4787	0.16443	H	4.60502	3.56286	0.1537
C	3.65585	1.61242	0.06543	H	6.94181	3.75169	0.28478
C	3.82007	0.20559	0.03586	H	2.29705	3.13102	0.01459
C	5.12356	-0.31096	0.19828	H	9.08744	4.44414	0.17088
C	6.2477	0.53297	0.32625	H	1.09773	-2.76208	-1.3283
N	7.12238	2.75736	0.31845	H	1.86009	-4.95826	-2.14931
N	2.38472	2.12379	-0.00998	H	4.27947	-5.57093	-1.9448
C	8.44277	2.37619	0.26539	H	5.85757	-4.03326	-0.8564
C	8.79429	0.99791	0.299	H	5.33579	-3.97572	1.55271
C	10.16682	0.73045	0.1059	H	7.21403	-4.94153	2.81061
C	11.16595	1.7097	-0.00441	H	9.39353	-3.71294	2.95366
C	10.74896	3.06521	0.05734	H	9.61584	-1.51426	1.85716
C	9.40166	3.39657	0.17028	H	-11.38712	5.00027	-0.01575
C	4.35914	-2.54575	-0.37223	H	11.38711	5.00028	0.0157
C	6.43045	-2.22144	0.908	H	13.43577	5.97915	-0.07252
C	3.00484	-2.15673	-0.52056	H	15.89593	5.74302	-0.17094
C	2.13548	-3.05894	-1.16752	H	16.93202	3.46638	-0.22861
C	2.5609	-4.286	-1.64957	H	15.49789	1.47502	-0.21833
C	3.91033	-4.63093	-1.52728	H	-15.49789	1.47501	0.21835
C	4.80106	-3.7697	-0.90799	H	-16.93202	3.46637	0.22864
C	6.29195	-3.45272	1.57493	H	-15.89593	5.74301	0.17093
C	7.34889	-3.98992	2.29051	H	-13.43577	5.97914	0.07248
C	8.56232	-3.30122	2.37673	H	14.99847	-0.22939	-0.77149
C	8.68613	-2.07142	1.75113	H	13.13933	-4.0845	-0.69024
C	7.64177	-1.49363	0.99984	H	10.93468	-3.00801	-0.23056
B	-2.6227	-0.70248	0.15057	H	-10.93467	-3.00802	0.23062
B	-7.65045	-0.02428	-0.49473	H	-13.13931	-4.0845	0.69035

B	2.62268	-0.70247	-0.15047	H	-14.99846	-0.22939	0.77155
B	7.65045	-0.02428	0.49465	H	-15.50998	-3.43385	1.99562
N	-11.70346	4.04042	0.02326	H	-16.34823	-2.10812	1.15829
N	11.70346	4.04042	-0.02331	H	-15.84453	-3.55766	0.26037
O	-10.49852	-0.56644	-0.00408	H	16.34825	-2.10813	-1.15817
O	10.49852	-0.56643	0.00407	H	15.84455	-3.55764	-0.26022
C	13.06578	3.8441	-0.10664	H	15.51002	-3.43388	-1.99549
C	-13.06578	3.84409	0.10662	H	-0.00001	3.19236	0.00008
C	13.89038	4.98497	-0.1156	H	0.	-1.75924	0.00002
C	15.26595	4.85012	-0.16773	C	-6.43042	-2.22143	-0.90809
C	15.84599	3.57656	-0.20436	C	-4.35917	-2.54576	0.37224
C	15.02749	2.45792	-0.2061	C	-7.64174	-1.49362	-0.99998
C	13.61993	2.53817	-0.1729	C	-8.68604	-2.07138	-1.75136
C	-13.61993	2.53816	0.17289	C	-8.56219	-3.30116	-2.37699
C	-15.02749	2.45791	0.20611	C	-7.34877	-3.98986	-2.29071
C	-15.84599	3.57655	0.20438	C	-6.29188	-3.45269	-1.57505
C	-15.26595	4.85011	0.16772	C	-4.80113	-3.76971	0.90797
C	-13.89038	4.98496	0.11558	C	-3.91043	-4.63094	1.5273
C	-11.7554	-1.02117	0.22735	C	-2.56101	-4.28601	1.64967
C	11.75541	-1.02117	-0.22733	C	-2.13556	-3.05895	1.16765
C	12.87976	-0.18421	-0.34763	C	-3.00488	-2.15674	0.52063
C	14.1018	-0.83352	-0.62881	N	-5.30546	-1.68682	-0.23311
C	14.22827	-2.21161	-0.75657	H	-9.61574	-1.51422	-1.85744
C	13.0709	-2.9967	-0.59853	H	-9.39336	-3.71286	-2.95399
C	11.84398	-2.41441	-0.34199	H	-7.21388	-4.94146	-2.81083
C	-11.84397	-2.41442	0.34204	H	-5.33571	-3.97569	-1.55278
C	-13.07089	-2.9967	0.59862	H	-5.85763	-4.03326	0.85633
C	-14.22825	-2.21161	0.75666	H	-4.27959	-5.57095	1.94481
C	-14.10179	-0.83352	0.62887	H	-1.86023	-4.95827	2.14946
C	-12.87976	-0.18422	0.34765	H	-1.09783	-2.76209	1.3285

Table S8. Cartesian coordinates of **DPA-B6** at the optimized S_1 geometry.

C	-15.05266	-2.0146	-0.74044	B	1.94649	1.45747	6.19686
C	-13.97866	-2.1815	0.14165	B	-20.64639	-3.22937	-4.1029
C	-12.77694	-1.51597	-0.12456	N	-3.89256	1.87289	0.84705
C	-12.60798	-0.70795	-1.27075	C	-21.97124	-1.9102	-8.93942
C	-13.73158	-0.51874	-2.10253	C	5.11701	5.28507	4.71679
C	-14.96924	-1.15236	-1.85571	C	-10.21001	-0.15582	-0.45648
N	-16.22894	-2.6931	-0.52824	C	-10.50529	-1.00934	0.6548
N	-11.71665	-1.6443	0.74177	C	-9.56621	-1.22462	1.66987
C	-17.30438	-2.7446	-1.37651	C	-8.32828	-0.57308	1.63724

C	-17.32811	-1.9429	-2.55669	C	-8.01447	0.36459	0.60143
C	-18.41901	-2.18539	-3.41873	C	-8.97507	0.51244	-0.41272
C	-19.48889	-3.04594	-3.13494	H	-14.07483	-2.82758	1.01948
C	-19.44307	-3.74215	-1.89729	H	-16.27086	-3.29217	0.28537
C	-18.35604	-3.61029	-1.03784	H	-11.86106	-2.22895	1.55482
C	-3.98188	-0.3282	3.79981	H	-18.31171	-4.18699	-0.10956
C	-5.29584	-0.80801	3.74669	H	-5.661	-1.51452	4.49817
C	-6.13555	-0.36351	2.71953	H	-3.53183	-1.35512	5.50585
C	-5.70656	0.57974	1.75977	H	-7.70386	-1.52249	3.32391
C	-4.36005	0.99671	1.81539	H	-1.76365	-1.37628	6.91603
C	-3.47179	0.54888	2.81755	H	-8.02016	3.01795	-0.86914
N	-3.14923	-0.71581	4.82212	H	-7.16739	5.16483	-1.72163
N	-7.41976	-0.84699	2.62628	H	-4.79979	5.83017	-1.25024
C	-1.89698	-0.22415	5.08437	H	-3.30843	4.29192	-0.03192
C	-1.28026	0.69216	4.18083	H	-2.91018	2.34866	-1.55229
C	-0.05481	1.23251	4.6267	H	-0.54085	2.3316	-2.2173
C	0.61106	0.84944	5.79971	H	1.23018	1.80421	-0.52425
C	-0.02062	-0.13613	6.60481	H	0.57449	1.25921	1.78805
C	-1.26746	-0.65214	6.2636	H	-20.43285	-5.05871	-0.70093
C	-4.79019	2.72587	0.16139	H	0.1501	-1.24433	8.3039
C	-2.51635	1.89244	0.52835	H	1.77449	-1.47889	9.87238
C	-6.14607	2.35224	-0.03171	H	4.01577	-0.85473	10.71609
C	-6.96679	3.26445	-0.72683	H	5.39401	0.82325	9.47367
C	-6.49756	4.48358	-1.19159	H	4.50397	1.86516	7.43838
C	-5.17287	4.85199	-0.93795	H	-23.2282	-3.86365	-5.18776
C	-4.32997	3.98547	-0.25869	H	-24.95395	-5.33745	-4.25419
C	-2.14203	2.15809	-0.80279	H	-24.56174	-6.4778	-2.06229
C	-0.80931	2.13844	-1.17588	H	-22.45046	-6.09184	-0.83026
C	0.17776	1.83509	-0.23277	H	4.19005	3.2915	6.28439
C	-0.19419	1.53759	1.06876	H	3.31857	5.40846	2.66712
C	-1.53685	1.55533	1.4967	H	1.27349	4.01506	2.34884
B	-11.27322	-0.05187	-1.56518	H	-18.13084	-0.32924	-6.75993
B	-16.1677	-0.94676	-2.76676	H	-19.68095	-0.4414	-8.71219
B	-6.65856	1.0872	0.69472	H	-22.17981	-3.17258	-6.5619
B	-2.02356	1.00606	2.86433	H	-21.44317	-2.28022	-9.83309
N	-20.48411	-4.56676	-1.58319	H	-22.81407	-2.58799	-8.74352
N	0.61664	-0.54639	7.73977	H	-22.38747	-0.92295	-9.19777
O	-18.38323	-1.53734	-4.59525	H	5.65419	5.00669	5.63445
O	0.47125	2.1948	3.85053	H	5.82078	5.19047	3.87395
C	1.86195	-0.13773	8.17365	H	4.85005	6.35148	4.7944
C	-21.62863	-4.76266	-2.33021	H	-9.79958	-1.91015	2.49101
C	2.37259	-0.73252	9.34088	H	-8.74308	1.19568	-1.22873
C	3.62767	-0.38412	9.80941	C	-14.76689	0.95267	-3.7217

C	4.39671	0.55736	9.11688	C	-12.34984	0.56481	-3.7843
C	3.8822	1.14672	7.97212	C	-16.0433	0.34416	-3.61908
C	2.60335	0.84219	7.46192	C	-17.1421	1.07487	-4.11398
C	-21.80394	-4.12629	-3.58745	C	-17.00676	2.33302	-4.67943
C	-23.0324	-4.3616	-4.23836	C	-15.74088	2.9242	-4.74236
C	-24.01648	-5.1863	-3.71485	C	-14.63415	2.24626	-4.26117
C	-23.79893	-5.8206	-2.48697	C	-12.28591	0.80687	-5.16732
C	-22.61744	-5.60848	-1.79759	C	-11.06849	1.05348	-5.78331
C	-19.31359	-1.67499	-5.57252	C	-9.88979	1.04195	-5.03148
C	1.61499	2.8665	4.13365	C	-9.94763	0.74643	-3.67807
C	2.41923	2.601	5.25772	C	-11.1626	0.4898	-3.00941
C	3.5531	3.42778	5.40964	N	-13.61546	0.32201	-3.1996
C	3.90051	4.43037	4.51131	H	-18.13577	0.63757	-4.02736
C	3.07029	4.62901	3.39333	H	-17.88573	2.86837	-5.04596
C	1.93544	3.8627	3.2034	H	-15.6193	3.93347	-5.14299
C	-19.03476	-0.9406	-6.73142	H	-13.65732	2.72993	-4.27115
C	-19.90099	-1.0139	-7.80645	H	-13.19794	0.78065	-5.76422
C	-21.05087	-1.8226	-7.75707	H	-11.03764	1.23195	-6.86067
C	-21.2974	-2.53211	-6.58715	H	-8.92709	1.23009	-5.51258
C	-20.461	-2.48105	-5.45142	H	-9.01486	0.67083	-3.11726

Table S9. Cartesian coordinates of **DPA-B8** at the optimized S_0 geometry.

C	-10.75529	2.17472	-0.33631	C	-12.8348	-1.00706	-0.96926
C	-9.40984	2.54591	-0.26317	C	-13.96539	-1.44819	-1.68757
C	-8.44414	1.54067	-0.16347	C	-14.025	-2.69274	-2.29295
C	-8.7967	0.16974	-0.1095	C	-12.9149	-3.53916	-2.21959
C	-10.16139	-0.16699	-0.24031	C	-11.77829	-3.14061	-1.53608
C	-11.16274	0.82043	-0.36355	C	-10.28688	-3.62173	0.91928
N	-11.72515	3.14351	-0.38875	C	-9.50764	-4.58668	1.53602
N	-7.1148	1.87655	-0.10996	C	-8.1217	-4.42607	1.62697
C	-13.08382	2.94843	-0.3044	C	-7.54489	-3.27498	1.11591
C	-13.62127	1.63092	-0.29925	C	-8.29798	-2.27311	0.46997
C	-15.01285	1.55886	-0.0731	N	-10.52943	-1.50564	-0.24723
C	-15.86528	2.66855	0.03462	H	-14.81263	-0.77078	-1.78422
C	-15.2683	3.95235	-0.06884	H	-14.91776	-2.99592	-2.84459
C	-13.89138	4.0928	-0.21666	H	-12.92292	-4.5081	-2.72492
C	1.19795	0.5588	0.09942	H	-10.90221	-3.78919	-1.52419
C	0.00001	1.2723	0.00053	H	-11.37013	-3.74098	0.89321
C	-1.19793	0.55882	-0.09846	H	-9.99158	-5.46189	1.97648
C	-1.22424	-0.85746	-0.14186	H	-7.5079	-5.17972	2.12502
C	0.00001	-1.54558	0.00035	H	-6.47349	-3.11824	1.25179

C	1.22425	-0.85748	0.14264	C	5.12793	-1.21155	0.12343
N	2.38969	1.23588	0.16275	C	6.26875	-0.40331	0.04145
N	-2.38967	1.23591	-0.16171	B	7.72971	-0.8895	-0.06996
C	3.64896	0.67812	0.16801	C	6.05019	1.00344	0.09924
C	3.81349	-0.73628	0.2118	C	8.79671	0.1697	0.10989
C	-1.06068	-3.64282	-0.61739	C	8.29792	-2.27306	-0.46988
C	1.06068	-3.6429	0.61783	N	7.11482	1.87651	0.1108
C	-2.35533	-3.0775	-0.72129	C	4.75613	1.53006	0.1373
C	-3.34776	-3.8353	-1.37598	C	8.44416	1.54063	0.16412
C	-3.09494	-5.08751	-1.91169	C	10.16142	-0.16705	0.24047
C	-1.79808	-5.60444	-1.83998	C	9.69536	-2.47661	-0.35421
C	-0.79106	-4.88887	-1.21291	C	7.54472	-3.27485	-1.11584
C	0.79105	-4.889	1.21323	H	6.89267	2.8624	0.14866
C	1.79806	-5.60464	1.84023	H	4.60956	2.61512	0.14261
C	3.09492	-5.08773	1.912	C	9.40987	2.54584	0.26387
C	3.34776	-3.83547	1.3764	C	11.16279	0.82035	0.36374
C	2.35534	-3.07759	0.72178	N	10.52947	-1.50571	0.24713
B	-7.72972	-0.88948	0.07033	C	10.28675	-3.62163	-0.91965
B	-12.63191	0.45804	-0.49402	C	8.12146	-4.42586	-1.62715
B	-2.53511	-1.59374	-0.31821	H	6.47331	-3.11807	-1.25155
B	2.53512	-1.59379	0.31888	C	10.75533	2.17464	0.33678
N	-16.07797	5.0511	0.00714	H	9.12005	3.60037	0.27475
O	-15.51742	0.32232	0.06492	B	12.63198	0.45794	0.49391
C	-17.45184	5.04592	0.12485	C	11.7322	-1.89208	0.88869
C	-18.17787	3.8304	0.23578	C	9.50741	-4.58649	-1.53642
C	-19.58188	3.9451	0.30162	H	11.36999	-3.7409	-0.89376
C	-20.23914	5.16545	0.28998	H	7.50758	-5.17944	-2.12521
C	-19.49112	6.34601	0.20898	N	11.7252	3.14343	0.38927
C	-18.11182	6.28929	0.12313	C	12.83495	-1.00723	0.96886
C	-16.81827	0.0505	0.337	C	11.77854	-3.14086	1.53553
C	-17.09437	-1.31433	0.48888	H	9.99129	-5.46164	-1.97706
C	-18.38178	-1.71588	0.79152	C	13.08385	2.94837	0.30468
C	-19.41511	-0.77522	0.95945	H	11.40941	4.10395	0.37953
C	-19.10424	0.56902	0.79259	C	13.96567	-1.44848	1.6869
C	-17.81317	1.03697	0.46373	C	12.91526	-3.53952	2.21879
B	-17.36167	2.5086	0.25429	H	10.90245	-3.78945	1.52369
N	0.	-2.93579	0.00026	C	14.02538	-2.69312	2.29208
C	-20.80325	-1.22484	1.31165	H	14.81292	-0.77109	1.78351
C	-6.26874	-0.40328	-0.04087	H	12.92336	-4.50854	2.72397
C	-6.05018	1.00348	-0.09844	H	14.91823	-2.99639	2.84351
C	-4.75611	1.5301	-0.13633	H	5.2736	-2.29222	0.11824
C	-3.64894	0.67815	-0.1671	C	13.62131	1.63086	0.29921
C	-3.81348	-0.73623	-0.21109	C	15.01286	1.55885	0.07283

C	-5.12792	-1.21151	-0.12288	C	15.86527	2.66856	-0.03482
H	-9.12001	3.60044	-0.27384	O	15.5174	0.32234	-0.06548
H	-11.40938	4.10403	-0.37884	C	15.26829	3.95234	0.06897
H	-6.89265	2.86244	-0.14764	B	17.36162	2.50866	-0.25477
H	-13.43662	5.08693	-0.24882	C	16.81822	0.05055	-0.33774
H	0.00001	2.36594	0.00059	C	13.89139	4.09276	0.21703
H	2.34133	2.24564	0.13343	N	16.07794	5.0511	-0.00692
H	-2.34131	2.24566	-0.13228	C	18.1778	3.83048	-0.2363
H	-4.34074	-3.40013	-1.5005	C	17.81311	1.03706	-0.46443
H	-3.88706	-5.64517	-2.41603	C	17.09429	-1.31424	-0.48992
H	-1.56057	-6.56529	-2.30312	C	17.45178	5.04596	-0.125
H	0.22357	-5.28706	-1.20257	H	15.63391	5.95715	0.06254
H	-0.22358	-5.28718	1.20284	C	19.58179	3.94524	-0.30255
H	1.56054	-6.56554	2.30328	C	19.10415	0.56914	-0.79346
H	3.88704	-5.64544	2.41629	C	18.38167	-1.71575	-0.79275
H	4.34074	-3.40031	1.50097	C	18.11172	6.28935	-0.12326
H	-15.63394	5.95716	-0.06209	C	20.23902	5.16561	-0.29091
H	-20.182	3.03666	0.34842	H	20.18194	3.03683	-0.34969
H	-21.32906	5.20634	0.34068	C	19.415	-0.77507	-0.96055
H	-19.9926	7.31691	0.20375	H	19.90456	1.2945	-0.94355
H	-17.52672	7.21038	0.04506	H	18.59616	-2.78141	-0.91422
H	-16.27891	-2.03018	0.36987	C	19.491	6.34613	-0.20949
H	-18.59631	-2.78157	0.9127	H	17.52661	7.21042	-0.04488
H	-19.90466	1.29436	0.94272	H	21.32893	5.20653	-0.34194
H	-20.81517	-1.78302	2.26159	C	20.80322	-1.22465	-1.31252
H	-21.48749	-0.37108	1.41576	H	19.99245	7.31704	-0.20426
H	-21.21585	-1.89407	0.5395	H	20.81478	-1.78622	-2.26043
H	-4.60954	2.61516	-0.14148	H	21.48656	-0.37062	-1.42026
H	-5.27359	-2.29219	-0.11787	H	21.2172	-1.89071	-0.53834
C	-11.73206	-1.89192	-0.88904	H	13.43663	5.08688	0.24944
C	-9.6954	-2.47664	0.35408	H	16.27883	-2.0301	-0.37103

Table S10. Cartesian coordinates of **DPA-B8** at the optimized S_1 geometry.

C	-14.96278	-2.20317	-0.82627	C	-16.1867	0.51879	-3.2596
C	-13.8179	-2.48683	-0.07344	C	-17.3196	1.30881	-3.54136
C	-12.63979	-1.78973	-0.35554	C	-17.22481	2.63031	-3.94658
C	-12.5672	-0.83595	-1.3962	C	-15.96296	3.22259	-4.05566
C	-13.75676	-0.53713	-2.09357	C	-14.82354	2.48769	-3.77717
C	-14.97289	-1.19967	-1.82001	C	-12.5697	1.18932	-5.07039
N	-16.11984	-2.90737	-0.60204	C	-11.40679	1.50676	-5.75391
N	-11.50904	-2.02966	0.38842	C	-10.16492	1.38212	-5.12391
C	-17.26794	-2.85103	-1.35197	C	-10.11057	0.90699	-3.82327

C	-17.39591	-1.89356	-2.39916	C	-11.26786	0.57737	-3.08803
C	-18.56293	-2.0226	-3.18216	N	-13.72992	0.44087	-3.07649
C	-19.60145	-2.93063	-2.92913	H	-18.30533	0.86394	-3.41321
C	-19.44292	-3.79523	-1.81393	H	-18.12797	3.21012	-4.15062
C	-18.28323	-3.77013	-1.04525	H	-15.86698	4.27562	-4.33167
C	-3.55345	-0.99339	2.95908	H	-13.84697	2.97019	-3.81933
C	-4.86794	-1.47445	2.96176	H	-13.53038	1.25277	-5.58199
C	-5.78991	-0.92543	2.06299	H	-11.46886	1.83014	-6.79579
C	-5.44012	0.12916	1.19001	H	-9.24543	1.62257	-5.66258
C	-4.09384	0.55596	1.18408	H	-9.13424	0.74467	-3.36375
C	-3.1245	-0.00562	2.04425	C	0.50686	0.36373	3.47676
N	-2.63989	-1.48831	3.86133	C	1.29551	-0.13824	4.52392
N	-7.07659	-1.41134	2.01712	B	2.71822	0.31678	4.8984
C	-1.34871	-1.06277	4.03373	C	0.7205	-1.19585	5.29574
C	-0.79193	-0.06612	3.17125	C	3.44318	-0.54021	5.91617
C	-4.67045	2.47428	-0.18892	C	3.49726	1.59445	4.50635
C	-2.3601	1.66931	-0.10097	N	1.44418	-1.80183	6.29226
C	-6.03394	2.10457	-0.31975	C	-0.58779	-1.63024	5.06269
C	-6.92098	3.08326	-0.81418	C	2.76064	-1.56509	6.6101
C	-6.50661	4.36272	-1.1474	C	4.81705	-0.36934	6.18777
C	-5.16833	4.72049	-0.95401	C	4.85787	1.68775	4.89515
C	-4.26237	3.79047	-0.47061	C	2.90584	2.74838	3.95212
C	-2.08344	2.14538	-1.39512	H	0.97916	-2.54198	6.80141
C	-0.77571	2.26751	-1.83607	H	-1.02004	-2.41787	5.68839
C	0.28412	1.89719	-1.00188	C	3.38568	-2.32993	7.59909
C	0.00931	1.37231	0.25068	C	5.49139	-1.14076	7.15873
C	-1.30569	1.2299	0.74053	N	5.52749	0.58448	5.47443
B	-11.25625	-0.14973	-1.72255	C	5.54483	2.90951	4.78422
B	-16.24799	-0.87504	-2.57864	C	3.59987	3.93709	3.79212
B	-6.47785	0.75056	0.27814	H	1.85048	2.71525	3.67677
B	-1.67821	0.44398	2.01789	C	4.73558	-2.09096	7.88015
N	-20.44914	-4.67369	-1.52943	H	2.82964	-3.09609	8.14741
O	-18.63773	-1.21283	-4.25055	B	6.97113	-0.95068	7.44413
C	-21.65396	-4.77947	-2.193	C	6.92291	0.43056	5.30557
C	-21.94439	-3.97308	-3.32495	C	4.92571	4.01762	4.22834
C	-23.22474	-4.13416	-3.89324	H	6.56597	2.99255	5.15715
C	-24.15451	-5.04179	-3.41029	H	3.10564	4.81113	3.36172
C	-23.82494	-5.84117	-2.30997	N	5.36184	-2.79802	8.8765
C	-22.5884	-5.71003	-1.70336	C	7.70716	-0.24166	6.27547
C	-19.65363	-1.22165	-5.14881	C	7.50464	0.8705	4.10166
C	-19.4836	-0.32196	-6.20839	H	5.4747	4.95986	4.15914
C	-20.44479	-0.25177	-7.19903	C	6.62823	-2.58208	9.35959
C	-21.58395	-1.07686	-7.16464	H	4.80474	-3.48239	9.37028

C	-21.72056	-1.95235	-6.09382	C	9.07726	-0.40964	5.98937
C	-20.78285	-2.05403	-5.0435	C	8.8574	0.69384	3.86767
B	-20.84161	-2.99147	-3.80643	H	6.8794	1.32551	3.33341
N	-3.7114	1.55823	0.30542	C	9.65754	0.05652	4.82085
C	-22.60872	-1.00462	-8.25889	H	9.69413	-0.94594	6.70893
C	-10.10307	-0.39046	-0.73039	H	9.28555	1.0302	2.92015
C	-10.30705	-1.37522	0.28614	H	10.72273	-0.09906	4.63469
C	-9.29083	-1.70257	1.18868	H	0.938	1.14576	2.85244
C	-8.0611	-1.03551	1.1411	C	7.48492	-1.63216	8.73212
C	-7.8348	0.02311	0.20549	C	8.70905	-1.4062	9.39716
C	-8.86956	0.27903	-0.70497	C	9.15091	-2.11403	10.52453
H	-13.84302	-3.24495	0.71492	O	9.4706	-0.42151	8.8936
H	-16.09134	-3.61361	0.12109	C	8.27816	-3.10619	11.04435
H	-11.58811	-2.71536	1.1279	B	10.50284	-1.83773	11.16269
H	-18.1532	-4.47197	-0.21653	C	10.66177	-0.03263	9.4125
H	-5.16962	-2.26592	3.65462	C	7.02372	-3.32437	10.48307
H	-2.97195	-2.19497	4.50464	N	8.69175	-3.82642	12.1285
H	-7.3011	-2.16679	2.65147	C	10.88785	-2.78437	12.33205
H	-7.9804	2.83569	-0.89732	C	11.26624	-0.64457	10.52568
H	-7.22529	5.09497	-1.52257	C	11.25054	1.04575	8.74056
H	-4.83305	5.7405	-1.15617	C	9.91776	-3.73145	12.75371
H	-3.23004	4.08881	-0.28725	H	8.05467	-4.52431	12.48899
H	-2.90423	2.40142	-2.06525	C	12.12227	-2.81121	13.0131
H	-0.58313	2.62923	-2.84889	C	12.49084	-0.08206	10.94605
H	1.31647	1.98648	-1.34793	C	12.46074	1.54306	9.18594
H	0.83836	1.01644	0.86445	C	10.17565	-4.61215	13.82005
H	-20.31621	-5.28605	-0.73557	C	12.38782	-3.68333	14.05738
H	-23.50663	-3.50886	-4.74012	H	12.91143	-2.12885	12.69825
H	-25.13546	-5.13094	-3.88173	C	13.10364	0.99004	10.30854
H	-24.54392	-6.56554	-1.91927	H	12.98406	-0.49355	11.82738
H	-18.58747	0.30109	-6.22868	C	11.39838	-4.58418	14.46695
H	-20.31011	0.44933	-8.02787	H	9.40546	-5.32502	14.12953
H	-22.59563	-2.60323	-6.08276	H	13.3599	-3.66942	14.55472
H	-22.16428	-1.23405	-9.24087	C	14.40717	1.55442	10.79334
H	-22.33389	-6.32421	-0.83433	H	12.9197	2.38601	8.66115
H	-23.43	-1.71496	-8.08845	H	11.58891	-5.27541	15.29171
H	-23.04679	0.00404	-8.33188	H	14.30781	2.61774	11.06544
H	-9.45708	-2.48571	1.93555	H	14.77507	1.01517	11.67766
H	-8.70298	1.0558	-1.45077	H	15.18489	1.49291	10.01485
C	-14.91853	1.13453	-3.40138	H	6.34237	-4.06008	10.9198
C	-12.51792	0.76056	-3.73235	H	10.7332	1.47315	7.87951

Table S11. Cartesian coordinates of **DPA-B10** at the optimized S_0 geometry.

C	15.29438	2.8566	0.24155	B	-2.61884	-2.65059	0.20857
C	13.91163	3.04522	0.16101	C	-1.21113	-0.54753	0.05068
C	13.08571	1.91895	0.10757	C	-3.81971	-1.74387	0.04437
C	13.61564	0.60514	0.10228	C	-2.99467	-4.10051	0.60106
C	15.01232	0.45558	0.2415	N	-2.38405	0.17424	0.06316
C	15.87532	1.56946	0.32348	C	0.	0.14809	-0.00004
N	16.12991	3.94476	0.24786	C	-3.6566	-0.33658	0.0119
N	11.72325	2.0743	0.05425	C	-5.12569	-2.2604	-0.09641
C	17.50283	3.92443	0.1679	C	-4.35349	-4.48665	0.49488
C	18.20712	2.68931	0.22069	C	-2.11209	-4.9915	1.2454
C	19.59741	2.78862	-0.00298	H	-2.29685	1.18148	0.03669
C	20.29928	3.99357	-0.1605	H	0.	1.24303	-0.00005
C	19.53974	5.19201	-0.11593	C	-4.7495	0.53158	-0.06545
C	18.15537	5.1588	0.02632	C	-6.24796	-1.41376	-0.22178
C	3.65661	-0.33658	-0.01195	N	-5.31121	-3.63772	-0.11195
C	4.7495	0.53158	0.06537	C	-4.78698	-5.69182	1.07761
C	6.03309	-0.0145	0.15824	C	-2.52991	-6.2042	1.76875
C	6.24797	-1.41376	0.22176	H	-1.06937	-4.69539	1.37178
C	5.1257	-2.2604	0.09641	C	-6.03308	-0.0145	-0.15829
C	3.81972	-1.74387	-0.04438	H	-4.60345	1.61539	-0.0535
N	2.38406	0.17424	-0.06323	B	-7.6457	-1.96663	-0.40122
N	7.12485	0.81575	0.19464	C	-6.45781	-4.18924	-0.73437
C	1.21113	-0.54753	-0.05073	C	-3.88427	-6.54238	1.69476
C	1.23789	-1.97125	-0.09613	H	-5.8458	-5.95032	1.065
C	6.45781	-4.18923	0.73443	H	-1.81897	-6.86817	2.26525
C	4.3535	-4.48666	-0.49482	N	-7.12485	0.81575	-0.19471
C	7.66588	-3.45526	0.82686	C	-7.66588	-3.45528	-0.82681
C	8.75121	-4.06638	1.4876	C	-6.35699	-5.45252	-1.34576
C	8.66769	-5.33423	2.03961	H	-4.24762	-7.46779	2.14835
C	7.45106	-6.01995	1.97851	C	-8.44711	0.42989	-0.19381
C	6.35698	-5.45249	1.34585	H	-6.9426	1.80937	-0.14634
C	4.78698	-5.69185	-1.07752	C	-8.75121	-4.06642	-1.48753
C	3.88428	-6.54243	-1.69464	C	-7.45107	-6.02	-1.9784
C	2.52992	-6.20425	-1.76865	H	-5.40421	-5.98198	-1.34301
C	2.11209	-4.99153	-1.24533	C	-8.6677	-5.33428	-2.03951
C	2.99467	-4.10052	-0.60101	H	-9.67827	-3.50241	-1.60316
B	12.69798	-0.59145	-0.03503	H	-7.34428	-6.99802	-2.45402
B	17.3786	1.40701	0.46692	H	-9.52782	-5.77531	-2.54792
B	7.64571	-1.96663	0.40123	H	0.	-3.70912	0.00001
B	2.61884	-2.6506	-0.20857	C	-8.79781	-0.94886	-0.2691
N	20.20012	6.38209	-0.24273	C	-10.16311	-1.24775	-0.18206
O	20.25838	1.6231	-0.08609	C	-11.18605	-0.2983	-0.06797

C	21.56373	6.55037	-0.35989	H	-10.45135	-2.29901	-0.20482
C	22.44235	5.43591	-0.41384	B	-12.69797	-0.59144	0.03504
C	23.81984	5.72958	-0.4851	C	-10.7834	1.06835	-0.08473
C	24.31267	7.02432	-0.53038	C	-13.61564	0.60514	-0.10229
C	23.417	8.09988	-0.50438	C	-13.44399	-1.90215	0.3859
C	22.0563	7.86787	-0.41628	N	-11.72324	2.07431	-0.05432
C	21.58608	1.51027	-0.34039	C	-9.43116	1.41973	-0.12501
C	22.03814	0.18727	-0.42657	C	-13.0857	1.91896	-0.10762
C	23.37025	-0.05719	-0.70207	C	-15.01232	0.45558	-0.24148
C	24.27517	1.001	-0.9075	C	-14.8556	-1.91564	0.26471
C	23.79079	2.29974	-0.80702	C	-12.83198	-3.01828	0.99252
C	22.44611	2.61083	-0.50854	H	-11.37271	3.02293	-0.06069
B	21.80551	4.01946	-0.37048	H	-9.14163	2.47546	-0.10248
N	5.31121	-3.63772	0.11199	C	-13.91163	3.04523	-0.16108
C	25.71424	0.71983	-1.22877	C	-15.87531	1.56946	-0.32348
C	11.18606	-0.2983	0.06796	N	-15.5524	-0.82197	-0.29915
C	10.78341	1.06834	0.08468	C	-15.59516	-2.99271	0.78754
C	9.43117	1.41973	0.12493	C	-13.5575	-4.10134	1.46116
C	8.44711	0.42989	0.19376	H	-11.74973	-3.01027	1.13257
C	8.79781	-0.94886	0.26908	C	-15.29438	2.8566	-0.2416
C	10.16312	-1.24775	0.18207	H	-13.48642	4.0524	-0.13201
H	13.48643	4.0524	0.1319	B	-17.3786	1.40701	-0.46689
H	15.69209	4.85487	0.19833	C	-16.79177	-1.02276	-0.95545
H	11.37271	3.02293	0.0606	C	-14.95223	-4.07426	1.36678
H	17.57526	6.08586	0.01352	H	-16.68455	-2.96632	0.75802
H	4.60346	1.61539	0.05339	H	-13.05002	-4.94743	1.92957
H	2.29685	1.18148	-0.03678	N	-16.12991	3.94476	-0.24793
H	6.94261	1.80937	0.14624	C	-17.76878	0.00133	-1.00173
H	9.67826	-3.50238	1.60322	C	-16.99716	-2.22921	-1.64992
H	9.52781	-5.77524	2.54804	H	-15.54877	-4.89373	1.77519
H	7.34427	-6.99796	2.45416	C	-17.50283	3.92444	-0.16795
H	5.40421	-5.98195	1.34311	H	-15.69208	4.85487	-0.19845
H	5.84581	-5.95035	-1.06489	C	-18.94322	-0.26033	-1.73738
H	4.24763	-7.46785	-2.14821	C	-18.17238	-2.44942	-2.34857
H	1.81897	-6.86823	-2.26513	H	-16.21335	-2.98667	-1.66259
H	1.06938	-4.69543	-1.37172	C	-19.16158	-1.46257	-2.39011
H	19.64186	7.22499	-0.21517	H	-19.69407	0.52529	-1.80855
H	24.53346	4.90604	-0.48978	H	-18.30448	-3.38911	-2.89059
H	25.3883	7.20435	-0.58305	H	-20.08319	-1.62499	-2.95359
H	23.78778	9.12705	-0.54439	C	-18.20711	2.68932	-0.22067
H	21.35565	8.70739	-0.38159	C	-19.5974	2.78863	0.00301
H	21.32082	-0.62209	-0.27827	C	-20.29928	3.99358	0.16049
H	23.72248	-1.0904	-0.77142	O	-20.25835	1.6231	0.08623

H	24.4927	3.11496	-0.98605	C	-19.53974	5.19202	0.11587
H	25.81072	0.12943	-2.15412	B	-21.80551	4.01947	0.37048
H	26.28467	1.6496	-1.36308	C	-21.58608	1.51027	0.34033
H	26.19851	0.14133	-0.42559	C	-18.15537	5.15881	-0.02641
H	9.14164	2.47546	0.10238	N	-20.20011	6.3821	0.24262
H	10.45136	-2.29901	0.20486	C	-22.44233	5.43594	0.41401
C	16.79176	-1.02275	0.95553	C	-22.44614	2.61083	0.50838
C	14.8556	-1.91566	-0.26463	C	-22.03813	0.18726	0.42645
C	17.76877	0.00134	1.00181	C	-21.56369	6.55039	0.35997
C	18.9432	-0.2603	1.73748	H	-19.64184	7.225	0.21502
C	19.16155	-1.46253	2.39025	C	-23.81979	5.72965	0.48556
C	18.17234	-2.44938	2.34872	C	-23.79088	2.2997	0.80658
C	16.99713	-2.22918	1.65004	C	-23.37028	-0.05722	0.70172
C	15.59517	-2.99274	-0.78742	C	-22.05624	7.8679	0.41645
C	14.95225	-4.07431	-1.36664	C	-24.31259	7.0244	0.53095
C	13.55752	-4.10139	-1.46104	H	-24.53344	4.90613	0.4904
C	12.83199	-3.01831	-0.99244	C	-24.27526	1.00095	0.90693
C	13.444	-1.90217	-0.38584	H	-24.49284	3.11489	0.98549
N	15.5524	-0.82197	0.29921	H	-23.7225	-1.09044	0.77103
H	19.69406	0.52532	1.80864	C	-23.4169	8.09994	0.5048
H	20.08315	-1.62494	2.95374	H	-21.35557	8.7074	0.38167
H	18.30443	-3.38905	2.89077	H	-25.38821	7.20444	0.58386
H	16.21332	-2.98664	1.66272	C	-25.71446	0.71977	1.22763
H	16.68456	-2.96635	-0.75789	H	-23.78766	9.12711	0.54488
H	15.5488	-4.89378	-1.77502	H	-25.81121	0.12699	2.15141
H	13.05005	-4.94749	-1.92943	H	-26.28435	1.64952	1.36437
H	11.74975	-3.01031	-1.1325	H	-26.19913	0.14367	0.42294
C	0.	-2.61876	0.	H	-17.57527	6.08587	-0.01364
C	-1.23788	-1.97125	0.09611	H	-21.32076	-0.62208	0.27831

Table S12. Cartesian coordinates of **DPA-B10** at the optimized S_1 geometry.

C	-15.29004	2.8626	-0.26683	B	2.62289	-2.69556	-0.19509
C	-13.90423	3.04104	-0.19617	C	1.21321	-0.59021	-0.03758
C	-13.08534	1.90979	-0.14231	C	3.82287	-1.78411	-0.03337
C	-13.62075	0.59972	-0.1265	C	3.00254	-4.14181	-0.59045
C	-15.0194	0.46047	-0.25764	N	2.3814	0.12873	-0.03862
C	-15.87771	1.57901	-0.33888	C	-0.00001	0.10581	0.00014
N	-16.11857	3.95695	-0.27307	C	3.65865	-0.38023	0.0078
N	-11.72009	2.05727	-0.09944	C	5.13265	-2.29494	0.10738
C	-17.48967	3.94546	-0.18662	C	4.36663	-4.52085	-0.49589
C	-18.20203	2.71273	-0.22498	C	2.12571	-5.0473	-1.22441
C	-19.59048	2.82232	0.00703	H	2.2916	1.1359	-0.00642

C	-20.2847	4.03252	0.15741	H	-0.00002	1.20059	0.00018
C	-19.51845	5.22663	0.09656	C	4.75152	0.48968	0.08938
C	-18.13535	5.18467	-0.05382	C	6.25635	-1.44847	0.23895
C	-3.65868	-0.38024	-0.00761	N	5.32018	-3.66988	0.1174
C	-4.75155	0.48966	-0.08918	C	4.81248	-5.7156	-1.08894
C	-6.03909	-0.05216	-0.17983	C	2.55621	-6.2525	-1.75574
C	-6.25637	-1.4485	-0.23884	H	1.07688	-4.76831	-1.3373
C	-5.13266	-2.29497	-0.10728	C	6.03907	-0.05212	0.17999
C	-3.82289	-1.78413	0.03351	H	4.60264	1.57356	0.08219
N	-2.38143	0.12872	0.03886	B	7.65585	-2.00162	0.41223
N	-7.13145	0.78234	-0.21765	C	6.46655	-4.22522	0.73546
C	-1.21324	-0.59022	0.03782	C	3.91577	-6.57608	-1.70228
C	-1.24178	-2.02007	0.08391	H	5.87523	-5.95809	-1.08525
C	-6.46653	-4.22523	-0.73548	H	1.84709	-6.92452	-2.24437
C	-4.36664	-4.5209	0.4959	N	7.13142	0.78238	0.21781
C	-7.67744	-3.49165	-0.82701	C	7.67746	-3.49164	0.827
C	-8.76425	-4.11723	-1.4729	C	6.36069	-5.49183	1.33954
C	-8.67531	-5.38853	-2.01668	H	4.28505	-7.49448	-2.16461
C	-7.45457	-6.0693	-1.96309	C	8.44979	0.40214	0.21945
C	-6.36063	-5.4918	-1.33964	H	6.94739	1.77606	0.1722
C	-4.81248	-5.71569	1.08888	C	8.76428	-4.11723	1.47285
C	-3.91577	-6.5762	1.70218	C	7.45465	-6.06934	1.96295
C	-2.55621	-6.2526	1.75569	H	5.40386	-6.01388	1.33786
C	-2.12571	-5.04737	1.22444	C	8.67538	-5.38856	2.01657
C	-3.00255	-4.14185	0.59052	H	9.69712	-3.56212	1.58431
B	-12.7082	-0.60249	0.00865	H	7.34643	-7.04879	2.43483
B	-17.38342	1.42408	-0.47036	H	9.53761	-5.83774	2.51442
B	-7.65585	-2.00166	-0.41216	H	0.	-3.75759	0.00012
B	-2.6229	-2.69558	0.19525	C	8.80573	-0.98055	0.28566
N	-20.17152	6.42093	0.21593	C	10.17352	-1.2735	0.20201
O	-20.25736	1.6609	0.10521	C	11.19618	-0.3186	0.09856
C	-21.53366	6.59822	0.34148	H	10.46537	-2.32338	0.21845
C	-22.41799	5.48925	0.41172	B	12.70817	-0.60242	-0.0088
C	-23.79315	5.79207	0.49046	C	10.78616	1.04956	0.12325
C	-24.27798	7.09021	0.52795	C	13.62072	0.59978	0.12639
C	-23.37654	8.16018	0.48584	C	13.46049	-1.90709	-0.36661
C	-22.01773	7.91885	0.38971	N	11.72006	2.05733	0.09951
C	-21.584	1.55802	0.3701	C	9.43291	1.3964	0.1626
C	-22.04314	0.23876	0.47355	C	13.08531	1.90985	0.14232
C	-23.37481	0.00488	0.76101	C	15.01937	0.46051	0.25745
C	-24.27219	1.07031	0.961	C	14.87428	-1.9112	-0.2562
C	-23.78106	2.36525	0.84331	C	12.85556	-3.03078	-0.96712
C	-22.43669	2.66535	0.53278	H	11.36416	3.00403	0.1142

B	-21.78945	4.06898	0.377	H	9.13976	2.45121	0.14692
N	-5.32017	-3.6699	-0.11734	C	13.90421	3.04109	0.19626
C	-25.71059	0.8011	1.29506	C	15.87769	1.57904	0.33874
C	-11.19622	-0.31868	-0.0986	N	15.56655	-0.81388	0.309
C	-10.7862	1.04949	-0.12318	C	15.62058	-2.97917	-0.78681
C	-9.43295	1.39634	-0.16241	C	13.58863	-4.10626	-1.44234
C	-8.44982	0.40209	-0.21932	H	11.77219	-3.03506	-1.09738
C	-8.80575	-0.9806	-0.28562	C	15.29003	2.86264	0.26684
C	-10.17353	-1.27356	-0.20205	H	13.47197	4.0456	0.17521
H	-13.47198	4.04554	-0.17498	B	17.3834	1.4241	0.47013
H	-15.67484	4.86471	-0.23257	C	16.81145	-1.01041	0.9544
H	-11.36419	3.00396	-0.11415	C	14.98401	-4.06724	-1.36188
H	-17.55043	6.10879	-0.05339	H	16.70978	-2.9413	-0.76538
H	-4.60269	1.57354	-0.08192	H	13.08461	-4.95746	-1.90543
H	-2.29163	1.13589	0.00665	N	16.11856	3.95699	0.27318
H	-6.94742	1.77602	-0.17205	C	17.78507	0.01814	0.99498
H	-9.6971	-3.56215	-1.58434	C	17.02645	-2.21848	1.64385
H	-9.53753	-5.8377	-2.51457	H	15.5844	-4.8809	-1.77589
H	-7.34633	-7.04871	-2.43503	C	17.48966	3.94549	0.18669
H	-5.4038	-6.01383	-1.33797	H	15.67483	4.86475	0.23275
H	-5.87522	-5.95821	1.08515	C	18.96794	-0.24352	1.71678
H	-4.28505	-7.49464	2.16444	C	18.20881	-2.43728	2.3304
H	-1.8471	-6.92464	2.2443	H	16.24401	-2.97722	1.66168
H	-1.07689	-4.76836	1.33738	C	19.1959	-1.44758	2.36329
H	-19.60911	7.2606	0.176	H	19.7181	0.54324	1.78199
H	-24.51162	4.97298	0.50764	H	18.34881	-3.37746	2.86944
H	-25.35217	7.27688	0.58708	H	20.12401	-1.6092	2.91623
H	-23.74082	9.18988	0.51945	C	18.202	2.71275	0.22482
H	-21.31228	8.75376	0.34266	C	19.59044	2.82233	-0.00723
H	-21.33178	-0.57653	0.32916	C	20.28473	4.03252	-0.1574
H	-23.73235	-1.0255	0.84407	O	20.25722	1.66089	-0.10577
H	-24.47735	3.18626	1.01809	C	19.51848	5.22663	-0.09634
H	-25.80408	0.22166	2.22761	B	21.7895	4.06898	-0.37696
H	-26.2747	1.73561	1.42282	C	21.58397	1.55798	-0.37003
H	-26.20356	0.21634	0.50175	C	18.13538	5.1847	0.05408
H	-9.1398	2.45115	-0.14662	N	20.17155	6.42094	-0.21551
H	-10.46537	-2.32344	-0.21851	C	22.41797	5.4893	-0.41209
C	-16.81144	-1.01037	-0.95483	C	22.43678	2.66529	-0.53228
C	-14.87434	-1.91129	0.2558	C	22.04306	0.23868	-0.47334
C	-17.78506	0.01818	-0.99536	C	21.53364	6.59824	-0.34152
C	-18.96789	-0.24343	-1.71725	H	19.60915	7.2606	-0.17545
C	-19.1958	-1.44744	-2.36388	C	23.79307	5.79222	-0.49163
C	-18.2087	-2.43713	-2.33104	C	23.78134	2.36505	-0.84191

C	-17.02639	-2.21838	-1.6444	C	23.37487	0.00471	-0.76003
C	-15.62068	-2.9793	0.78626	C	22.01763	7.9189	-0.38995
C	-14.98415	-4.06744	1.36125	C	24.27782	7.09038	-0.52938
C	-13.58878	-4.10648	1.44177	H	24.51161	4.97321	-0.50932
C	-12.85567	-3.03096	0.96669	C	24.27247	1.07008	-0.95931
C	-13.46056	-1.90719	0.36628	H	24.47787	3.18597	-1.01612
N	-15.56658	-0.81391	-0.30933	H	23.73237	-1.02569	-0.84299
H	-19.71807	0.54331	-1.78239	C	23.37637	8.16031	-0.48674
H	-20.12388	-1.60902	-2.91689	H	21.31214	8.75376	-0.34256
H	-18.34866	-3.37725	-2.8702	H	25.35196	7.27708	-0.58915
H	-16.24393	-2.97711	-1.66225	C	25.71116	0.8008	-1.29202
H	-16.70988	-2.94142	0.76478	H	23.74058	9.19002	-0.52051
H	-15.58457	-4.88114	1.77514	H	25.80542	0.21843	-2.22265
H	-13.08479	-4.95774	1.90479	H	26.2747	1.73528	-1.4225
H	-11.7723	-3.03527	1.09701	H	26.20424	0.21894	-0.49663
C	-0.00001	-2.66799	0.0001	H	17.5505	6.10884	0.0538
C	1.24176	-2.02006	-0.08371	H	21.33154	-0.57654	-0.32941

References

1. Mamada M, Aoyama A, Uchida R, et al. Efficient Deep-blue multiple-resonance emitters based on azepine-decorated *v*-DABNA for CIE_y below 0.06. *Adv. Mater.* 2024; **36**: 2402905.
2. Xiao S, Cao X, Chen G, et al. Synergistic π-extension and peripheral-locking of B/N-based multi-resonance framework enables high-performance pure-green organic light-emitting diodes. *Angew. Chem. Int. Ed.* 2024; **64**: e202418348.
3. Cao X, Huang X, Miao J, et al. Topological structure optimization of B,N-doped nanographenes for deep-blue emitters. *Nat Commun*. 2026; doi:10.1038/s41467-026-73679-9
4. Frisch, M. J. et al. Gaussian 16 Rev. C.01. (Gaussian, Inc., Wallingford CT, 2016).
5. Weigend F, Ahlrichs R. Balanced basis sets of split valence, triple zeta valence and quadruple zeta valence quality for H to Rn: Design and assessment of accuracy. *Phys. Chem. Chem. Phys.* 2005; **7**: 3297-3305.
6. Martin, R. L. Natural transition orbitals. *J. Chem. Phys.* 2003; **118**: 4775-4777.
7. Humphrey, W., Dalke, A. Schulten, K. VMD: Visual molecular dynamics. *J. Mol. Graph.* 1996; **14**: 33-38.
8. Lu T, Chen F. Multiwfn: A multifunctional wavefunction analyzer. *J. Comput. Chem.* 2011; **33**: 580-592.
9. Kondo Y, Yoshiura K. Kitera S. et al. Narrowband deep-blue organic light-emitting diode featuring an organoboron-based emitter. *Nat. Photonics* 2019; **13**: 678-682.
10. Pan KC, Li SW, Ho YY, et al. Efficient and tunable thermally activated delayed fluorescence emitters having orientation-adjustable CN-substituted pyridine and pyrimidine acceptor units. *Adv. Funct. Mater.* 2016; **26**: 7560-7571.
